# Domain Truncation in Hispidin Synthase Orthologs from Non-Bioluminescent Fungi Does Not Lead to Hispidin Biosynthesis

**DOI:** 10.3390/ijms24021317

**Published:** 2023-01-10

**Authors:** Kseniia A. Palkina, Anastasia V. Balakireva, Olga A. Belozerova, Tatiana V. Chepurnykh, Nadezhda M. Markina, Sergey I. Kovalchuk, Aleksandra S. Tsarkova, Alexander S. Mishin, Ilia V. Yampolsky, Karen S. Sarkisyan

**Affiliations:** 1 Shemyakin-Ovchinnikov Institute of Bioorganic Chemistry, Russian Academy of Sciences, 117997 Moscow, Russia; 2 Planta LLC., 121205 Moscow, Russia; 3 Institute of Translational Medicine, Pirogov Russian National Research Medical University, 117997 Moscow, Russia; 4 Synthetic Biology Group, MRC London Institute of Medical Sciences, London W12 0NN, UK; 5 Institute of Clinical Sciences, Faculty of Medicine and Imperial College Centre for Synthetic Biology, Imperial College London, London SW7 2AZ, UK

**Keywords:** bioluminescence, fungi, hispidin, α-pyrones, polyketide synthases, caffeic acid

## Abstract

Hispidin is a polyketide found in plants and fungi. In bioluminescent fungi, hispidin serves as a precursor of luciferin and is produced by hispidin synthases. Previous studies revealed that hispidin synthases differ in orthologous polyketide synthases from non-bioluminescent fungi by the absence of two domains with predicted ketoreductase and dehydratase activities. Here, we investigated the hypothesis that the loss of these domains in evolution led to the production of hispidin and the emergence of bioluminescence. We cloned three orthologous polyketide synthases from non-bioluminescent fungi, as well as their truncated variants, and assessed their ability to produce hispidin in a bioluminescence assay in yeast. Interestingly, expression of the full-length enzyme hsPKS resulted in dim luminescence, indicating that small amounts of hispidin are likely being produced as side products of the main reaction. Deletion of the ketoreductase and dehydratase domains resulted in no luminescence. Thus, domain truncation by itself does not appear to be a sufficient step for the emergence of efficient hispidin synthases from orthologous polyketide synthases. At the same time, the production of small amounts of hispidin or related compounds by full-length enzymes suggests that ancestral fungal species were well-positioned for the evolution of bioluminescence.

## 1. Introduction

Hispidin is a small polyketide found in plants and fungi, with activities in different systems ranging from antioxidant [1,2,3] to cytotoxic [4], potentially hypoglycemic [5], anti-inflammatory [6], antiviral [7], and neuroprotective [8]. It also plays a key role in the bioluminescence of fungi of the order Agaricales, being the biosynthetic precursor of the fungal luciferin.

**Figure 1:**
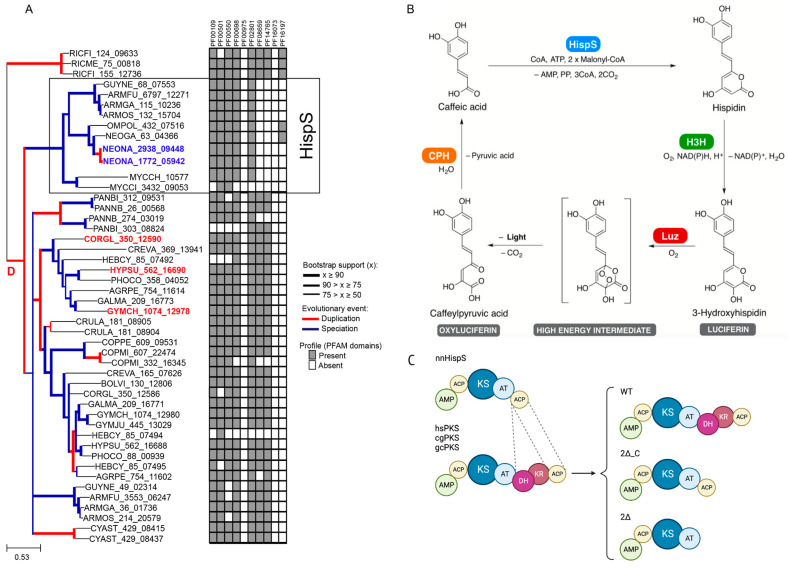
(**A**) Adapted from [9]. Polyketide synthase domain organisation in bioluminescent (marked with a rectangle “HispS”) and non-bioluminescent fungi. Genes selected for domain deletion analysis are shown in red; nnHispS is shown in blue. (**B**) Adapted from [9]. Caffeic acid cycle in bioluminescent fungi: hispidin is produced by a hispidin synthase (HispS), converted by a hispidin-3-hydroxylase (H3H) into luciferin, which is then oxidised by a luciferase (Luz), resulting in emission of light. Oxyluciferin is predicted to be converted back to caffeic acid by a caffeoyl private hydroxylase (CPH). (**C**) Schematic representation of the wild-type (WT) and truncated polyketide synthases from *Cortinarius glaucopus* (cgPKS), *Hypholoma sublateritium* (hsPKS), and *Gymnopilus chrysopellus* (gcPKS). 2Δ denotes enzyme variants with deleted dehydratase, ketoreductase, and C-terminal acyl carrier protein domains; 2Δ_C denotes enzyme variants where only the dehydratase and ketoreductase domains were deleted. KS—ketosynthase, AT—acyltransferase, ACP—acyl carrier protein, AMP—AMP-binding domain, KR—ketoreductase, DH—dehydratase.

**Figure 2:**
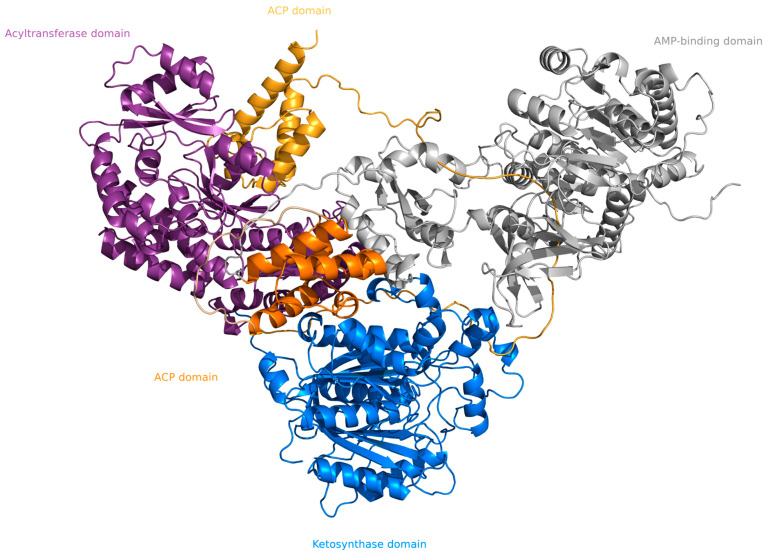
nnHispS protein structure, predicted by AlphaFold 2.0 [10].

In bioluminescent Agaricales, the biosynthesis of hispidin from caffeic acid is carried out by type I polyketide synthases, large enzymes composed of multiple domains with various enzymatic activities. Hispidin synthases from species within the bioluminescent clade are composed of five predicted domains: the AMP-binding domain, the acyl carrier protein domain, the ketosynthase (N- and C-terminal domains), the acyltransferase, and another C-terminal acyl carrier protein domain. At the same time, orthologous enzymes from related non-bioluminescent fungi generally contain two additional domains with predicted ketoreductase and dehydratase activities (Figure 1A and Figure 2).

Such difference in domain architecture made it tempting to speculate that the loss of the domains could have been the key evolutionary event that favoured production of α-pyrones by the ancestral polyketide synthase and, alongside the formation of the luciferase gene cluster [11], might have led to the emergence of bioluminescence in fungi ~160 million years ago [9]. In this study, we aimed to test this hypothesis on several orthologous polyketide synthases from non-bioluminescent fungi.

## 2. Results

To test whether truncation of two domains in hispidin synthase orthologs from non-bioluminescent fungi would lead to hispidin or similar styrylpyrone biosynthesis, we selected three polyketide synthases from closely related non-bioluminescent Agaricales species: *Cortinarius glaucopus* (cgPKS), *Hypholoma sublateritium* (hsPKS), and *Gymnopilus chrysopellus* (gcPKS) (Figure 1A, Supplementary Information S1). The hispidin synthase from the bioluminescent fungus *Neonothopanus nambi* (nnHispS) was used as a control. In the present study, we assessed the effect of deletions of the dehydratase and ketoreductase domains alone (denoted as 2Δ_C on Figure 1C) or together with the C-terminal ACP domain (denoted as 2Δ on Figure 1C), on the ability of the enzymes to produce stytylpyrones suitable for luminescence.

We reconstituted the fungal bioluminescence pathway in yeast to assay for the biosynthesis of hispidin upon treatment with caffeic acid (Figure 1B). We expressed full-length polyketide synthase genes and their truncated versions in a *P. pastoris* strain encoding enzymes required for activity of the fungal bioluminescence pathway in heterologous hosts: luciferase nnLuz, hispidin-3-hydroxylase nnH3H, as well as 4′-phosphopantetheinyl transferase npgA, an enzyme required for activation of polyketide synthases. As CPH is not strictly required for bioluminescence in yeast and plants [9,12] and its role has not yet been experimentally validated, we did not include it in our assay. We confirmed a high level of expression of polyketide synthase genes by real-time PCR (Supplementary Tables S1 and S2).

**Figure 3:**
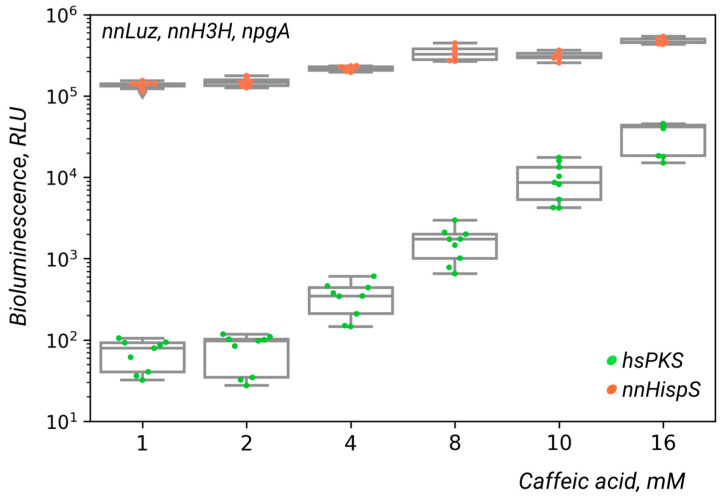
Luminescence emitted by *Pichia pastoris* GS115 yeast colonies expressing nnLuz, nnH3H, npgA, and full-length polyketide synthase from *H. sublateritium* or nnHispS after treatment with different concentrations of caffeic acid.

Surprisingly, we observed dim bioluminescence from yeast expressing full-length polyketide synthase hsPKS from *Hypholoma sublateritium* upon treatment with caffeic acid (Figure 3, WT), suggesting production of hispidin or a related luciferin precursor by this enzyme [13]). However, light intensity was up to two orders of magnitude lower than the control strain expressing hispidin synthase nnHispS from the bioluminescent fungus (Figure 3). In the cases of full-length gcPKS and cgPKS, we detected no luminescence in the presence of caffeic acid (Supplementary Figure S1). To address whether luminescence coincides with hispidin production, we performed LC-MS analysis of yeast strains expressing nnHispS or full-length polyketide synthases grown in the presence or absence of caffeic acid (Supplementary Figure S2). We have shown that nnHispS-expressing glowing yeast indeed produced hispidin, whereas full-length polyketide synthases did not produce hispidin in the samples. Since the hsPKS-expressing strain emits luminescence in the presence of caffeic acid, we suggest that this enzyme is able to produce either hispidin in quantities insufficient for detection by LC-MS or other similar derivatives of caffeic acid with potential for luminescence activity.

All truncated versions lacking dehydratase and ketoreductase domains also demonstrated a complete absence of luminescence indicating no hispidin-like styrylpyrone production from caffeic acid in yeast. Additional truncation of the C-terminal acyl carrier protein did not affect luminescence output either.

Since the luminescence emitted by yeast cells expressing full-length hsPKS was dim compared to those expressing nnHispS, we hypothesised that this enzyme or other assayed polyketide synthases (gcPKS or cgPKS) might prefer substrates other than caffeic acid. To test this, we treated yeast strains expressing full-length enzymes from non-bioluminescent fungi, or nnHispS, with two other tyrosine-derived phenolic acids—*p*-coumaric and ferulic acids.

**Figure 4:**
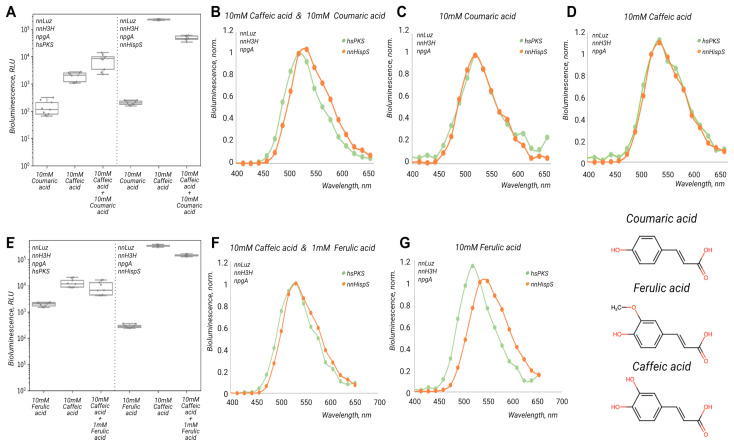
(**A**) Luminescence of *Pichia pastoris* colonies expressing nnLuz, nnH3H, npgA, and either full-length hsPKS (green) or nnHispS (orange), after treatment with coumaric acid, caffeic acid, or their equimolar combination. Bioluminescence spectra were normalised to 518 nm after treatment with (**B**) caffeic and coumaric acids, (**C**) coumaric acid only, and (**D**) caffeic acid only. (**E**) Integral luminescence of yeast colonies after treatment with ferulic acid, caffeic acid, or their equimolar combination. Bioluminescence spectra were normalised on 518 nm after treatment with (**F**) caffeic and ferulic acids, and (**G**) ferulic acid only.

We observed no luminescence in the cases of gcPKS and cgPKS when grown on either 10 mM p-coumaric or 10 mM ferulic acids (Supplementary Figure S1). This result indicated that these polyketide synthases were not able to produce styrylpyrones for bioluminescence reactions. As for hsPKS, we observed different levels of bioluminescence when treated with caffeic acid, coumaric acid, or their equimolar combination (10 mM each) (Figure 4). In the latter case, we detected half an order of magnitude increase in luminescence emitted by hsPKS-expressing cells compared to caffeic acid alone, while in the case of nnHispS the luminescence level remained the same (Figure 4A). Moreover, treatment with coumaric acid resulted in the hypsochromic shift of bioluminescence for both hsPKS and nnHispS (Figure 4B,C). Surprisingly, in the case of an equimolar mixture of the substrates, the bioluminescence spectra for hsPKS corresponded to only coumaric acid, while nnHispS corresponded to only caffeic acid (Figure 4D). This result suggests the preference of hsPKS for coumaric over caffeic acid for the synthesis of hispidin-like compounds, and is interesting in the context of the availability of these substrates in fungal cells, as a related *Hypholoma* species showed up to a 3-fold greater concentration of both ferulic and coumaric acids than caffeic acid [14].

Interestingly, when treated with ferulic acid or its combination with caffeic acid, we did not observe an increase in luminescence compared to caffeic acid alone (Figure 4E). Upon treatment with ferulic acid, both enzymes demonstrated spectral shifts: red-shifted luminescence in the case of nnHispS and blue-shifted, in the case of hsPKS (Figure 4F,G). However, when treated with a 10-to-1 combination of caffeic and ferulic acids both enzymes preferred caffeic acid. At a 10 mM concentration of the substrates, both yeast strains did not survive, while control strains—lacking polyketide synthase and empty strains—grew normally. This result suggests the potential toxicity of combinations of ferulic or caffeic acid-derived alpha-pyrone(s) synthesised by the assayed enzymes.

## 3. Discussion

If the loss of two polyketide synthase domains in the early evolution of bioluminescent fungi favoured hispidin biosynthesis, truncation of these domains in orthologous polyketide synthases can be expected to yield similar results. However, the data presented here indicate that (1) low quantities of similar to hispidin styrylpyrone(s) can be biosynthesised in vivo even by the full-length type I polyketide synthases from related fungal species; and (2) truncation of polyketide synthases from *Cortinarius glaucopus*, *Hypholoma sublateritium*, and *Gymnopilus chrysopellus* does not increase hispidin biosynthesis in *Pichia pastoris*.

Recently, an alternative method of α-pyrone biosynthesis was described [15]. The enzymes, related to those of the fatty acid ß-oxidation pathway in *E. coli*—CoA-ligase FadD and the thiolases FadA and FadI—have been shown to produce styrylpyrones with phenylpropionic acids in vivo using acetyl-CoA as an extender unit for carbon chain elongation through non-decarboxylative Claisen condensation. In this context, it is noteworthy that in our experiments, control yeast strains lacking polyketide synthases were not luminescent; thus, the detected light emission should be attributed to the activity of polyketide synthases.

Both hsPKS, which comprises ketoreductase and dehydratase domains, and nnHispS, which lacks these domains, are able to produce hispidin-like styrylpyrones. Thus, the truncation of the domains was not strictly required to kickstart the biosynthesis of luciferin precursors in fungi. 

Polyketide synthases are sensitive to even small changes in sequence [16], and close polyketide synthase homologs can produce different compounds [17,18]. The mutual orientation of the domains and the linkers between them was shown to be important for the product formation [18,19]. The polyketide synthases selected for the present study differed in the linker sequences between the domains (Supplementary Information S1), which may explain the differences in their ability to produce hispidin-like styrylpyrones. Rational approaches for the engineering of polyketide synthases do not always result in functional enzymes [20], and it is possible that the deletion of the ketoreductase and dehydratase domains in this study may have simply disturbed the structure of the enzymes.

**Figure 5:**
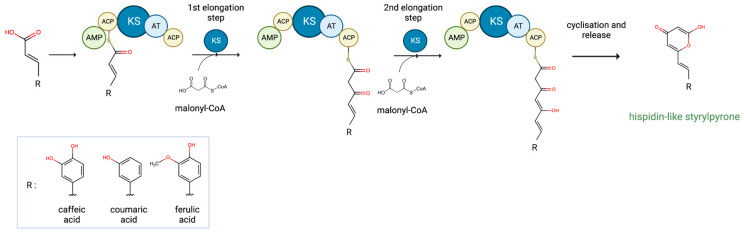
Proposed biosynthetic mechanism of styrylpyrones by nnHispS depending on a substrate. KS—ketosynthase, AT—acyltransferase, ACP—acyl carrier protein, AMP—AMP-binding domain.

**Figure 6:**
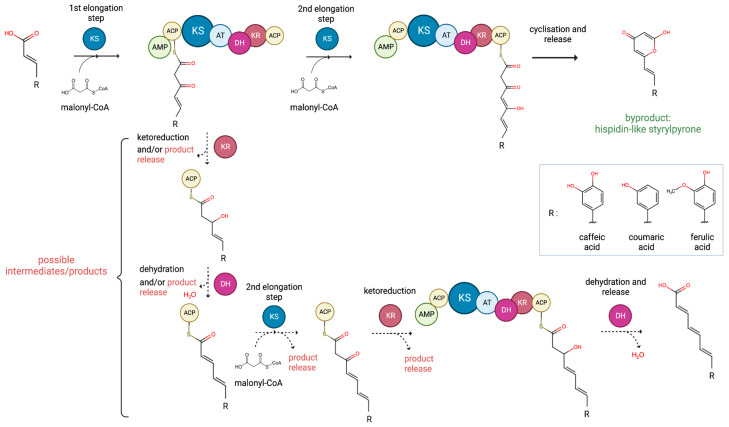
Proposed biosynthetic mechanism of styrylpyrones by hsPKS depending on a substrate. KS—ketosynthase, AT—acyltransferase, ACP—acyl carrier protein, AMP—AMP-binding domain, KR—ketoreductase, DH—dehydratase.

We showed that polyketide synthase from *Hypholoma sublateritium* is able to produce hispidin-like styrylpyrones from caffeic, p-coumaric, and ferulic acids, however, its main reaction product remains unknown. Although the prediction of type I polyketide synthase products from their sequence becomes more and more advanced [21,22,23,24], none of the published tools we tested were able to generate the correct prediction—hispidin—for nnHispS. Thus, we did not consider bioinformatic predictions of reaction products in our analysis of the consequences of domain loss in polyketide synthases. Nevertheless, we attempted to rationally consider reaction products catalysed by nnHisps (Figure 5) or hsPKS (Figure 6) based on existing data on type I polyketide synthase catalysis [20]. 

The dehydratase domain is known to generate α,β-unsaturated bonds through a dehydration reaction [25], while the ketoreductase domain reduces a β-ketone into alcohol using NADPH [26]. It should be noted that the sequence of iterative polyketide synthase protein domains (dehydratase—ketoreductase domains) does not necessarily reflect the order of the corresponding catalysed chemical transformations (dehydration and ketoreduction, respectively), as demonstrated for some evolutionarily related enzymes—fatty acid synthases and other iterative polyketide synthases [27,28,29]. Judging by its domain architecture, nnHispS could be classified as an iterative type I polyketide synthase that is able to perform multiple cycles of carbon chain elongation using malonyl-CoA as an extender unit. Taking this into account, hispidin production by nnHispS could be achieved via two cycles of elongation, cyclisation, and release of the product (Figure 5). As we have shown here, hsPKS is able to produce hispidin-like styrylpyrone as a byproduct, considering the observed dim luminescence. This could be achieved via two cycles of elongation similar to nnHispS, while the formation of the main products might require more than two elongation steps and be accompanied by β-ketone reduction and dehydration events (Figure 6). 

We think that the formation of the main reaction products generated by hsPKS from the phenolic acids used in this study—caffeic, coumaric, and ferulic acids—could occur in a similar way (Figure 5). We used the predicted main products of hsPKS as queries in a structural search for polyketides already described in higher fungi [30]. We could not find exact matches; however, we found several structurally similar compounds. One of the hits was muscarufin, an orange pigment from *Amanita muscaria.* Even though its biosynthesis still remains uncharacterised, it is possibly associated with tyrosine metabolism [31]. The other match was a polyketide isolated from the *Ganoderma lucidum* stem decay fungus [32], a compound that has been assayed for chemotherapy of squamous cell carcinoma—however, its role in fungi and its biosynthesis remain to be explored.

## 4. Materials and Methods

### 4.1. Choice of Polyketide Synthase Genes from Non-Bioluminescent Fungi and Annotation of Domains

Genomes and predicted proteomes of bioluminescent and non-bioluminescent fungi were obtained, as described in [9]. Multiple alignment of selected polyketide synthases was performed using the MUSCLE algorithm [33]. For experiments in this study, we selected three polyketide synthase genes from non-bioluminescent fungi that were phylogenetically closest to *Neonothopanus nambi* hispidin synthase in our dataset but additionally encode dehydratase and ketoreductase domains (Figure 1A). Domain annotation was performed with the HMMER online tool [34].

### 4.2. Cloning

For DNA assembly purposes, polyketide synthase genes from non-bioluminescent fungi were divided into three parts: the first encoding AMP-binding, acyl carrier protein, ketosynthase (N- and C-terminal domains), and acyltransferase domains; the second ketoreductase and dehydratase domains; and the third, the acyl carrier protein domain. We used custom Python scripts to simultaneously codon-optimise polyketide synthase genes for expression in yeast and plant cells. We then ordered optimised genes as synthetic linear DNA and used Golden Gate cloning to assemble vectors for expression in *Pichia pastoris* [35,36,37]. Polyketide synthase genes were expressed under the control of the constitutive GAP promoter and AOX terminator. Zeocin resistance cassette was used as a selectable marker in both *E.coli* and *Pichia* [38]. Primers and plasmid maps used in the study could be found in Supplementary Tables S2 and S3, respectively.

### 4.3. Yeast Transformation

Truncated versions of each polyketide synthase gene — a version lacking ketoreductase and dehydratase domains but containing an intact C-terminal ACP domain (2Δ_C) and a version lacking the C-terminal ACP domain in addition to the ketoreductase and dehydratase domains (2Δ)—were cloned into a vector for expression in yeast *Pichia pastoris* GS115 cells (Figure 1C). We also created a control plasmid carrying hispidin synthase from *N. nambi*. *Pichia pastoris* GS115 yeast strain was transformed by electroporation. The PKSs genes were inserted into the transcriptionally active region of the genome (GAP promoter). Competent cells were thawed on ice and mixed with 0.5–1.0 µg of plasmid DNA, linearised at the AvrII restriction site within the GAP promoter sequence, and column-purified to reduce salt concentration. Cell suspension was incubated on ice for 10 min, transferred to electroporation cuvettes with a 0.2 cm gap (Enfield, CT, Eppendorf, USA), and electroporated on a MicroPulser device (Bio-Rad, Hercules, CA, USA) according to the standard protocol for *P. pastoris*. After electroporation, the cuvette was washed with 1 M sorbitol, and cells were transferred into a clean tube and incubated without shaking for 60 min at 30 °C. After that, 1 mL of YPD without antibiotics was added and incubated with shaking for 60 min at 30 °C. The suspension was then plated onto YPD agar plates containing zeocin (100 µg/mL). All yeast strains obtained in the study were genotyped by PCR reactions using primers specific to polyketide synthase genes (Supplementary Table S2). 

### 4.4. Validation of Gene Expression by RT-PCR

To determine the expression level of target polyketide synthase genes, yeast biomass was flash frozen in liquid nitrogen and homogenised for RNA extraction with the TRIzol kit (Thermo Fisher Scientific, Waltham, MA, USA). Synthesis of the first cDNA strand was carried out with the MMLV kit (Evrogen, Moscow, Russia). RT-PCR was performed with a qPCRmix-HS SYBR + LowROX kit (Evrogen) on a 7500 real-time PCR machine (Applied Biosystems, Waltham, MA, USA) with primers annealing at hsPKS, gcPKS, cgPKS, nnHispS, and actin 1 genes [39] as housekeeping control (primers are listed in Supplementary Table S1) using the following program: 95 °C for 1 min, followed by 40 cycles of 95 °C for 30 s, 60 °C for 30 s, and 72 °C for 30 s. The PCR product size was confirmed by melting curve determination. In total, three technical replicates for assayed polyketide synthases genes and actin 1 control for each strain were analysed.

### 4.5. Yeast “Drop-Test” and Registration of Bioluminescence Spectra

Fresh yeast colonies were transferred into tubes containing 5–7 mL of YPD medium. A 5–10 µL aliquot of each liquid culture was then transferred to a YPD agar plate containing different concentrations of caffeic, ferulic, and coumaric acids dissolved in 40% DMSO and PBS and left to dry at room temperature. After that, the colonies were incubated at 30 °C for 24 h to form large, round “spots” that were imaged using Fusion Pulse.7 (BioRad). The exposure time for imaging yeast spots with *p*-coumaric, ferulic, and caffeic acid was 30 min, except for imaging yeast colonies expressing nnHispS with caffeic acid and its mix with other acids—1 min. Luminescence spectra were registered from yeast suspension in PBS using the TECAN Spark imager. Image analysis was carried out in the Fiji distribution of ImageJ—manually assigned ROI for the spots, subtracted the background, and measured the mean intensity. Figures were created on BioRender.com (accessed on 10 November 2022).

### 4.6. LC-MS Analysis of Yeast Extracts

*P. pastoris* strains expressing hsPKS, gcPKS, cgPKS, or nnHispS along with npgA, nnLuz, and nnH3H were analysed with LC-MS. Yeast colonies were grown in YPD media with or without caffeic acid (10 mM) overnight at 30 °C. After incubation, all samples were washed three times with MQ. In every tube, about 100 mg of glass beads and 1000 µL of cold 70% methanol were added to yeast pellets. The samples were homogenised with a bead mill homogeniser at a frequency of 13.4 rpm for 20 min, then centrifuged for 5 min at 13,000 rpm. A supernatant volume of 700 µL was transferred to another tube and lyophilised in miVac machine. Before analysis, dry residues were reconstituted by vortexing with 100 µL of a 20% acetonitrile-water mixture acidified to 0.1% with formic acid and centrifuged at 13,000 rpm to remove insoluble debris. The supernatants were transferred to HPLC vials and analyzed.

LC-MS analysis was carried out on an Ultimate 3000 RSLCnano HPLC system connected to an Orbitrap Fusion Lumos mass spectrometer (Framingham, MA, Thermo Fisher Scientific), with the loading pump used for analytical flow gradient delivery. Samples were separated on Agilent Eclipse Plus C8 3.5 µm 100 Å column 150 × 2.1 mm at a 200 µL/min flow rate. Separation was performed by a gradient of 99.9% acetonitrile, 10 mM ammonium formate, and 0.1% formic acid (Buffer B) in 99.9% H_2_O, 10 mM ammonium formate, and 0.1% formic acid (Buffer A): 3% B at 0 min, 3% B at 1 min, and 95% B at 11 min, followed by a 2 min wash at 95% B and a 3 min equilibration at 3% B before the next run. MS1 spectra were collected in negative ion mode at 30 K Orbitrap resolution in profile mode with a 150–400 a.e.m mass range and RF lens 30%. For the rest of the MS1 parameters as well as for the ESI parameters, the default values suggested by Xcalibur software ver. 4.3.73.11 were taken. MS2 precursors were selected based on MS1 intensity: Intensity threshold was 5 × 10^4^ with the dynamic exclusion set to 10 s after two selections with a mass tolerance of 10 ppm and isotope exclusion. MS2 spectra were collected at 15 K resolution in centroid mode. The isolation window was set to 1/6 *m*/*z* with no offset and quadrupole isolation mode. Fragmentation was performed by HCD with a stepped CE of 20, 35, and 50%. The rest of the MS2 parameters were taken as default values. The total MS1-MS2 cycle time was selected to be 1 s.

## 5. Conclusions

In this study, we found no evidence that the loss of keto-reductase and dehydratase domains was the key evolutionary event that resulted in hispidin biosynthesis in bioluminescent fungi. However, the demonstration that wild-type orthologous polyketide synthases from non-glowing fungi can produce hispidin-like compounds adds another piece of evidence that ancestral fungal species were well-positioned to evolve light emission via oxidation of styrylpyrones. Future molecular studies are thus more likely to yield insights into the evolution of fungal bioluminescence if they focus on downstream biosynthetic enzymes—hispidin-3-hydroxylase and luciferase.

## Data Availability

Not applicable.

## Supplementary Materials

The following supporting information can be downloaded at: https://www.mdpi.com/article/10.3390/ijms24021317/s1.
