# Supplementary material for: Domain Truncation in Hispidin Synthase Orthologs from Non-Bioluminescent Fungi Does Not Lead to Hispidin Biosynthesis
Source: Int J Mol Sci. 2023 Jan 10;24(2):1317. doi: 10.3390/ijms24021317 (PMC9866795; doi:10.3390/ijms24021317)
Supplement: Supplementary file 1 [file ijms-24-01317-s001.zip › Supplementary Information 1.html]

Launch in Jalview  

{"seqs":[{"start":1,"name":"nnHispS/1-1698","end":1698,"id":"1349904521","seq":"MNSSKN--PPSTLLDVFLDTARNLDTASRNVLECGEHRWSYRELDTVSSALAQHLRYTVGLSPTVAVISENHPYILALMLAVWKLGGTFAPIDVHSPAELVAGMLNIVSPSCLVIPSSDVTNQTLACDLNIPVVAFHPHQSTIPELNKKYLTDS-QISP-DLPFPDPNRPALYLFTSSATSRSNLKCVPLTHTFILRNSLSKRAWCKRMRPETDFDGIRVLGWAPWSHVLAHMQDIGPLTLLNAGCYVFATTPSTYPTE-------------LKDDRDVISCAANAVMYKGVKSFACLPFVLGGLKALCESEPSVKAQLQVEERA---QLLKSLQHMKILECGGAMLEVSVASWAIENRIPISIGIGMTETGGALFAGPVQAI-----QTGFSSEDKFIEDATYLLVKDDYESHAEED--INEGELVVKSRMLPRGYLGYNDPSFSVDDAGWVTFKTGDRYSVTPDGKFSWLGRNTDFIQMTSGETLDPRPIESLLCESSLISRACVIGDKFLNGPATAVCAIIELEPTT-VEKGQ---AHSRDIARIFAPINRDLPPPLRIAWSHVLVLQPSEKIPMTKKGTIFRKKIEQVFGSALGGSSG-----------------DNS-----QATTDASVVRRDE-LSNTVKHIISRVLGVSDDELL--WTLSFAELGMTSALATRIANELNEVLVGVNLPINACYIHVDLPSLSNAVYAKLAHLKLPDRTPEPRKAPVENPGGKEIVIVGQAFRLPGSINDVASLRDAFLARQASSIITEIPPDRWDHASFYP----------KDIRFNKAGLVDIANYDHSFFGLTATEALYLSPTMRLALEVSFEALENANIPVSQLKGSQTAVYVAT-TDDGFETLLNAEAGYDAYTRFYGTGRAASTASGRISYLLDVHGPSITVDTACSGGAVCIDQAIDYLQSSSAADTAIICASNTHCWPGSFMFLSAQGMVSSGGRCATFTTDADGYVPSEGAVAFILKTREAAMRDKDTILATIKATQISHNGRSQGLVAPNVNSQADLHRSLLQKAGLSPADIHFIEAHGTGTSLGDLSEIQAINDAYTSSQPRTAGPLIVSASKTVIGHTEPAGPLVGMLSVLNSFKEGAVPGLAHLTADNLNPALDCSSVPLLIPYQPVHLA---APKPHRAAVMSYGFSGTLGGIVLEAPDE-----E---RLEEEPPNDKPMLFVVSAKTHTALIEYLGRYLEFLLQANPQDFCDICYTSCVGREHYRYRFACVANDMEDLIGQLQKRLGSKVP--PKPSY-KRGALAFAFSGQGTQFRGMATELAKAYSGFRKIVSDLAKRASELSGHAIDRFLLAYDIGAENVAPDSEADQICIFVYQCSVLRWLQTMGIRPSAVIGHSLGEISASVAAGALSLDSALDLVISRARLLRSSTNAPAGMAAMSASQDEVVELIGKLDLDKANSLSVSVINGPQNTVVSGSSAAIESIVALAKGRKIKASALNINQAFHSPYVDSAVPGLRAWSEKHISSARPLQIPLYSTLLGAQVSEGQMLNPDHWVDHARKPVQFAQAATIM-KESFTGVIIDIGPQVVAWSLLLSNGLT-SV-TALAAKRGRSQQVAFLSALADLYQDYGVVPDFVGLYAQQEDASRLKKTDILTYPFQRVRRYPSFIPSRRAP------------------------------------------------------------------------------------------------------------------------------------------------------------------------------------------------------------------------------------------------------------------------------------------------------------------------------------------------------------------------------------------------------------------------------------------------------------------------------------------------------------------------------------------------------------------------------------------------------------------------------------------------------------------------------------------------------------------------------------------------------------------------------------------------THAHVQDEETLSSGSSTPTLENTD-----LDSGKESLMG-PTRGLLRVDDLRDSIVSSVKDVLELKSNEDLDLS------------------ESLNALGMDSIMFAQLRKRIGEGLGLSVPMVFLSDAFSIGEMVSNLVEQAEASEDN-","order":1},{"start":1,"name":"gcPKS(wt)/1-2484","end":2484,"id":"1630866817","seq":"MDE------VKTLLDAFLSVARNPSTIEDPVLECGSEQWSYGDLDCISSGLALKIHQKCGLKPMVAVISENHPFVLAILLATWKLGGIFAPFDCHSPLEMVEKMLENTEATCAVVPDFEDGLNGLLDKMQIPKISYS-KNTTITSLTQLYLAQVAEISPSLYPPPDLASLAAYIHTSSASSISNLKCVQLTHESIAYGCRSVIKWFHRAWPSVNFDKLRVLGIAPWSHIMALSYDFGAATFGTGGCYVFGVPPSGYPVGIEVGHVDDSVSGKAEEERDILDRLVDAAVKARPDVLVAVPWVLEGFKERYTRLLG------ANKEAEALRVKYALQALKCLGSGGAAMSAEMLSWIKELKINASSNIGMTELGGGLFHRKIDLLSTPDHDDGWSLEDCFFDDVQLVLVDEDG----TEN--DQEGELIITTRHISRGYLKYDNSAFSFLPDGSTTFRTGDVYERKSDGRIVWKGRKDDYIQTASGETLDPRPIEKALSACEGVLHCCVVGNNFMRKASDAICILIEPAVTADGDSVTLTTTEIAQITKTLAAMNRGLLPPLRIPWSRVVVLEKGMRIPYTRKGMIFRKKLESLFGDVVSHLLEKDNLGHLALNGEINYEEDKK----PAKPSAPGIWKTED-VKSMTVVTIASILGVGTEALRAAPDTTFAEFGMDSNMAVRIVNELNSTF-SLHLPLNACHTSVDLQSMCEAIMVELGIKGEQQASSGLCDQPTTTNVKEEVVIVGQALRLPGDINTPDAFWEALINKR-NDIMIPVPPDRWDHASFYRSPTSPEPPQIGDINFEKAGFLDVAHFDNAFFGISTPEAFFVSPSVRLTLETAFEALENANIPISKVKGTSMGAFVAAGLNEGYTHVLFTSLGWEAYKRTFGTGTASSTACGRLSYLLDIHGPSMTTDTACSSGLVAFDQAVKYIQSGG-GESAMVSAASTALWPGSFGFLSANKMASVNSRCATFTTEADGYVPSEGCVAFVLKSKTAALRDGDNILAIVKSTEVMHGGKSQGLVSPNVKTQIALQRSLLVQAGLQPSEIAFLEAHGTGTSLGDLIEIQGINEVFKYSHT--EDPLILGASKSCIGHTEMASGLVGLLSAIASLKYGVVPGLVHLNEHNLNPSIDCDMVPLHIPHEVAPLP-RMPETPSRGLILSNGFAGTLAGAIIEGPGNAL-SGDLQKKQFSALNDQIPMPFVVSAKTPERLCAYMEKYIAFCRKSTPSDFVNLCFTTCVGREHYRYRFACVAHNLPELIATLESRLQELRYNAQAIGNVAGPRVAFAFPGQGSQFQGMASELAEHFPDFKEIISALSVEASQVSGYDILSFLLDKDSPCEFLVNEGRMGQIGIFVFQSSLASWLRSLGIEPFAVLGHSLGEIAATVTAGAMDYAFALKFVIRRAEILCPEFAQSAGMALIAASEETILQRLHELGLED--HLAIAVCNGPNSHAVSGNLLAIDSLVTDAKAQGIRATKLNVTQGFHSPSIYPYLPVLEAWLAGNQHELAPLKLPMYSTVYGRKIAADSELATSYWIEHAKNPVEFYRAVRELENDKDLNIILDIGPQPFIWTTLQTLQHH-KATISTSTKQPQSQNLAFLRGIALLFE-QGVTPNFEKLLQGS-R-SRGRKISVPTYPFQKQRHYPECIPSRYHVPK----------GSTVDRVLEFPIDQGLFDLLADHLIQGHRVVPAAYLIDFFANKAPSGALQSISFHLPLVLESYDLTVNAEINGNGRFLLYNSDSSGQHVCSGILGSRVPPVLKNG-VRDQ---PPEQIKDTADVYASFKNVQFGPSFRNVQEIRTWPTHADALIAVHSAAHRAPSLDRIRKLDACLHSLGAIITREVPQVRELDGAFLPSSLEGFSLHSDDLPESFVCRYYLPVDVARNYHVISAAFDVFSEAGELLVSCKKYSVAWIPTGVTIQKSERNSGSTSEAQSVGWWRQTWVNKGTATE-DIGFSKYDQLLAISPTERDSRIASLANGIS----------------------------KEVSLP---DPLEEVIQKFKDSPAVIMLDLTSIDALPMTEAFTSCYRHVLRLMQLLSSHKIDIREFVVISATSVAADVPA------GPSAPEVEAPSVAALVQGMMRVFRRETGSDDQIWALDLPRLDTISDDALRHLLLSELEGRQRGKHTDRAVAYRRRDGQEKVERLVPIFESVKADEEVGTTYHGVAVITGLGSIGASLAQPMVMKGSSKVVYIGRRPVDDTEVQTTLHRLESEIPGRIAYVQADVCELDSLKSAISDIQASHGPIGSIIHSAAVISDATIQNIDLQSFETVIRPKVVGAWNLHVLNEELCPTLADFVLLSSISVSLGNPGQAAYAAANHYIEVLASYRRGKNLPATALQLGPWESKLTQNLDTRDSLIPLMNNKRGVPLIISAMSKSDSVQMIANLDARKLAAHPVYSRDPLFYDIVFPEQDRLAK-SHQAR-SDEEISTLVANILRKVLELRPSEKLGQSLNLLLLAQLFDNRTAELDDSLTICGVDSISFAQVRGRILHELMVEVPMMFLSDTFTIHEMIAFVIEKYSSRIASL","order":2},{"start":1,"name":"gcPKS(Δ2)/1-1572","end":1572,"id":"1200812152","seq":"MDE------VKTLLDAFLSVARNPSTIEDPVLECGSEQWSYGDLDCISSGLALKIHQKCGLKPMVAVISENHPFVLAILLATWKLGGIFAPFDCHSPLEMVEKMLENTEATCAVVPDFEDGLNGLLDKMQIPKISYS-KNTTITSLTQLYLAQVAEISPSLYPPPDLASLAAYIHTSSASSISNLKCVQLTHESIAYGCRSVIKWFHRAWPSVNFDKLRVLGIAPWSHIMALSYDFGAATFGTGGCYVFGVPPSGYPVGIEVGHVDDSVSGKAEEERDILDRLVDAAVKARPDVLVAVPWVLEGFKERYTRLLG------ANKEAEALRVKYALQALKCLGSGGAAMSAEMLSWIKELKINASSNIGMTELGGGLFHRKIDLLSTPDHDDGWSLEDCFFDDVQLVLVDEDG----TEN--DQEGELIITTRHISRGYLKYDNSAFSFLPDGSTTFRTGDVYERKSDGRIVWKGRKDDYIQTASGETLDPRPIEKALSACEGVLHCCVVGNNFMRKASDAICILIEPAVTADGDSVTLTTTEIAQITKTLAAMNRGLLPPLRIPWSRVVVLEKGMRIPYTRKGMIFRKKLESLFGDVVSHLLEKDNLGHLALNGEINYEEDKK----PAKPSAPGIWKTED-VKSMTVVTIASILGVGTEALRAAPDTTFAEFGMDSNMAVRIVNELNSTF-SLHLPLNACHTSVDLQSMCEAIMVELGIKGEQQASSGLCDQPTTTNVKEEVVIVGQALRLPGDINTPDAFWEALINKR-NDIMIPVPPDRWDHASFYRSPTSPEPPQIGDINFEKAGFLDVAHFDNAFFGISTPEAFFVSPSVRLTLETAFEALENANIPISKVKGTSMGAFVAAGLNEGYTHVLFTSLGWEAYKRTFGTGTASSTACGRLSYLLDIHGPSMTTDTACSSGLVAFDQAVKYIQSGG-GESAMVSAASTALWPGSFGFLSANKMASVNSRCATFTTEADGYVPSEGCVAFVLKSKTAALRDGDNILAIVKSTEVMHGGKSQGLVSPNVKTQIALQRSLLVQAGLQPSEIAFLEAHGTGTSLGDLIEIQGINEVFKYSHT--EDPLILGASKSCIGHTEMASGLVGLLSAIASLKYGVVPGLVHLNEHNLNPSIDCDMVPLHIPHEVAPLP-RMPETPSRGLILSNGFAGTLAGAIIEGPGNAL-SGDLQKKQFSALNDQIPMPFVVSAKTPERLCAYMEKYIAFCRKSTPSDFVNLCFTTCVGREHYRYRFACVAHNLPELIATLESRLQELRYNAQAIGNVAGPRVAFAFPGQGSQFQGMASELAEHFPDFKEIISALSVEASQVSGYDILSFLLDKDSPCEFLVNEGRMGQIGIFVFQSSLASWLRSLGIEPFAVLGHSLGEIAATVTAGAMDYAFALKFVIRRAEILCPEFAQSAGMALIAASEETILQRLHELGLED--HLAIAVCNGPNSHAVSGNLLAIDSLVTDAKAQGIRATKLNVTQGFHSPSIYPYLPVLEAWLAGNQHELAPLKLPMYSTVYGRKIAADSELATSYWIEHAKNPVEFYRAVRELENDKDLNIILDIGPQPFIWTTLQTLQHH-KATISTSTKQPQSQNLAFLRGS---------------------------------------------------------------------------------------------------------------------------------------------------------------------------------------------------------------------------------------------------------------------------------------------------------------------------------------------------------------------------------------------------------------------------------------------------------------------------------------------------------------------------------------------------------------------------------------------------------------------------------------------------------------------------------------------------------------------------------------------------------------------------------------------------------------------------------------------------------------------------------------------------------------------------------------------------------------------------------------------------------","order":3},{"start":1,"name":"gcPKS(Δ2&C)/1-1755","end":1755,"id":"1012189","seq":"MDE------VKTLLDAFLSVARNPSTIEDPVLECGSEQWSYGDLDCISSGLALKIHQKCGLKPMVAVISENHPFVLAILLATWKLGGIFAPFDCHSPLEMVEKMLENTEATCAVVPDFEDGLNGLLDKMQIPKISYS-KNTTITSLTQLYLAQVAEISPSLYPPPDLASLAAYIHTSSASSISNLKCVQLTHESIAYGCRSVIKWFHRAWPSVNFDKLRVLGIAPWSHIMALSYDFGAATFGTGGCYVFGVPPSGYPVGIEVGHVDDSVSGKAEEERDILDRLVDAAVKARPDVLVAVPWVLEGFKERYTRLLG------ANKEAEALRVKYALQALKCLGSGGAAMSAEMLSWIKELKINASSNIGMTELGGGLFHRKIDLLSTPDHDDGWSLEDCFFDDVQLVLVDEDG----TEN--DQEGELIITTRHISRGYLKYDNSAFSFLPDGSTTFRTGDVYERKSDGRIVWKGRKDDYIQTASGETLDPRPIEKALSACEGVLHCCVVGNNFMRKASDAICILIEPAVTADGDSVTLTTTEIAQITKTLAAMNRGLLPPLRIPWSRVVVLEKGMRIPYTRKGMIFRKKLESLFGDVVSHLLEKDNLGHLALNGEINYEEDKK----PAKPSAPGIWKTED-VKSMTVVTIASILGVGTEALRAAPDTTFAEFGMDSNMAVRIVNELNSTF-SLHLPLNACHTSVDLQSMCEAIMVELGIKGEQQASSGLCDQPTTTNVKEEVVIVGQALRLPGDINTPDAFWEALINKR-NDIMIPVPPDRWDHASFYRSPTSPEPPQIGDINFEKAGFLDVAHFDNAFFGISTPEAFFVSPSVRLTLETAFEALENANIPISKVKGTSMGAFVAAGLNEGYTHVLFTSLGWEAYKRTFGTGTASSTACGRLSYLLDIHGPSMTTDTACSSGLVAFDQAVKYIQSGG-GESAMVSAASTALWPGSFGFLSANKMASVNSRCATFTTEADGYVPSEGCVAFVLKSKTAALRDGDNILAIVKSTEVMHGGKSQGLVSPNVKTQIALQRSLLVQAGLQPSEIAFLEAHGTGTSLGDLIEIQGINEVFKYSHT--EDPLILGASKSCIGHTEMASGLVGLLSAIASLKYGVVPGLVHLNEHNLNPSIDCDMVPLHIPHEVAPLP-RMPETPSRGLILSNGFAGTLAGAIIEGPGNAL-SGDLQKKQFSALNDQIPMPFVVSAKTPERLCAYMEKYIAFCRKSTPSDFVNLCFTTCVGREHYRYRFACVAHNLPELIATLESRLQELRYNAQAIGNVAGPRVAFAFPGQGSQFQGMASELAEHFPDFKEIISALSVEASQVSGYDILSFLLDKDSPCEFLVNEGRMGQIGIFVFQSSLASWLRSLGIEPFAVLGHSLGEIAATVTAGAMDYAFALKFVIRRAEILCPEFAQSAGMALIAASEETILQRLHELGLED--HLAIAVCNGPNSHAVSGNLLAIDSLVTDAKAQGIRATKLNVTQGFHSPSIYPYLPVLEAWLAGNQHELAPLKLPMYSTVYGRKIAADSELATSYWIEHAKNPVEFYRAVRELENDKDLNIILDIGPQPFIWTTLQTLQHH-KATISTSTKQPQSQNLAFLRGSGG-----------------------------------------------------------------------------------------------------------------------------------------------------------G-------------------------------------------------------------------------------------------------------------------------------------------------------------------------------------------------------------------------------------------------------------------------------------------------------------------------------------------------------------------------------------------------------------------------------------------------------------------GSGGGGSG----------------------------------------------------------------------------------------------------------------------------------------------------------------------------------GGGSLIPLMNNKRGVPLIISAMSKSDSVQMIANLDARKLAAHPVYSRDPLFYDIVFPEQDRLAK-SHQAR-SDEEISTLVANILRKVLELRPSEKLGQSLNLLLLAQLFDNRTAELDDSLTICGVDSISFAQVRGRILHELMVEVPMMFLSDTFTIHEMIAFVIEKYSSRIASL","order":4},{"start":1,"name":"cgPKS(wt)/1-2421","end":2421,"id":"2143416565","seq":"MFCSISPPSPQTLLDSFLYAARNVETVENDVVECGYEKWSYGDLDVISTGLAIEIKETYGMKPTVATFSENHPYILAVMLATWKLGGICAPLDHHTPHELVQHMIINIAPTFVVVPSSDEPIKQLLQGMNVHFMIFDVRTTSMTSLTQRFLNQSPDLSVEAFPLPSPSDIAFFLHTSSASSISNLKCVPLAHGSVFSGCKSRLTWWQKTWPDKNFMNLRVLGWSPWSHILGISHDIGGATFATAGCYLFGLIPSSYTSQQEL-------TDEYEGEFDIVSRLLNAVIRLRPDVFSAVPWVLEGFRDKWSRETV------ADKKR---VMQDVLEKMKVFGCGGAALSKEIVLWAKDMNIPVTVDIGMTELGRPLFYSKADDFD----LLGWSMKDCLIPDAELRLVNENC----DDDDDLEEGELVITSGAISQGYLKFDNLAFTKAPDGRTTFRTGDVYTMTADDHLMWIGRKEDYIQMVSGETLDPRPIERALNTSSAISHSCVIGNHFLGRAAGFICVLIKPANNKAIQKTPSNAIITSEITRAVASVNRTLPPPLRIAWSRVLILDEGQEVPYTRKGTIFRKKLEGMFGGQVAGLLENGDV-------QDNQEKLAKVSDYHQEGTASSKWTKVD-VTDMVLKTVAGALQISIAVLSMHSDSSFIEFGMDSNMAVRIVNELNHLF-KLQLPLNTCHTYLDLVSLSGAVLTELGMNEKVTIEV-STTNPPTVQSHEEVVIVGQALRLPGDINTPESFWQALVDKR--DIMTPIPQDRWDQASFYRSPSSTAPPQDCDITFEKAGFIQVESFDNSFFGISTPEAFYVSPTIRLTLETAFEALENANIPVSRVKGTNMGIFVAAGLDGGYQQLLYYDQGFGAYTRFFGTGIATSTACGRLSYLLDVHGPSITSDTACSSGLVVFDQAVKYLQSGD-GESAIVCAVNTNLWPGSFGFLSAQKMASPHSRCATFSSEADGYVSSEGAVAFILKTRSAALRDGDSILAVVKSTDIKHGGRSQGLVSPNVNAQIALQSSLLEKAGLKPSEIDFVEAHGTGTSLGDLIEIQGINQVFQRSHS--ETPLIVGAAKSCIGHTEVAAGLVGVLKAIASFRHSAIPGLMHLTAENMNPSIDCGIIPMHIPYELFPLPPKENNTPYRSVVLANGFAGTIAGVILEDPKHVIHSADLNSINEAETSEDFPMLFVVSAKSAEALTQYLWKYLDFCRTSSTSDFRSICYTTCLGREHYRYRFACVVGSMGSLIKVLENRLRSTSS--PVASNPAACRIAFAFPGQGSHYQAMASDLVTRYPGFKDILDSAASTASTLSGYPISSFLVDAKTSCDLAIDNSQVAQICIFVYQYSICTWLKQLGIEPRAVLGHSLGEIAAAVIGGALPYEIGLNLVVTRARLLQCDPTHPGGMAIVGASQERILYIIHKLGLDD--RLVIAVYNDPENHVISGEIKAIDTFLSTANIWGFRGTKINVDQG--APCISSALPALQEWVSGHRHLSSPLNIPLYSTVYGKEIRGNQWLSPDYWVEHARDPVRFFDAVEALYSSKSFDIIVDIGPQPLIWTTLQSFSRK-NIAIATCGKRSNDQNAAFLGAIASLFE-MDIAPDFGKLLAHKSQ-YGGHMCSLPTYPFQRQRHYPTSIPSRNSPPPVLLPLLPVSHTNPSSSIIHFNVDQSLCDLLLDHRIEGHRVAPGASLVDFFAKLCPVKSVKTIKFNAPLVLDFPEAHVVAEFTDGHHFAMYDNHSRTHIVCSGIAASRAPQSYTRQPINLNPSEQPEQVLTKDEIYKVFKNVEFGSAFRNIQEYRRWSSHADCLITVEPTEH--PAHDRIRKLDSCLHMFGAFSFQEVPQSRDLDGAFLPTALEDFTLHSDELPSSFICRYYLPLDVSRNFHVMSAAFDVFSLAGALLVSCRKYSVAWIPVGIAIPNHI-S----QPSVNTQWLQQSWVARDLPSA--VPDDKLEVLCVID--QAQSQIPSLFNRMAWKT-------------HFLNLYDLLAPSQSFSLPSLSLQLHAIIDRITSTNLLIVVDVTSNHASPTSEAFCSSHRHILTLMKLLISSKVRFTSLVFITEMSVAIGNEG--NRLLAPHLLASMTPTVGSVIQGMLRVFRREMGLDEVIWALDLPPMNTVEDGVILGIISNEICSRLHGLSTDRTVAYRNVDQTKSLSRLVPVLQSIGHH--PIREVSGTSIVVGLGSIGHALAPSLA-GSHSQVVFIGRRQAHDHEVQEVLLHLQSKTGGRCAYMQADVCDPDSLRNVIISAQTLYGPIENIVHTAVVVSDATIQTVADKSFELVLRPKVIGAWNLHTICEELKLPLKSFVLLSSVSVPLGNQGQIAYVAGNAYMETLASYRHSVGLPATCLQLGAWESKLVQNLDFSTGFVRPIKHAKGIPLLLKAMLTPIAVQVIADFDVEKLASVPAYARDPLFCHILGGAALTQIKTSLRGNLTEDEVADIMIDILRTVLELRPSERLG-----------------------------------------------------------------------------","order":5},{"start":1,"name":"cgPKS(Δ2)/1-1584","end":1584,"id":"1712358427","seq":"MFCSISPPSPQTLLDSFLYAARNVETVENDVVECGYEKWSYGDLDVISTGLAIEIKETYGMKPTVATFSENHPYILAVMLATWKLGGICAPLDHHTPHELVQHMIINIAPTFVVVPSSDEPIKQLLQGMNVHFMIFDVRTTSMTSLTQRFLNQSPDLSVEAFPLPSPSDIAFFLHTSSASSISNLKCVPLAHGSVFSGCKSRLTWWQKTWPDKNFMNLRVLGWSPWSHILGISHDIGGATFATAGCYLFGLIPSSYTSQQEL-------TDEYEGEFDIVSRLLNAVIRLRPDVFSAVPWVLEGFRDKWSRETV------ADKKR---VMQDVLEKMKVFGCGGAALSKEIVLWAKDMNIPVTVDIGMTELGRPLFYSKADDFD----LLGWSMKDCLIPDAELRLVNENC----DDDDDLEEGELVITSGAISQGYLKFDNLAFTKAPDGRTTFRTGDVYTMTADDHLMWIGRKEDYIQMVSGETLDPRPIERALNTSSAISHSCVIGNHFLGRAAGFICVLIKPANNKAIQKTPSNAIITSEITRAVASVNRTLPPPLRIAWSRVLILDEGQEVPYTRKGTIFRKKLEGMFGGQVAGLLENGDV-------QDNQEKLAKVSDYHQEGTASSKWTKVD-VTDMVLKTVAGALQISIAVLSMHSDSSFIEFGMDSNMAVRIVNELNHLF-KLQLPLNTCHTYLDLVSLSGAVLTELGMNEKVTIEV-STTNPPTVQSHEEVVIVGQALRLPGDINTPESFWQALVDKR--DIMTPIPQDRWDQASFYRSPSSTAPPQDCDITFEKAGFIQVESFDNSFFGISTPEAFYVSPTIRLTLETAFEALENANIPVSRVKGTNMGIFVAAGLDGGYQQLLYYDQGFGAYTRFFGTGIATSTACGRLSYLLDVHGPSITSDTACSSGLVVFDQAVKYLQSGD-GESAIVCAVNTNLWPGSFGFLSAQKMASPHSRCATFSSEADGYVSSEGAVAFILKTRSAALRDGDSILAVVKSTDIKHGGRSQGLVSPNVNAQIALQSSLLEKAGLKPSEIDFVEAHGTGTSLGDLIEIQGINQVFQRSHS--ETPLIVGAAKSCIGHTEVAAGLVGVLKAIASFRHSAIPGLMHLTAENMNPSIDCGIIPMHIPYELFPLPPKENNTPYRSVVLANGFAGTIAGVILEDPKHVIHSADLNSINEAETSEDFPMLFVVSAKSAEALTQYLWKYLDFCRTSSTSDFRSICYTTCLGREHYRYRFACVVGSMGSLIKVLENRLRSTSS--PVASNPAACRIAFAFPGQGSHYQAMASDLVTRYPGFKDILDSAASTASTLSGYPISSFLVDAKTSCDLAIDNSQVAQICIFVYQYSICTWLKQLGIEPRAVLGHSLGEIAAAVIGGALPYEIGLNLVVTRARLLQCDPTHPGGMAIVGASQERILYIIHKLGLDD--RLVIAVYNDPENHVISGEIKAIDTFLSTANIWGFRGTKINVDQG--APCISSALPALQEWVSGHRHLSSPLNIPLYSTVYGKEIRGNQWLSPDYWVEHARDPVRFFDAVEALYSSKSFDIIVDIGPQPLIWTTLQSFSRK-NIAIATCGKRSNDQNAAFLGAIASLFE-MDIAPDFGKLLAHKSQ-YGG-------------------------------------------------------------------------------------------------------------------------------------------------------------------------------------------------------------------------------------------------------------------------------------------------------------------------------------------------------------------------------------------------------------------------------------------------------------------------------------------------------------------------------------------------------------------------------------------------------------------------------------------------------------------------------------------------------------------------------------------------------------------------------------------------------------------------------------------------------------------------------------------------------------------------------------------------------------------------------","order":6},{"start":1,"name":"cgPKS(Δ2&C)/1-1695","end":1695,"id":"919708553","seq":"MFCSISPPSPQTLLDSFLYAARNVETVENDVVECGYEKWSYGDLDVISTGLAIEIKETYGMKPTVATFSENHPYILAVMLATWKLGGICAPLDHHTPHELVQHMIINIAPTFVVVPSSDEPIKQLLQGMNVHFMIFDVRTTSMTSLTQRFLNQSPDLSVEAFPLPSPSDIAFFLHTSSASSISNLKCVPLAHGSVFSGCKSRLTWWQKTWPDKNFMNLRVLGWSPWSHILGISHDIGGATFATAGCYLFGLIPSSYTSQQEL-------TDEYEGEFDIVSRLLNAVIRLRPDVFSAVPWVLEGFRDKWSRETV------ADKKR---VMQDVLEKMKVFGCGGAALSKEIVLWAKDMNIPVTVDIGMTELGRPLFYSKADDFD----LLGWSMKDCLIPDAELRLVNENC----DDDDDLEEGELVITSGAISQGYLKFDNLAFTKAPDGRTTFRTGDVYTMTADDHLMWIGRKEDYIQMVSGETLDPRPIERALNTSSAISHSCVIGNHFLGRAAGFICVLIKPANNKAIQKTPSNAIITSEITRAVASVNRTLPPPLRIAWSRVLILDEGQEVPYTRKGTIFRKKLEGMFGGQVAGLLENGDV-------QDNQEKLAKVSDYHQEGTASSKWTKVD-VTDMVLKTVAGALQISIAVLSMHSDSSFIEFGMDSNMAVRIVNELNHLF-KLQLPLNTCHTYLDLVSLSGAVLTELGMNEKVTIEV-STTNPPTVQSHEEVVIVGQALRLPGDINTPESFWQALVDKR--DIMTPIPQDRWDQASFYRSPSSTAPPQDCDITFEKAGFIQVESFDNSFFGISTPEAFYVSPTIRLTLETAFEALENANIPVSRVKGTNMGIFVAAGLDGGYQQLLYYDQGFGAYTRFFGTGIATSTACGRLSYLLDVHGPSITSDTACSSGLVVFDQAVKYLQSGD-GESAIVCAVNTNLWPGSFGFLSAQKMASPHSRCATFSSEADGYVSSEGAVAFILKTRSAALRDGDSILAVVKSTDIKHGGRSQGLVSPNVNAQIALQSSLLEKAGLKPSEIDFVEAHGTGTSLGDLIEIQGINQVFQRSHS--ETPLIVGAAKSCIGHTEVAAGLVGVLKAIASFRHSAIPGLMHLTAENMNPSIDCGIIPMHIPYELFPLPPKENNTPYRSVVLANGFAGTIAGVILEDPKHVIHSADLNSINEAETSEDFPMLFVVSAKSAEALTQYLWKYLDFCRTSSTSDFRSICYTTCLGREHYRYRFACVVGSMGSLIKVLENRLRSTSS--PVASNPAACRIAFAFPGQGSHYQAMASDLVTRYPGFKDILDSAASTASTLSGYPISSFLVDAKTSCDLAIDNSQVAQICIFVYQYSICTWLKQLGIEPRAVLGHSLGEIAAAVIGGALPYEIGLNLVVTRARLLQCDPTHPGGMAIVGASQERILYIIHKLGLDD--RLVIAVYNDPENHVISGEIKAIDTFLSTANIWGFRGTKINVDQG--APCISSALPALQEWVSGHRHLSSPLNIPLYSTVYGKEIRGNQWLSPDYWVEHARDPVRFFDAVEALYSSKSFDIIVDIGPQPLIWTTLQSFSRK-NIAIATCGKRSNDQNAAFLGAIASLFE-MDIAPDFGKLLAHKSQ-YGGS--------------------------------------------------------------------------------------------------------------------------------GG-------------------------------------------------------------------------------------------------------------------------------------------------------------------------------------------------------------------------------------------------------------------------------------------------------------------------------------------------------------------------------------------------------------------------------------------------------------------GGSGGGGSGG--------------------------------------------------------------------------------------------------------------------------------------------------------------------------------GGSTGFVRPIKHAKGIPLLLKAMLTPIAVQVIADFDVEKLASVPAYARDPLFCHILGGAALTQIKTSLRGNLTEDEVADIMIDILRTVLELRPSERLG-----------------------------------------------------------------------------","order":7},{"start":1,"name":"hsPKS(wt)/1-2457","end":2457,"id":"493519005","seq":"MAASLY--DKNTLLNAFLGVAHSAD-VDRNAVEYGNERWTYGDLDTVSTGLALEMHKKYGPKPVVAIVSENHPYTLAMLFAIWKLGGIAAPLDHNVPKDIMERMLLNIGPTCVLVPATERVVQSIVEGISVACHAFNPKEMSITALMQKYLDLSPELTGPAFHLPNPDDIALYLHTSSASSVANVKCVPTTHASILGASAARLAWWKRTWPAQQYTHLRVLGWSTWAHVIGLTNDLGAAMVLTAGCYIFAMPPASGAGGNA-----------AALYLDVCGQLLETAIIKQPTVFAGVPWVLEGFMRNYKQEAD------AARKQ---AIQDAVKRLKVFGSGGASTNAECIEWAIQMAIPLVLDIGMTEVGGPLFHSTIGG------PEGWLSEDCMLPGAQLKLIDDSG----AEV--STEGELVVRAQNVTRGYRHYDNSSFTLENDGTVSFKTGDVYAFVGDQRLVWKGRKEDYIQMSSGESLDPRVVEAVLDKCPAIARSCVVGNNFLKTSSQVVCAIVQPAKNT----------STTEITRAISVANRSLAPPLRISWSRVLVLSEGQEVPITKKGAIFRKKLEELFGAQLGALLSRPEV-------DIASRAKTK----PSASSSRAQGKTRDQIASIVSNIVLQTLRISEETMDDNSQATFAELGMDSAMSTLIVNKLNRQL-DMSLPLNTCHTHIDLVSLTNAILSDLGIDASSAKARPSTRVAPPAHEKEEIVIVGQAVRLPGDINTPDSFWRALIDKR-EDIITAVPASRWDHASFYRAPDSKEPPAPCDITLEKAGFVDSYSFDHAFFGISSAEAFHVSPNIRLSMEVAFEALENANIPPSKVKGSNMAVFVAASMDEGYIKLLFADKGWGAYTRFYGTGVATSTACGRLSYLLDVHGPSITIDTACSSGLIAFDQAVQYLQSGQ-GESAIVCGANTHAWPGTLGFLSAQKMTSSNSRCATFTNMADGYVPSEAAAGLIMKTKSAALRDGDRIIGVVRSTDVQHDGRSQGLVAPNVKAQIAMQIALLEKAQLSPAQIDFIEAHGTGTSLGDLIEIQGINEVFEGSHG-ADKPLVVGAAKSCVGHAELVAGLIGVVKTLGSFAKGSVPGLVQLTADNMNPNIDCSVVPLHIPIEPTVLK-TEDNLPLRALILSNGFAGSIAGTILEAPT-----EDMQPKASANIPETMPMTFVVSGKSQDALNEYLSLYLDFCLDADSSLFHAICYTTCIGREHYRYRFACVVNNMQDLIARLEDRLQNTSS--TSAGG-NARRILLGFPGQGSQYQGMGRYLANQYSGFRTIITEAANKAAGLTGYPILPYLLDESAPKGLTIDHSEVAQVCIFIFQYSVATWLESIGIHAHAVLGHSLGEIAAAVIARTFTLEIGLQFVVERAKLLRADPTRPAGMAALQTTEARVAQYIQKLGVEG--RVAIAVYNAPDAHVVSGELKAVESVLAAAKRDGVRCTKLNVDQGFHSPAVASALPSLKMWLDNHDDAITGLEKPFFSTLRGAEIPKHERLDTQYWIAHAKSPVRFYETARVATKASSIDVIVDVGPQPTVWSNMQTPEYAGKARLAFTGKRGKDQIVAMLAALSSLFE-KGFNVDFDALFSQM-P-YKFAMTDVPTYPFQRLYNYPAYICTRSSTVASILNQVET--SQQKAPTPQFVVDQSLCDFLDLHRIEGRRVLPGAAMVDFFARAAGSKSVKNVKFHTPLVLETPETQVRAEIDEQGAYKLVQDDGADTLICSGTISDKRGSSLGRK-VAHEPEAVPLQMMTKTQIYECFKNVQFGDPFRTVQAVRIWADYADADIRLEATAY--PAGDRIRKLDACLHMFGALSSRLAPPVDDNAGAYLPASLEDFTLHTDDMPYKFTCRYYLPLDIGRNARVLSSCFEVFSDAGDLLVSCKKYSVAWVPKGVVHKEQK-P----QQTAPDTWIRNAWTTQNLPAPQTTSVHRFDEIIYFGNGETSRVLSSLSSSAKNCISVEMPHLPRDESGKIPNN-------TKVVSP---EDMNKLPAILRGQDMLVVLDLSKSNNSPGSDQFTALYLQALMFLKHIMSHKFHISSFLALTSWSAPVDLYKEGLDLFSDSKVS-SASLVGAVVQGMIRVFRRETGLDFAAWCLDLTSIDSLTDSQLQNILTSEIQARYRSEFLDTFVCYREDADKKSLSRLVPSLESLERV--PARTPSGTTVIVGMGSIGTALAASLVEVGCNPVIFFGRRPDSQEKVVNELSALPENVRKQCQYRQVDVCDMEALKKALADVNATHGGIKNIIHTAAVVTDSTIVATKPSDFEAVLLPKVTGSWNLHVASQELNLALDSFVLFSSTNVIVGNPGQVSYVAANSFMDSLATFRHNCGLPGASLQLGAWESRLISDVNMENSFALLMKNDEGLPLILKAMMAPIPLQIIARMDTSKLSANPAYAKDPFFAPLLSSSNGAAPK-DTKAKLSKENAQKILIDILRVALELQPSEKLDTS------------------EELTALGADSITFAQFKGQVLKEFAVDVPMVYLSDGYTISDMINNVLESYG--VASL","order":8},{"start":1,"name":"hsPKS(Δ2)/1-1543","end":1543,"id":"1445639764","seq":"MAASLY--DKNTLLNAFLGVAHSAD-VDRNAVEYGNERWTYGDLDTVSTGLALEMHKKYGPKPVVAIVSENHPYTLAMLFAIWKLGGIAAPLDHNVPKDIMERMLLNIGPTCVLVPATERVVQSIVEGISVACHAFNPKEMSITALMQKYLDLSPELTGPAFHLPNPDDIALYLHTSSASSVANVKCVPTTHASILGASAARLAWWKRTWPAQQYTHLRVLGWSTWAHVIGLTNDLGAAMVLTAGCYIFAMPPASGAGGNA-----------AALYLDVCGQLLETAIIKQPTVFAGVPWVLEGFMRNYKQEAD------AARKQ---AIQDAVKRLKVFGSGGASTNAECIEWAIQMAIPLVLDIGMTEVGGPLFHSTIGG------PEGWLSEDCMLPGAQLKLIDDSG----AEV--STEGELVVRAQNVTRGYRHYDNSSFTLENDGTVSFKTGDVYAFVGDQRLVWKGRKEDYIQMSSGESLDPRVVEAVLDKCPAIARSCVVGNNFLKTSSQVVCAIVQPAKNT----------STTEITRAISVANRSLAPPLRISWSRVLVLSEGQEVPITKKGAIFRKKLEELFGAQLGALLSRPEV-------DIASRAKTK----PSASSSRAQGKTRDQIASIVSNIVLQTLRISEETMDDNSQATFAELGMDSAMSTLIVNKLNRQL-DMSLPLNTCHTHIDLVSLTNAILSDLGIDASSAKARPSTRVAPPAHEKEEIVIVGQAVRLPGDINTPDSFWRALIDKR-EDIITAVPASRWDHASFYRAPDSKEPPAPCDITLEKAGFVDSYSFDHAFFGISSAEAFHVSPNIRLSMEVAFEALENANIPPSKVKGSNMAVFVAASMDEGYIKLLFADKGWGAYTRFYGTGVATSTACGRLSYLLDVHGPSITIDTACSSGLIAFDQAVQYLQSGQ-GESAIVCGANTHAWPGTLGFLSAQKMTSSNSRCATFTNMADGYVPSEAAAGLIMKTKSAALRDGDRIIGVVRSTDVQHDGRSQGLVAPNVKAQIAMQIALLEKAQLSPAQIDFIEAHGTGTSLGDLIEIQGINEVFEGSHG-ADKPLVVGAAKSCVGHAELVAGLIGVVKTLGSFAKGSVPGLVQLTADNMNPNIDCSVVPLHIPIEPTVLK-TEDNLPLRALILSNGFAGSIAGTILEAPT-----EDMQPKASANIPETMPMTFVVSGKSQDALNEYLSLYLDFCLDADSSLFHAICYTTCIGREHYRYRFACVVNNMQDLIARLEDRLQNTSS--TSAGG-NARRILLGFPGQGSQYQGMGRYLANQYSGFRTIITEAANKAAGLTGYPILPYLLDESAPKGLTIDHSEVAQVCIFIFQYSVATWLESIGIHAHAVLGHSLGEIAAAVIARTFTLEIGLQFVVERAKLLRADPTRPAGMAALQTTEARVAQYIQKLGVEG--RVAIAVYNAPDAHVVSGELKAVESVLAAAKRDGVRCTKLNVDQGFHSPAVASALPSLKMWLDNHDDAITGLEKPFFSTLRGAEIPKHERLDTQYWIAHAKSPVRFYETARVATKASSIDVIVDVGPQPTVWSNMQTPEYAGKARLAFTGKRGKDQIVAMLAALSSLFE-KGS------------------------------------------------------------------------------------------------------------------------------------------------------------------------------------------------------------------------------------------------------------------------------------------------------------------------------------------------------------------------------------------------------------------------------------------------------------------------------------------------------------------------------------------------------------------------------------------------------------------------------------------------------------------------------------------------------------------------------------------------------------------------------------------------------------------------------------------------------------------------------------------------------------------------------------------------------------------------------------------------","order":9},{"start":1,"name":"hsPKS(Δ2&C)/1-1714","end":1714,"id":"1834786205","seq":"MAASLY--DKNTLLNAFLGVAHSAD-VDRNAVEYGNERWTYGDLDTVSTGLALEMHKKYGPKPVVAIVSENHPYTLAMLFAIWKLGGIAAPLDHNVPKDIMERMLLNIGPTCVLVPATERVVQSIVEGISVACHAFNPKEMSITALMQKYLDLSPELTGPAFHLPNPDDIALYLHTSSASSVANVKCVPTTHASILGASAARLAWWKRTWPAQQYTHLRVLGWSTWAHVIGLTNDLGAAMVLTAGCYIFAMPPASGAGGNA-----------AALYLDVCGQLLETAIIKQPTVFAGVPWVLEGFMRNYKQEAD------AARKQ---AIQDAVKRLKVFGSGGASTNAECIEWAIQMAIPLVLDIGMTEVGGPLFHSTIGG------PEGWLSEDCMLPGAQLKLIDDSG----AEV--STEGELVVRAQNVTRGYRHYDNSSFTLENDGTVSFKTGDVYAFVGDQRLVWKGRKEDYIQMSSGESLDPRVVEAVLDKCPAIARSCVVGNNFLKTSSQVVCAIVQPAKNT----------STTEITRAISVANRSLAPPLRISWSRVLVLSEGQEVPITKKGAIFRKKLEELFGAQLGALLSRPEV-------DIASRAKTK----PSASSSRAQGKTRDQIASIVSNIVLQTLRISEETMDDNSQATFAELGMDSAMSTLIVNKLNRQL-DMSLPLNTCHTHIDLVSLTNAILSDLGIDASSAKARPSTRVAPPAHEKEEIVIVGQAVRLPGDINTPDSFWRALIDKR-EDIITAVPASRWDHASFYRAPDSKEPPAPCDITLEKAGFVDSYSFDHAFFGISSAEAFHVSPNIRLSMEVAFEALENANIPPSKVKGSNMAVFVAASMDEGYIKLLFADKGWGAYTRFYGTGVATSTACGRLSYLLDVHGPSITIDTACSSGLIAFDQAVQYLQSGQ-GESAIVCGANTHAWPGTLGFLSAQKMTSSNSRCATFTNMADGYVPSEAAAGLIMKTKSAALRDGDRIIGVVRSTDVQHDGRSQGLVAPNVKAQIAMQIALLEKAQLSPAQIDFIEAHGTGTSLGDLIEIQGINEVFEGSHG-ADKPLVVGAAKSCVGHAELVAGLIGVVKTLGSFAKGSVPGLVQLTADNMNPNIDCSVVPLHIPIEPTVLK-TEDNLPLRALILSNGFAGSIAGTILEAPT-----EDMQPKASANIPETMPMTFVVSGKSQDALNEYLSLYLDFCLDADSSLFHAICYTTCIGREHYRYRFACVVNNMQDLIARLEDRLQNTSS--TSAGG-NARRILLGFPGQGSQYQGMGRYLANQYSGFRTIITEAANKAAGLTGYPILPYLLDESAPKGLTIDHSEVAQVCIFIFQYSVATWLESIGIHAHAVLGHSLGEIAAAVIARTFTLEIGLQFVVERAKLLRADPTRPAGMAALQTTEARVAQYIQKLGVEG--RVAIAVYNAPDAHVVSGELKAVESVLAAAKRDGVRCTKLNVDQGFHSPAVASALPSLKMWLDNHDDAITGLEKPFFSTLRGAEIPKHERLDTQYWIAHAKSPVRFYETARVATKASSIDVIVDVGPQPTVWSNMQTPEYAGKARLAFTGKRGKDQIVAMLAALSSLFE-KGSGGG--------------------------------------------------------------------------------------------------------------------------------------------------G-------------------------------------------------------------------------------------------------------------------------------------------------------------------------------------------------------------------------------------------------------------------------------------------------------------------------------------------------------------------------------------------------------------------------------------------------------------------SG---------------------------------------------------------------------------------------------------------------------------------------------------------------------------GGGSGGGGSDVNMENSFALLMKNDEGLPLILKAMMAPIPLQIIARMDTSKLSANPAYAKDPFFAPLLSSSNGAAPK-DTKAKLSKENAQKILIDILRVALELQPSEKLDTS------------------EELTALGADSITFAQFKGQVLKEFAVDVPMVYLSDGYTISDMINNVLESYG--VASL","order":10}],"appSettings":{"globalColorScheme":"% Identity","webStartUrl":"https://www.jalview.org/services/launchApp","application":"Jalview","showSeqFeatures":"false","version":"2.11.2.5"},"seqGroups":[{"displayText":true,"groupName":"JGroup:2095019979","sequenceRefs":["1630866817","2143416565","493519005"],"startRes":0,"colourText":false,"endRes":2574,"showNonconserved":false,"displayBoxes":true,"colourScheme":"% Identity"}],"alignAnnotation":[{"annotationSettings":{"scaleColLabel":false,"visible":true,"hasIcon":true,"showAllColLabels":false,"belowAlignment":true,"centreColLabels":false},"score":0,"graphType":0,"calcId":"","annotations":[{"value":0,"secondaryStructure":"\u0000"},{"value":0,"secondaryStructure":"\u0000"},{"value":0,"secondaryStructure":"\u0000"},{"value":0,"secondaryStructure":"\u0000"},{"value":0,"secondaryStructure":"\u0000"},{"value":0,"secondaryStructure":"\u0000"},{"value":0,"secondaryStructure":"\u0000"},{"value":0,"secondaryStructure":"\u0000"},{"value":0,"secondaryStructure":"\u0000"},{"value":0,"secondaryStructure":"\u0000"},{"value":0,"secondaryStructure":"\u0000"},{"value":0,"secondaryStructure":"\u0000"},{"value":0,"secondaryStructure":"\u0000"},{"value":0,"secondaryStructure":"\u0000"},{"value":0,"secondaryStructure":"\u0000"},{"value":0,"secondaryStructure":"\u0000"},{"value":0,"secondaryStructure":"\u0000"},{"value":0,"secondaryStructure":"\u0000"},{"value":0,"secondaryStructure":"\u0000"},{"value":0,"secondaryStructure":"\u0000"},{"value":0,"secondaryStructure":"\u0000"},{"value":0,"secondaryStructure":"\u0000"},{"value":0,"secondaryStructure":"\u0000"},{"value":0,"secondaryStructure":"\u0000"},{"value":0,"secondaryStructure":"\u0000"},{"value":0,"secondaryStructure":"\u0000"},{"description":"","displayCharacter":"AMP-binding domain","value":0,"secondaryStructure":"H"},{"description":"","displayCharacter":"AMP-binding domain","value":0,"secondaryStructure":"H"},{"description":"","displayCharacter":"AMP-binding domain","value":0,"secondaryStructure":"H"},{"description":"","displayCharacter":"AMP-binding domain","value":0,"secondaryStructure":"H"},{"description":"","displayCharacter":"AMP-binding domain","value":0,"secondaryStructure":"H"},{"description":"","displayCharacter":"AMP-binding domain","value":0,"secondaryStructure":"H"},{"description":"","displayCharacter":"AMP-binding domain","value":0,"secondaryStructure":"H"},{"description":"","displayCharacter":"AMP-binding domain","value":0,"secondaryStructure":"H"},{"description":"","displayCharacter":"AMP-binding domain","value":0,"secondaryStructure":"H"},{"description":"","displayCharacter":"AMP-binding domain","value":0,"secondaryStructure":"H"},{"description":"","displayCharacter":"AMP-binding domain","value":0,"secondaryStructure":"H"},{"description":"","displayCharacter":"AMP-binding domain","value":0,"secondaryStructure":"H"},{"description":"","displayCharacter":"AMP-binding domain","value":0,"secondaryStructure":"H"},{"description":"","displayCharacter":"AMP-binding domain","value":0,"secondaryStructure":"H"},{"description":"","displayCharacter":"AMP-binding domain","value":0,"secondaryStructure":"H"},{"description":"","displayCharacter":"AMP-binding domain","value":0,"secondaryStructure":"H"},{"description":"","displayCharacter":"AMP-binding domain","value":0,"secondaryStructure":"H"},{"description":"","displayCharacter":"AMP-binding domain","value":0,"secondaryStructure":"H"},{"description":"","displayCharacter":"AMP-binding domain","value":0,"secondaryStructure":"H"},{"description":"","displayCharacter":"AMP-binding domain","value":0,"secondaryStructure":"H"},{"description":"","displayCharacter":"AMP-binding domain","value":0,"secondaryStructure":"H"},{"description":"","displayCharacter":"AMP-binding domain","value":0,"secondaryStructure":"H"},{"description":"","displayCharacter":"AMP-binding domain","value":0,"secondaryStructure":"H"},{"description":"","displayCharacter":"AMP-binding domain","value":0,"secondaryStructure":"H"},{"description":"","displayCharacter":"AMP-binding domain","value":0,"secondaryStructure":"H"},{"description":"","displayCharacter":"AMP-binding domain","value":0,"secondaryStructure":"H"},{"description":"","displayCharacter":"AMP-binding domain","value":0,"secondaryStructure":"H"},{"description":"","displayCharacter":"AMP-binding domain","value":0,"secondaryStructure":"H"},{"description":"","displayCharacter":"AMP-binding domain","value":0,"secondaryStructure":"H"},{"description":"","displayCharacter":"AMP-binding domain","value":0,"secondaryStructure":"H"},{"description":"","displayCharacter":"AMP-binding domain","value":0,"secondaryStructure":"H"},{"description":"","displayCharacter":"AMP-binding domain","value":0,"secondaryStructure":"H"},{"description":"","displayCharacter":"AMP-binding domain","value":0,"secondaryStructure":"H"},{"description":"","displayCharacter":"AMP-binding domain","value":0,"secondaryStructure":"H"},{"description":"","displayCharacter":"AMP-binding domain","value":0,"secondaryStructure":"H"},{"description":"","displayCharacter":"AMP-binding domain","value":0,"secondaryStructure":"H"},{"description":"","displayCharacter":"AMP-binding domain","value":0,"secondaryStructure":"H"},{"description":"","displayCharacter":"AMP-binding domain","value":0,"secondaryStructure":"H"},{"description":"","displayCharacter":"AMP-binding domain","value":0,"secondaryStructure":"H"},{"description":"","displayCharacter":"AMP-binding domain","value":0,"secondaryStructure":"H"},{"description":"","displayCharacter":"AMP-binding domain","value":0,"secondaryStructure":"H"},{"description":"","displayCharacter":"AMP-binding domain","value":0,"secondaryStructure":"H"},{"description":"","displayCharacter":"AMP-binding domain","value":0,"secondaryStructure":"H"},{"description":"","displayCharacter":"AMP-binding domain","value":0,"secondaryStructure":"H"},{"description":"","displayCharacter":"AMP-binding domain","value":0,"secondaryStructure":"H"},{"description":"","displayCharacter":"AMP-binding domain","value":0,"secondaryStructure":"H"},{"description":"","displayCharacter":"AMP-binding domain","value":0,"secondaryStructure":"H"},{"description":"","displayCharacter":"AMP-binding domain","value":0,"secondaryStructure":"H"},{"description":"","displayCharacter":"AMP-binding domain","value":0,"secondaryStructure":"H"},{"description":"","displayCharacter":"AMP-binding domain","value":0,"secondaryStructure":"H"},{"description":"","displayCharacter":"AMP-binding domain","value":0,"secondaryStructure":"H"},{"description":"","displayCharacter":"AMP-binding domain","value":0,"secondaryStructure":"H"},{"description":"","displayCharacter":"AMP-binding domain","value":0,"secondaryStructure":"H"},{"description":"","displayCharacter":"AMP-binding domain","value":0,"secondaryStructure":"H"},{"description":"","displayCharacter":"AMP-binding domain","value":0,"secondaryStructure":"H"},{"description":"","displayCharacter":"AMP-binding domain","value":0,"secondaryStructure":"H"},{"description":"","displayCharacter":"AMP-binding domain","value":0,"secondaryStructure":"H"},{"description":"","displayCharacter":"AMP-binding domain","value":0,"secondaryStructure":"H"},{"description":"","displayCharacter":"AMP-binding domain","value":0,"secondaryStructure":"H"},{"description":"","displayCharacter":"AMP-binding domain","value":0,"secondaryStructure":"H"},{"description":"","displayCharacter":"AMP-binding domain","value":0,"secondaryStructure":"H"},{"description":"","displayCharacter":"AMP-binding domain","value":0,"secondaryStructure":"H"},{"description":"","displayCharacter":"AMP-binding domain","value":0,"secondaryStructure":"H"},{"description":"","displayCharacter":"AMP-binding domain","value":0,"secondaryStructure":"H"},{"description":"","displayCharacter":"AMP-binding domain","value":0,"secondaryStructure":"H"},{"description":"","displayCharacter":"AMP-binding domain","value":0,"secondaryStructure":"H"},{"description":"","displayCharacter":"AMP-binding domain","value":0,"secondaryStructure":"H"},{"description":"","displayCharacter":"AMP-binding domain","value":0,"secondaryStructure":"H"},{"description":"","displayCharacter":"AMP-binding domain","value":0,"secondaryStructure":"H"},{"description":"","displayCharacter":"AMP-binding domain","value":0,"secondaryStructure":"H"},{"description":"","displayCharacter":"AMP-binding domain","value":0,"secondaryStructure":"H"},{"description":"","displayCharacter":"AMP-binding domain","value":0,"secondaryStructure":"H"},{"description":"","displayCharacter":"AMP-binding domain","value":0,"secondaryStructure":"H"},{"description":"","displayCharacter":"AMP-binding domain","value":0,"secondaryStructure":"H"},{"description":"","displayCharacter":"AMP-binding domain","value":0,"secondaryStructure":"H"},{"description":"","displayCharacter":"AMP-binding domain","value":0,"secondaryStructure":"H"},{"description":"","displayCharacter":"AMP-binding domain","value":0,"secondaryStructure":"H"},{"description":"","displayCharacter":"AMP-binding domain","value":0,"secondaryStructure":"H"},{"description":"","displayCharacter":"AMP-binding domain","value":0,"secondaryStructure":"H"},{"description":"","displayCharacter":"AMP-binding domain","value":0,"secondaryStructure":"H"},{"description":"","displayCharacter":"AMP-binding domain","value":0,"secondaryStructure":"H"},{"description":"","displayCharacter":"AMP-binding domain","value":0,"secondaryStructure":"H"},{"description":"","displayCharacter":"AMP-binding domain","value":0,"secondaryStructure":"H"},{"description":"","displayCharacter":"AMP-binding domain","value":0,"secondaryStructure":"H"},{"description":"","displayCharacter":"AMP-binding domain","value":0,"secondaryStructure":"H"},{"description":"","displayCharacter":"AMP-binding domain","value":0,"secondaryStructure":"H"},{"description":"","displayCharacter":"AMP-binding domain","value":0,"secondaryStructure":"H"},{"description":"","displayCharacter":"AMP-binding domain","value":0,"secondaryStructure":"H"},{"description":"","displayCharacter":"AMP-binding domain","value":0,"secondaryStructure":"H"},{"description":"","displayCharacter":"AMP-binding domain","value":0,"secondaryStructure":"H"},{"description":"","displayCharacter":"AMP-binding domain","value":0,"secondaryStructure":"H"},{"description":"","displayCharacter":"AMP-binding domain","value":0,"secondaryStructure":"H"},{"description":"","displayCharacter":"AMP-binding domain","value":0,"secondaryStructure":"H"},{"description":"","displayCharacter":"AMP-binding domain","value":0,"secondaryStructure":"H"},{"description":"","displayCharacter":"AMP-binding domain","value":0,"secondaryStructure":"H"},{"description":"","displayCharacter":"AMP-binding domain","value":0,"secondaryStructure":"H"},{"description":"","displayCharacter":"AMP-binding domain","value":0,"secondaryStructure":"H"},{"description":"","displayCharacter":"AMP-binding domain","value":0,"secondaryStructure":"H"},{"description":"","displayCharacter":"AMP-binding domain","value":0,"secondaryStructure":"H"},{"description":"","displayCharacter":"AMP-binding domain","value":0,"secondaryStructure":"H"},{"description":"","displayCharacter":"AMP-binding domain","value":0,"secondaryStructure":"H"},{"description":"","displayCharacter":"AMP-binding domain","value":0,"secondaryStructure":"H"},{"description":"","displayCharacter":"AMP-binding domain","value":0,"secondaryStructure":"H"},{"description":"","displayCharacter":"AMP-binding domain","value":0,"secondaryStructure":"H"},{"description":"","displayCharacter":"AMP-binding domain","value":0,"secondaryStructure":"H"},{"description":"","displayCharacter":"AMP-binding domain","value":0,"secondaryStructure":"H"},{"description":"","displayCharacter":"AMP-binding domain","value":0,"secondaryStructure":"H"},{"description":"","displayCharacter":"AMP-binding domain","value":0,"secondaryStructure":"H"},{"description":"","displayCharacter":"AMP-binding domain","value":0,"secondaryStructure":"H"},{"description":"","displayCharacter":"AMP-binding domain","value":0,"secondaryStructure":"H"},{"description":"","displayCharacter":"AMP-binding domain","value":0,"secondaryStructure":"H"},{"description":"","displayCharacter":"AMP-binding domain","value":0,"secondaryStructure":"H"},{"description":"","displayCharacter":"AMP-binding domain","value":0,"secondaryStructure":"H"},{"description":"","displayCharacter":"AMP-binding domain","value":0,"secondaryStructure":"H"},{"description":"","displayCharacter":"AMP-binding domain","value":0,"secondaryStructure":"H"},{"description":"","displayCharacter":"AMP-binding domain","value":0,"secondaryStructure":"H"},{"description":"","displayCharacter":"AMP-binding domain","value":0,"secondaryStructure":"H"},{"description":"","displayCharacter":"AMP-binding domain","value":0,"secondaryStructure":"H"},{"description":"","displayCharacter":"AMP-binding domain","value":0,"secondaryStructure":"H"},{"description":"","displayCharacter":"AMP-binding domain","value":0,"secondaryStructure":"H"},{"description":"","displayCharacter":"AMP-binding domain","value":0,"secondaryStructure":"H"},{"description":"","displayCharacter":"AMP-binding domain","value":0,"secondaryStructure":"H"},{"description":"","displayCharacter":"AMP-binding domain","value":0,"secondaryStructure":"H"},{"description":"","displayCharacter":"AMP-binding domain","value":0,"secondaryStructure":"H"},{"description":"","displayCharacter":"AMP-binding domain","value":0,"secondaryStructure":"H"},{"description":"","displayCharacter":"AMP-binding domain","value":0,"secondaryStructure":"H"},{"description":"","displayCharacter":"AMP-binding domain","value":0,"secondaryStructure":"H"},{"description":"","displayCharacter":"AMP-binding domain","value":0,"secondaryStructure":"H"},{"description":"","displayCharacter":"AMP-binding domain","value":0,"secondaryStructure":"H"},{"description":"","displayCharacter":"AMP-binding domain","value":0,"secondaryStructure":"H"},{"description":"","displayCharacter":"AMP-binding domain","value":0,"secondaryStructure":"H"},{"description":"","displayCharacter":"AMP-binding domain","value":0,"secondaryStructure":"H"},{"description":"","displayCharacter":"AMP-binding domain","value":0,"secondaryStructure":"H"},{"description":"","displayCharacter":"AMP-binding domain","value":0,"secondaryStructure":"H"},{"description":"","displayCharacter":"AMP-binding domain","value":0,"secondaryStructure":"H"},{"description":"","displayCharacter":"AMP-binding domain","value":0,"secondaryStructure":"H"},{"description":"","displayCharacter":"AMP-binding domain","value":0,"secondaryStructure":"H"},{"description":"","displayCharacter":"AMP-binding domain","value":0,"secondaryStructure":"H"},{"description":"","displayCharacter":"AMP-binding domain","value":0,"secondaryStructure":"H"},{"description":"","displayCharacter":"AMP-binding domain","value":0,"secondaryStructure":"H"},{"description":"","displayCharacter":"AMP-binding domain","value":0,"secondaryStructure":"H"},{"description":"","displayCharacter":"AMP-binding domain","value":0,"secondaryStructure":"H"},{"description":"","displayCharacter":"AMP-binding domain","value":0,"secondaryStructure":"H"},{"description":"","displayCharacter":"AMP-binding domain","value":0,"secondaryStructure":"H"},{"description":"","displayCharacter":"AMP-binding domain","value":0,"secondaryStructure":"H"},{"description":"","displayCharacter":"AMP-binding domain","value":0,"secondaryStructure":"H"},{"description":"","displayCharacter":"AMP-binding domain","value":0,"secondaryStructure":"H"},{"description":"","displayCharacter":"AMP-binding domain","value":0,"secondaryStructure":"H"},{"description":"","displayCharacter":"AMP-binding domain","value":0,"secondaryStructure":"H"},{"description":"","displayCharacter":"AMP-binding domain","value":0,"secondaryStructure":"H"},{"description":"","displayCharacter":"AMP-binding domain","value":0,"secondaryStructure":"H"},{"description":"","displayCharacter":"AMP-binding domain","value":0,"secondaryStructure":"H"},{"description":"","displayCharacter":"AMP-binding domain","value":0,"secondaryStructure":"H"},{"description":"","displayCharacter":"AMP-binding domain","value":0,"secondaryStructure":"H"},{"description":"","displayCharacter":"AMP-binding domain","value":0,"secondaryStructure":"H"},{"description":"","displayCharacter":"AMP-binding domain","value":0,"secondaryStructure":"H"},{"description":"","displayCharacter":"AMP-binding domain","value":0,"secondaryStructure":"H"},{"description":"","displayCharacter":"AMP-binding domain","value":0,"secondaryStructure":"H"},{"description":"","displayCharacter":"AMP-binding domain","value":0,"secondaryStructure":"H"},{"description":"","displayCharacter":"AMP-binding domain","value":0,"secondaryStructure":"H"},{"description":"","displayCharacter":"AMP-binding domain","value":0,"secondaryStructure":"H"},{"description":"","displayCharacter":"AMP-binding domain","value":0,"secondaryStructure":"H"},{"description":"","displayCharacter":"AMP-binding domain","value":0,"secondaryStructure":"H"},{"description":"","displayCharacter":"AMP-binding domain","value":0,"secondaryStructure":"H"},{"description":"","displayCharacter":"AMP-binding domain","value":0,"secondaryStructure":"H"},{"description":"","displayCharacter":"AMP-binding domain","value":0,"secondaryStructure":"H"},{"description":"","displayCharacter":"AMP-binding domain","value":0,"secondaryStructure":"H"},{"description":"","displayCharacter":"AMP-binding domain","value":0,"secondaryStructure":"H"},{"description":"","displayCharacter":"AMP-binding domain","value":0,"secondaryStructure":"H"},{"description":"","displayCharacter":"AMP-binding domain","value":0,"secondaryStructure":"H"},{"description":"","displayCharacter":"AMP-binding domain","value":0,"secondaryStructure":"H"},{"description":"","displayCharacter":"AMP-binding domain","value":0,"secondaryStructure":"H"},{"description":"","displayCharacter":"AMP-binding domain","value":0,"secondaryStructure":"H"},{"description":"","displayCharacter":"AMP-binding domain","value":0,"secondaryStructure":"H"},{"description":"","displayCharacter":"AMP-binding domain","value":0,"secondaryStructure":"H"},{"description":"","displayCharacter":"AMP-binding domain","value":0,"secondaryStructure":"H"},{"description":"","displayCharacter":"AMP-binding domain","value":0,"secondaryStructure":"H"},{"description":"","displayCharacter":"AMP-binding domain","value":0,"secondaryStructure":"H"},{"description":"","displayCharacter":"AMP-binding domain","value":0,"secondaryStructure":"H"},{"description":"","displayCharacter":"AMP-binding domain","value":0,"secondaryStructure":"H"},{"description":"","displayCharacter":"AMP-binding domain","value":0,"secondaryStructure":"H"},{"description":"","displayCharacter":"AMP-binding domain","value":0,"secondaryStructure":"H"},{"description":"","displayCharacter":"AMP-binding domain","value":0,"secondaryStructure":"H"},{"description":"","displayCharacter":"AMP-binding domain","value":0,"secondaryStructure":"H"},{"description":"","displayCharacter":"AMP-binding domain","value":0,"secondaryStructure":"H"},{"description":"","displayCharacter":"AMP-binding domain","value":0,"secondaryStructure":"H"},{"description":"","displayCharacter":"AMP-binding domain","value":0,"secondaryStructure":"H"},{"description":"","displayCharacter":"AMP-binding domain","value":0,"secondaryStructure":"H"},{"description":"","displayCharacter":"AMP-binding domain","value":0,"secondaryStructure":"H"},{"description":"","displayCharacter":"AMP-binding domain","value":0,"secondaryStructure":"H"},{"description":"","displayCharacter":"AMP-binding domain","value":0,"secondaryStructure":"H"},{"description":"","displayCharacter":"AMP-binding domain","value":0,"secondaryStructure":"H"},{"description":"","displayCharacter":"AMP-binding domain","value":0,"secondaryStructure":"H"},{"description":"","displayCharacter":"AMP-binding domain","value":0,"secondaryStructure":"H"},{"description":"","displayCharacter":"AMP-binding domain","value":0,"secondaryStructure":"H"},{"description":"","displayCharacter":"AMP-binding domain","value":0,"secondaryStructure":"H"},{"description":"","displayCharacter":"AMP-binding domain","value":0,"secondaryStructure":"H"},{"description":"","displayCharacter":"AMP-binding domain","value":0,"secondaryStructure":"H"},{"description":"","displayCharacter":"AMP-binding domain","value":0,"secondaryStructure":"H"},{"description":"","displayCharacter":"AMP-binding domain","value":0,"secondaryStructure":"H"},{"description":"","displayCharacter":"AMP-binding domain","value":0,"secondaryStructure":"H"},{"description":"","displayCharacter":"AMP-binding domain","value":0,"secondaryStructure":"H"},{"description":"","displayCharacter":"AMP-binding domain","value":0,"secondaryStructure":"H"},{"description":"","displayCharacter":"AMP-binding domain","value":0,"secondaryStructure":"H"},{"description":"","displayCharacter":"AMP-binding domain","value":0,"secondaryStructure":"H"},{"description":"","displayCharacter":"AMP-binding domain","value":0,"secondaryStructure":"H"},{"description":"","displayCharacter":"AMP-binding domain","value":0,"secondaryStructure":"H"},{"description":"","displayCharacter":"AMP-binding domain","value":0,"secondaryStructure":"H"},{"description":"","displayCharacter":"AMP-binding domain","value":0,"secondaryStructure":"H"},{"description":"","displayCharacter":"AMP-binding domain","value":0,"secondaryStructure":"H"},{"description":"","displayCharacter":"AMP-binding domain","value":0,"secondaryStructure":"H"},{"description":"","displayCharacter":"AMP-binding domain","value":0,"secondaryStructure":"H"},{"description":"","displayCharacter":"AMP-binding domain","value":0,"secondaryStructure":"H"},{"description":"","displayCharacter":"AMP-binding domain","value":0,"secondaryStructure":"H"},{"description":"","displayCharacter":"AMP-binding domain","value":0,"secondaryStructure":"H"},{"description":"","displayCharacter":"AMP-binding domain","value":0,"secondaryStructure":"H"},{"description":"","displayCharacter":"AMP-binding domain","value":0,"secondaryStructure":"H"},{"description":"","displayCharacter":"AMP-binding domain","value":0,"secondaryStructure":"H"},{"description":"","displayCharacter":"AMP-binding domain","value":0,"secondaryStructure":"H"},{"description":"","displayCharacter":"AMP-binding domain","value":0,"secondaryStructure":"H"},{"description":"","displayCharacter":"AMP-binding domain","value":0,"secondaryStructure":"H"},{"description":"","displayCharacter":"AMP-binding domain","value":0,"secondaryStructure":"H"},{"description":"","displayCharacter":"AMP-binding domain","value":0,"secondaryStructure":"H"},{"description":"","displayCharacter":"AMP-binding domain","value":0,"secondaryStructure":"H"},{"description":"","displayCharacter":"AMP-binding domain","value":0,"secondaryStructure":"H"},{"description":"","displayCharacter":"AMP-binding domain","value":0,"secondaryStructure":"H"},{"description":"","displayCharacter":"AMP-binding domain","value":0,"secondaryStructure":"H"},{"description":"","displayCharacter":"AMP-binding domain","value":0,"secondaryStructure":"H"},{"description":"","displayCharacter":"AMP-binding domain","value":0,"secondaryStructure":"H"},{"description":"","displayCharacter":"AMP-binding domain","value":0,"secondaryStructure":"H"},{"description":"","displayCharacter":"AMP-binding domain","value":0,"secondaryStructure":"H"},{"description":"","displayCharacter":"AMP-binding domain","value":0,"secondaryStructure":"H"},{"description":"","displayCharacter":"AMP-binding domain","value":0,"secondaryStructure":"H"},{"description":"","displayCharacter":"AMP-binding domain","value":0,"secondaryStructure":"H"},{"description":"","displayCharacter":"AMP-binding domain","value":0,"secondaryStructure":"H"},{"description":"","displayCharacter":"AMP-binding domain","value":0,"secondaryStructure":"H"},{"description":"","displayCharacter":"AMP-binding domain","value":0,"secondaryStructure":"H"},{"description":"","displayCharacter":"AMP-binding domain","value":0,"secondaryStructure":"H"},{"description":"","displayCharacter":"AMP-binding domain","value":0,"secondaryStructure":"H"},{"description":"","displayCharacter":"AMP-binding domain","value":0,"secondaryStructure":"H"},{"description":"","displayCharacter":"AMP-binding domain","value":0,"secondaryStructure":"H"},{"description":"","displayCharacter":"AMP-binding domain","value":0,"secondaryStructure":"H"},{"description":"","displayCharacter":"AMP-binding domain","value":0,"secondaryStructure":"H"},{"description":"","displayCharacter":"AMP-binding domain","value":0,"secondaryStructure":"H"},{"description":"","displayCharacter":"AMP-binding domain","value":0,"secondaryStructure":"H"},{"description":"","displayCharacter":"AMP-binding domain","value":0,"secondaryStructure":"H"},{"description":"","displayCharacter":"AMP-binding domain","value":0,"secondaryStructure":"H"},{"description":"","displayCharacter":"AMP-binding domain","value":0,"secondaryStructure":"H"},{"description":"","displayCharacter":"AMP-binding domain","value":0,"secondaryStructure":"H"},{"description":"","displayCharacter":"AMP-binding domain","value":0,"secondaryStructure":"H"},{"description":"","displayCharacter":"AMP-binding domain","value":0,"secondaryStructure":"H"},{"description":"","displayCharacter":"AMP-binding domain","value":0,"secondaryStructure":"H"},{"description":"","displayCharacter":"AMP-binding domain","value":0,"secondaryStructure":"H"},{"description":"","displayCharacter":"AMP-binding domain","value":0,"secondaryStructure":"H"},{"description":"","displayCharacter":"AMP-binding domain","value":0,"secondaryStructure":"H"},{"description":"","displayCharacter":"AMP-binding domain","value":0,"secondaryStructure":"H"},{"description":"","displayCharacter":"AMP-binding domain","value":0,"secondaryStructure":"H"},{"description":"","displayCharacter":"AMP-binding domain","value":0,"secondaryStructure":"H"},{"description":"","displayCharacter":"AMP-binding domain","value":0,"secondaryStructure":"H"},{"description":"","displayCharacter":"AMP-binding domain","value":0,"secondaryStructure":"H"},{"description":"","displayCharacter":"AMP-binding domain","value":0,"secondaryStructure":"H"},{"description":"","displayCharacter":"AMP-binding domain","value":0,"secondaryStructure":"H"},{"description":"","displayCharacter":"AMP-binding domain","value":0,"secondaryStructure":"H"},{"description":"","displayCharacter":"AMP-binding domain","value":0,"secondaryStructure":"H"},{"description":"","displayCharacter":"AMP-binding domain","value":0,"secondaryStructure":"H"},{"description":"","displayCharacter":"AMP-binding domain","value":0,"secondaryStructure":"H"},{"description":"","displayCharacter":"AMP-binding domain","value":0,"secondaryStructure":"H"},{"description":"","displayCharacter":"AMP-binding domain","value":0,"secondaryStructure":"H"},{"description":"","displayCharacter":"AMP-binding domain","value":0,"secondaryStructure":"H"},{"description":"","displayCharacter":"AMP-binding domain","value":0,"secondaryStructure":"H"},{"description":"","displayCharacter":"AMP-binding domain","value":0,"secondaryStructure":"H"},{"description":"","displayCharacter":"AMP-binding domain","value":0,"secondaryStructure":"H"},{"description":"","displayCharacter":"AMP-binding domain","value":0,"secondaryStructure":"H"},{"description":"","displayCharacter":"AMP-binding domain","value":0,"secondaryStructure":"H"},{"description":"","displayCharacter":"AMP-binding domain","value":0,"secondaryStructure":"H"},{"description":"","displayCharacter":"AMP-binding domain","value":0,"secondaryStructure":"H"},{"description":"","displayCharacter":"AMP-binding domain","value":0,"secondaryStructure":"H"},{"description":"","displayCharacter":"AMP-binding domain","value":0,"secondaryStructure":"H"},{"description":"","displayCharacter":"AMP-binding domain","value":0,"secondaryStructure":"H"},{"description":"","displayCharacter":"AMP-binding domain","value":0,"secondaryStructure":"H"},{"description":"","displayCharacter":"AMP-binding domain","value":0,"secondaryStructure":"H"},{"description":"","displayCharacter":"AMP-binding domain","value":0,"secondaryStructure":"H"},{"description":"","displayCharacter":"AMP-binding domain","value":0,"secondaryStructure":"H"},{"description":"","displayCharacter":"AMP-binding domain","value":0,"secondaryStructure":"H"},{"description":"","displayCharacter":"AMP-binding domain","value":0,"secondaryStructure":"H"},{"description":"","displayCharacter":"AMP-binding domain","value":0,"secondaryStructure":"H"},{"description":"","displayCharacter":"AMP-binding domain","value":0,"secondaryStructure":"H"},{"description":"","displayCharacter":"AMP-binding domain","value":0,"secondaryStructure":"H"},{"description":"","displayCharacter":"AMP-binding domain","value":0,"secondaryStructure":"H"},{"description":"","displayCharacter":"AMP-binding domain","value":0,"secondaryStructure":"H"},{"description":"","displayCharacter":"AMP-binding domain","value":0,"secondaryStructure":"H"},{"description":"","displayCharacter":"AMP-binding domain","value":0,"secondaryStructure":"H"},{"description":"","displayCharacter":"AMP-binding domain","value":0,"secondaryStructure":"H"},{"description":"","displayCharacter":"AMP-binding domain","value":0,"secondaryStructure":"H"},{"description":"","displayCharacter":"AMP-binding domain","value":0,"secondaryStructure":"H"},{"description":"","displayCharacter":"AMP-binding domain","value":0,"secondaryStructure":"H"},{"description":"","displayCharacter":"AMP-binding domain","value":0,"secondaryStructure":"H"},{"description":"","displayCharacter":"AMP-binding domain","value":0,"secondaryStructure":"H"},{"description":"","displayCharacter":"AMP-binding domain","value":0,"secondaryStructure":"H"},{"description":"","displayCharacter":"AMP-binding domain","value":0,"secondaryStructure":"H"},{"description":"","displayCharacter":"AMP-binding domain","value":0,"secondaryStructure":"H"},{"description":"","displayCharacter":"AMP-binding domain","value":0,"secondaryStructure":"H"},{"description":"","displayCharacter":"AMP-binding domain","value":0,"secondaryStructure":"H"},{"description":"","displayCharacter":"AMP-binding domain","value":0,"secondaryStructure":"H"},{"description":"","displayCharacter":"AMP-binding domain","value":0,"secondaryStructure":"H"},{"description":"","displayCharacter":"AMP-binding domain","value":0,"secondaryStructure":"H"},{"description":"","displayCharacter":"AMP-binding domain","value":0,"secondaryStructure":"H"},{"description":"","displayCharacter":"AMP-binding domain","value":0,"secondaryStructure":"H"},{"description":"","displayCharacter":"AMP-binding domain","value":0,"secondaryStructure":"H"},{"description":"","displayCharacter":"AMP-binding domain","value":0,"secondaryStructure":"H"},{"description":"","displayCharacter":"AMP-binding domain","value":0,"secondaryStructure":"H"},{"description":"","displayCharacter":"AMP-binding domain","value":0,"secondaryStructure":"H"},{"description":"","displayCharacter":"AMP-binding domain","value":0,"secondaryStructure":"H"},{"description":"","displayCharacter":"AMP-binding domain","value":0,"secondaryStructure":"H"},{"description":"","displayCharacter":"AMP-binding domain","value":0,"secondaryStructure":"H"},{"description":"","displayCharacter":"AMP-binding domain","value":0,"secondaryStructure":"H"},{"description":"","displayCharacter":"AMP-binding domain","value":0,"secondaryStructure":"H"},{"description":"","displayCharacter":"AMP-binding domain","value":0,"secondaryStructure":"H"},{"description":"","displayCharacter":"AMP-binding domain","value":0,"secondaryStructure":"H"},{"description":"","displayCharacter":"AMP-binding domain","value":0,"secondaryStructure":"H"},{"description":"","displayCharacter":"AMP-binding domain","value":0,"secondaryStructure":"H"},{"description":"","displayCharacter":"AMP-binding domain","value":0,"secondaryStructure":"H"},{"description":"","displayCharacter":"AMP-binding domain","value":0,"secondaryStructure":"H"},{"description":"","displayCharacter":"AMP-binding domain","value":0,"secondaryStructure":"H"},{"description":"","displayCharacter":"AMP-binding domain","value":0,"secondaryStructure":"H"},{"description":"","displayCharacter":"AMP-binding domain","value":0,"secondaryStructure":"H"},{"description":"","displayCharacter":"AMP-binding domain","value":0,"secondaryStructure":"H"},{"description":"","displayCharacter":"AMP-binding domain","value":0,"secondaryStructure":"H"},{"description":"","displayCharacter":"AMP-binding domain","value":0,"secondaryStructure":"H"},{"description":"","displayCharacter":"AMP-binding domain","value":0,"secondaryStructure":"H"},{"description":"","displayCharacter":"AMP-binding domain","value":0,"secondaryStructure":"H"},{"description":"","displayCharacter":"AMP-binding domain","value":0,"secondaryStructure":"H"},{"description":"","displayCharacter":"AMP-binding domain","value":0,"secondaryStructure":"H"},{"description":"","displayCharacter":"AMP-binding domain","value":0,"secondaryStructure":"H"},{"description":"","displayCharacter":"AMP-binding domain","value":0,"secondaryStructure":"H"},{"description":"","displayCharacter":"AMP-binding domain","value":0,"secondaryStructure":"H"},{"description":"","displayCharacter":"AMP-binding domain","value":0,"secondaryStructure":"H"},{"description":"","displayCharacter":"AMP-binding domain","value":0,"secondaryStructure":"H"},{"description":"","displayCharacter":"AMP-binding domain","value":0,"secondaryStructure":"H"},{"description":"","displayCharacter":"AMP-binding domain","value":0,"secondaryStructure":"H"},{"description":"","displayCharacter":"AMP-binding domain","value":0,"secondaryStructure":"H"},{"description":"","displayCharacter":"AMP-binding domain","value":0,"secondaryStructure":"H"},{"description":"","displayCharacter":"AMP-binding domain","value":0,"secondaryStructure":"H"},{"description":"","displayCharacter":"AMP-binding domain","value":0,"secondaryStructure":"H"},{"description":"","displayCharacter":"AMP-binding domain","value":0,"secondaryStructure":"H"},{"description":"","displayCharacter":"AMP-binding domain","value":0,"secondaryStructure":"H"},{"description":"","displayCharacter":"AMP-binding domain","value":0,"secondaryStructure":"H"},{"description":"","displayCharacter":"AMP-binding domain","value":0,"secondaryStructure":"H"},{"description":"","displayCharacter":"AMP-binding domain","value":0,"secondaryStructure":"H"},{"description":"","displayCharacter":"AMP-binding domain","value":0,"secondaryStructure":"H"},{"description":"","displayCharacter":"AMP-binding domain","value":0,"secondaryStructure":"H"},{"description":"","displayCharacter":"AMP-binding domain","value":0,"secondaryStructure":"H"},{"description":"","displayCharacter":"AMP-binding domain","value":0,"secondaryStructure":"H"},{"description":"","displayCharacter":"AMP-binding domain","value":0,"secondaryStructure":"H"},{"description":"","displayCharacter":"AMP-binding domain","value":0,"secondaryStructure":"H"},{"description":"","displayCharacter":"AMP-binding domain","value":0,"secondaryStructure":"H"},{"description":"","displayCharacter":"AMP-binding domain","value":0,"secondaryStructure":"H"},{"description":"","displayCharacter":"AMP-binding domain","value":0,"secondaryStructure":"H"},{"description":"","displayCharacter":"AMP-binding domain","value":0,"secondaryStructure":"H"},{"description":"","displayCharacter":"AMP-binding domain","value":0,"secondaryStructure":"H"},{"description":"","displayCharacter":"AMP-binding domain","value":0,"secondaryStructure":"H"},{"description":"","displayCharacter":"AMP-binding domain","value":0,"secondaryStructure":"H"},{"description":"","displayCharacter":"AMP-binding domain","value":0,"secondaryStructure":"H"},{"description":"","displayCharacter":"AMP-binding domain","value":0,"secondaryStructure":"H"},{"description":"","displayCharacter":"AMP-binding domain","value":0,"secondaryStructure":"H"},{"description":"","displayCharacter":"AMP-binding domain","value":0,"secondaryStructure":"H"},{"description":"","displayCharacter":"AMP-binding domain","value":0,"secondaryStructure":"H"},{"description":"","displayCharacter":"AMP-binding domain","value":0,"secondaryStructure":"H"},{"description":"","displayCharacter":"AMP-binding domain","value":0,"secondaryStructure":"H"},{"description":"","displayCharacter":"AMP-binding domain","value":0,"secondaryStructure":"H"},{"description":"","displayCharacter":"AMP-binding domain","value":0,"secondaryStructure":"H"},{"description":"","displayCharacter":"AMP-binding domain","value":0,"secondaryStructure":"H"},{"description":"","displayCharacter":"AMP-binding domain","value":0,"secondaryStructure":"H"},{"description":"","displayCharacter":"AMP-binding domain","value":0,"secondaryStructure":"H"},{"description":"","displayCharacter":"AMP-binding domain","value":0,"secondaryStructure":"H"},{"description":"","displayCharacter":"AMP-binding domain","value":0,"secondaryStructure":"H"},{"description":"","displayCharacter":"AMP-binding domain","value":0,"secondaryStructure":"H"},{"description":"","displayCharacter":"AMP-binding domain","value":0,"secondaryStructure":"H"},{"description":"","displayCharacter":"AMP-binding domain","value":0,"secondaryStructure":"H"},{"description":"","displayCharacter":"AMP-binding domain","value":0,"secondaryStructure":"H"},{"description":"","displayCharacter":"AMP-binding domain","value":0,"secondaryStructure":"H"},{"description":"","displayCharacter":"AMP-binding domain","value":0,"secondaryStructure":"H"},{"description":"","displayCharacter":"AMP-binding domain","value":0,"secondaryStructure":"H"},{"description":"","displayCharacter":"AMP-binding domain","value":0,"secondaryStructure":"H"},{"description":"","displayCharacter":"AMP-binding domain","value":0,"secondaryStructure":"H"},{"description":"","displayCharacter":"AMP-binding domain","value":0,"secondaryStructure":"H"},{"description":"","displayCharacter":"AMP-binding domain","value":0,"secondaryStructure":"H"},{"description":"","displayCharacter":"AMP-binding domain","value":0,"secondaryStructure":"H"},{"description":"","displayCharacter":"AMP-binding domain","value":0,"secondaryStructure":"H"},{"description":"","displayCharacter":"AMP-binding domain","value":0,"secondaryStructure":"H"},{"description":"","displayCharacter":"AMP-binding domain","value":0,"secondaryStructure":"H"},{"description":"","displayCharacter":"AMP-binding domain","value":0,"secondaryStructure":"H"},{"description":"","displayCharacter":"AMP-binding domain","value":0,"secondaryStructure":"H"},{"description":"","displayCharacter":"AMP-binding domain","value":0,"secondaryStructure":"H"},{"description":"","displayCharacter":"AMP-binding domain","value":0,"secondaryStructure":"H"},{"description":"","displayCharacter":"AMP-binding domain","value":0,"secondaryStructure":"H"},{"description":"","displayCharacter":"AMP-binding domain","value":0,"secondaryStructure":"H"},{"description":"","displayCharacter":"AMP-binding domain","value":0,"secondaryStructure":"H"},{"description":"","displayCharacter":"AMP-binding domain","value":0,"secondaryStructure":"H"},{"description":"","displayCharacter":"AMP-binding domain","value":0,"secondaryStructure":"H"},{"description":"","displayCharacter":"AMP-binding domain","value":0,"secondaryStructure":"H"},{"description":"","displayCharacter":"AMP-binding domain","value":0,"secondaryStructure":"H"},{"description":"","displayCharacter":"AMP-binding domain","value":0,"secondaryStructure":"H"},{"description":"","displayCharacter":"AMP-binding domain","value":0,"secondaryStructure":"H"},{"description":"","displayCharacter":"AMP-binding domain","value":0,"secondaryStructure":"H"},{"description":"","displayCharacter":"AMP-binding domain","value":0,"secondaryStructure":"H"},{"description":"","displayCharacter":"AMP-binding domain","value":0,"secondaryStructure":"H"},{"description":"","displayCharacter":"AMP-binding domain","value":0,"secondaryStructure":"H"},{"description":"","displayCharacter":"AMP-binding domain","value":0,"secondaryStructure":"H"},{"description":"","displayCharacter":"AMP-binding domain","value":0,"secondaryStructure":"H"},{"description":"","displayCharacter":"AMP-binding domain","value":0,"secondaryStructure":"H"},{"description":"","displayCharacter":"AMP-binding domain","value":0,"secondaryStructure":"H"},{"description":"","displayCharacter":"AMP-binding domain","value":0,"secondaryStructure":"H"},{"description":"","displayCharacter":"AMP-binding domain","value":0,"secondaryStructure":"H"},{"description":"","displayCharacter":"AMP-binding domain","value":0,"secondaryStructure":"H"},{"description":"","displayCharacter":"AMP-binding domain","value":0,"secondaryStructure":"H"},{"description":"","displayCharacter":"AMP-binding domain","value":0,"secondaryStructure":"H"},{"description":"","displayCharacter":"AMP-binding domain","value":0,"secondaryStructure":"H"},{"description":"","displayCharacter":"AMP-binding domain","value":0,"secondaryStructure":"H"},{"description":"","displayCharacter":"AMP-binding domain","value":0,"secondaryStructure":"H"},{"description":"","displayCharacter":"AMP-binding domain","value":0,"secondaryStructure":"H"},{"description":"","displayCharacter":"AMP-binding domain","value":0,"secondaryStructure":"H"},{"description":"","displayCharacter":"AMP-binding domain","value":0,"secondaryStructure":"H"},{"description":"","displayCharacter":"AMP-binding domain","value":0,"secondaryStructure":"H"},{"description":"","displayCharacter":"AMP-binding domain","value":0,"secondaryStructure":"H"},{"description":"","displayCharacter":"AMP-binding domain","value":0,"secondaryStructure":"H"},{"description":"","displayCharacter":"AMP-binding domain","value":0,"secondaryStructure":"H"},{"description":"","displayCharacter":"AMP-binding domain","value":0,"secondaryStructure":"H"},{"description":"","displayCharacter":"AMP-binding domain","value":0,"secondaryStructure":"H"},{"description":"","displayCharacter":"AMP-binding domain","value":0,"secondaryStructure":"H"},{"description":"","displayCharacter":"AMP-binding domain","value":0,"secondaryStructure":"H"},{"description":"","displayCharacter":"AMP-binding domain","value":0,"secondaryStructure":"H"},{"description":"","displayCharacter":"AMP-binding domain","value":0,"secondaryStructure":"H"},{"description":"","displayCharacter":"AMP-binding domain","value":0,"secondaryStructure":"H"},{"description":"","displayCharacter":"AMP-binding domain","value":0,"secondaryStructure":"H"},{"description":"","displayCharacter":"AMP-binding domain","value":0,"secondaryStructure":"H"},{"description":"","displayCharacter":"AMP-binding domain","value":0,"secondaryStructure":"H"},{"description":"","displayCharacter":"AMP-binding domain","value":0,"secondaryStructure":"H"},{"description":"","displayCharacter":"AMP-binding domain","value":0,"secondaryStructure":"H"},{"description":"","displayCharacter":"AMP-binding domain","value":0,"secondaryStructure":"H"},{"description":"","displayCharacter":"AMP-binding domain","value":0,"secondaryStructure":"H"},{"description":"","displayCharacter":"AMP-binding domain","value":0,"secondaryStructure":"H"},{"description":"","displayCharacter":"AMP-binding domain","value":0,"secondaryStructure":"H"},{"description":"","displayCharacter":"AMP-binding domain","value":0,"secondaryStructure":"H"},{"description":"","displayCharacter":"AMP-binding domain","value":0,"secondaryStructure":"H"},{"description":"","displayCharacter":"AMP-binding domain","value":0,"secondaryStructure":"H"},{"description":"","displayCharacter":"AMP-binding domain","value":0,"secondaryStructure":"H"},{"description":"","displayCharacter":"AMP-binding domain","value":0,"secondaryStructure":"H"},{"description":"","displayCharacter":"AMP-binding domain","value":0,"secondaryStructure":"H"},{"description":"","displayCharacter":"AMP-binding domain","value":0,"secondaryStructure":"H"},{"description":"","displayCharacter":"AMP-binding domain","value":0,"secondaryStructure":"H"},{"description":"","displayCharacter":"AMP-binding domain","value":0,"secondaryStructure":"H"},{"description":"","displayCharacter":"AMP-binding domain","value":0,"secondaryStructure":"H"},{"description":"","displayCharacter":"AMP-binding domain","value":0,"secondaryStructure":"H"},{"description":"","displayCharacter":"AMP-binding domain","value":0,"secondaryStructure":"H"},{"description":"","displayCharacter":"AMP-binding domain","value":0,"secondaryStructure":"H"},{"description":"","displayCharacter":"AMP-binding domain","value":0,"secondaryStructure":"H"},{"description":"","displayCharacter":"AMP-binding domain","value":0,"secondaryStructure":"H"},{"description":"","displayCharacter":"AMP-binding domain","value":0,"secondaryStructure":"H"},{"description":"","displayCharacter":"AMP-binding domain","value":0,"secondaryStructure":"H"},{"description":"","displayCharacter":"AMP-binding domain","value":0,"secondaryStructure":"H"},{"description":"","displayCharacter":"AMP-binding domain","value":0,"secondaryStructure":"H"},{"description":"","displayCharacter":"AMP-binding domain","value":0,"secondaryStructure":"H"},{"description":"","displayCharacter":"AMP-binding domain","value":0,"secondaryStructure":"H"},{"description":"","displayCharacter":"AMP-binding domain","value":0,"secondaryStructure":"H"},{"description":"","displayCharacter":"AMP-binding domain","value":0,"secondaryStructure":"H"},{"description":"","displayCharacter":"AMP-binding domain","value":0,"secondaryStructure":"H"},{"description":"","displayCharacter":"AMP-binding domain","value":0,"secondaryStructure":"H"},{"description":"","displayCharacter":"AMP-binding domain","value":0,"secondaryStructure":"H"},{"description":"","displayCharacter":"AMP-binding domain","value":0,"secondaryStructure":"H"},{"description":"","displayCharacter":"AMP-binding domain","value":0,"secondaryStructure":"H"},{"description":"","displayCharacter":"AMP-binding domain","value":0,"secondaryStructure":"H"},{"description":"","displayCharacter":"AMP-binding domain","value":0,"secondaryStructure":"H"},{"description":"","displayCharacter":"AMP-binding domain","value":0,"secondaryStructure":"H"},{"description":"","displayCharacter":"AMP-binding domain","value":0,"secondaryStructure":"H"},{"description":"","displayCharacter":"AMP-binding domain","value":0,"secondaryStructure":"H"},{"description":"","displayCharacter":"AMP-binding domain","value":0,"secondaryStructure":"H"},{"description":"","displayCharacter":"AMP-binding domain","value":0,"secondaryStructure":"H"},{"description":"","displayCharacter":"AMP-binding domain","value":0,"secondaryStructure":"H"},{"description":"","displayCharacter":"AMP-binding domain","value":0,"secondaryStructure":"H"},{"description":"","displayCharacter":"AMP-binding domain","value":0,"secondaryStructure":"H"},{"description":"","displayCharacter":"AMP-binding domain","value":0,"secondaryStructure":"H"},{"description":"","displayCharacter":"AMP-binding domain","value":0,"secondaryStructure":"H"},{"description":"","displayCharacter":"AMP-binding domain","value":0,"secondaryStructure":"H"},{"description":"","displayCharacter":"AMP-binding domain","value":0,"secondaryStructure":"H"},{"description":"","displayCharacter":"AMP-binding domain","value":0,"secondaryStructure":"H"},{"description":"","displayCharacter":"AMP-binding domain","value":0,"secondaryStructure":"H"},{"description":"","displayCharacter":"AMP-binding domain","value":0,"secondaryStructure":"H"},{"description":"","displayCharacter":"AMP-binding domain","value":0,"secondaryStructure":"H"},{"description":"","displayCharacter":"AMP-binding domain","value":0,"secondaryStructure":"H"},{"description":"","displayCharacter":"AMP-binding domain","value":0,"secondaryStructure":"H"},{"description":"","displayCharacter":"AMP-binding domain","value":0,"secondaryStructure":"H"},{"description":"","displayCharacter":"AMP-binding domain","value":0,"secondaryStructure":"H"},{"description":"","displayCharacter":"AMP-binding domain","value":0,"secondaryStructure":"H"},{"description":"","displayCharacter":"AMP-binding domain","value":0,"secondaryStructure":"H"},{"description":"","displayCharacter":"AMP-binding domain","value":0,"secondaryStructure":"H"},{"description":"","displayCharacter":"AMP-binding domain","value":0,"secondaryStructure":"H"},{"description":"","displayCharacter":"AMP-binding domain","value":0,"secondaryStructure":"H"},{"description":"","displayCharacter":"AMP-binding domain","value":0,"secondaryStructure":"H"},{"description":"","displayCharacter":"AMP-binding domain","value":0,"secondaryStructure":"H"},{"description":"","displayCharacter":"AMP-binding domain","value":0,"secondaryStructure":"H"},{"description":"","displayCharacter":"AMP-binding domain","value":0,"secondaryStructure":"H"},{"description":"","displayCharacter":"AMP-binding domain","value":0,"secondaryStructure":"H"},{"description":"","displayCharacter":"AMP-binding domain","value":0,"secondaryStructure":"H"},{"description":"","displayCharacter":"AMP-binding domain","value":0,"secondaryStructure":"H"},{"description":"","displayCharacter":"AMP-binding domain","value":0,"secondaryStructure":"H"},{"description":"","displayCharacter":"AMP-binding domain","value":0,"secondaryStructure":"H"},{"description":"","displayCharacter":"AMP-binding domain","value":0,"secondaryStructure":"H"},{"description":"","displayCharacter":"AMP-binding domain","value":0,"secondaryStructure":"H"},{"description":"","displayCharacter":"AMP-binding domain","value":0,"secondaryStructure":"H"},{"description":"","displayCharacter":"AMP-binding domain","value":0,"secondaryStructure":"H"},{"description":"","displayCharacter":"AMP-binding domain","value":0,"secondaryStructure":"H"},{"description":"","displayCharacter":"AMP-binding domain","value":0,"secondaryStructure":"H"},{"description":"","displayCharacter":"AMP-binding domain","value":0,"secondaryStructure":"H"},{"description":"","displayCharacter":"AMP-binding domain","value":0,"secondaryStructure":"H"},{"description":"","displayCharacter":"AMP-binding domain","value":0,"secondaryStructure":"H"},{"description":"","displayCharacter":"AMP-binding domain","value":0,"secondaryStructure":"H"},{"description":"","displayCharacter":"AMP-binding domain","value":0,"secondaryStructure":"H"},{"description":"","displayCharacter":"AMP-binding domain","value":0,"secondaryStructure":"H"},{"description":"","displayCharacter":"AMP-binding domain","value":0,"secondaryStructure":"H"},{"description":"","displayCharacter":"AMP-binding domain","value":0,"secondaryStructure":"H"},{"description":"","displayCharacter":"AMP-binding domain","value":0,"secondaryStructure":"H"},{"description":"","displayCharacter":"AMP-binding domain","value":0,"secondaryStructure":"H"},{"description":"","displayCharacter":"AMP-binding domain","value":0,"secondaryStructure":"H"},{"description":"","displayCharacter":"AMP-binding domain","value":0,"secondaryStructure":"H"},{"description":"","displayCharacter":"AMP-binding domain","value":0,"secondaryStructure":"H"},{"description":"","displayCharacter":"AMP-binding domain","value":0,"secondaryStructure":"H"},{"description":"","displayCharacter":"AMP-binding domain","value":0,"secondaryStructure":"H"},{"description":"","displayCharacter":"AMP-binding domain","value":0,"secondaryStructure":"H"},{"description":"","displayCharacter":"AMP-binding domain","value":0,"secondaryStructure":"H"},{"description":"","displayCharacter":"AMP-binding domain","value":0,"secondaryStructure":"H"},{"description":"","displayCharacter":"AMP-binding domain","value":0,"secondaryStructure":"H"},{"description":"","displayCharacter":"AMP-binding domain","value":0,"secondaryStructure":"H"},{"description":"","displayCharacter":"AMP-binding domain","value":0,"secondaryStructure":"H"},{"description":"","displayCharacter":"AMP-binding domain","value":0,"secondaryStructure":"H"},{"description":"","displayCharacter":"AMP-binding domain","value":0,"secondaryStructure":"H"},{"description":"","displayCharacter":"AMP-binding domain","value":0,"secondaryStructure":"H"},{"description":"","displayCharacter":"AMP-binding domain","value":0,"secondaryStructure":"H"},{"description":"","displayCharacter":"AMP-binding domain","value":0,"secondaryStructure":"H"},{"description":"","displayCharacter":"AMP-binding domain","value":0,"secondaryStructure":"H"},{"description":"","displayCharacter":"AMP-binding domain","value":0,"secondaryStructure":"H"},{"description":"","displayCharacter":"AMP-binding domain","value":0,"secondaryStructure":"H"},{"description":"","displayCharacter":"AMP-binding domain","value":0,"secondaryStructure":"H"},{"description":"","displayCharacter":"AMP-binding domain","value":0,"secondaryStructure":"H"},{"description":"","displayCharacter":"AMP-binding domain","value":0,"secondaryStructure":"H"},{"description":"","displayCharacter":"AMP-binding domain","value":0,"secondaryStructure":"H"},{"description":"","displayCharacter":"AMP-binding domain","value":0,"secondaryStructure":"H"},{"description":"","displayCharacter":"AMP-binding domain","value":0,"secondaryStructure":"H"},{"description":"","displayCharacter":"AMP-binding domain","value":0,"secondaryStructure":"H"},{"description":"","displayCharacter":"AMP-binding domain","value":0,"secondaryStructure":"H"},{"description":"","displayCharacter":"AMP-binding domain","value":0,"secondaryStructure":"H"},{"description":"","displayCharacter":"AMP-binding domain","value":0,"secondaryStructure":"H"},{"description":"","displayCharacter":"AMP-binding domain","value":0,"secondaryStructure":"H"},{"description":"","displayCharacter":"AMP-binding domain","value":0,"secondaryStructure":"H"},{"description":"","displayCharacter":"AMP-binding domain","value":0,"secondaryStructure":"H"},{"description":"","displayCharacter":"AMP-binding domain","value":0,"secondaryStructure":"H"},{"description":"","displayCharacter":"AMP-binding domain","value":0,"secondaryStructure":"H"},{"description":"","displayCharacter":"AMP-binding domain","value":0,"secondaryStructure":"H"},{"description":"","displayCharacter":"AMP-binding domain","value":0,"secondaryStructure":"H"},{"description":"","displayCharacter":"AMP-binding domain","value":0,"secondaryStructure":"H"},{"description":"","displayCharacter":"AMP-binding domain","value":0,"secondaryStructure":"H"},{"description":"","displayCharacter":"AMP-binding domain","value":0,"secondaryStructure":"H"},{"description":"","displayCharacter":"AMP-binding domain","value":0,"secondaryStructure":"H"},{"description":"","displayCharacter":"AMP-binding domain","value":0,"secondaryStructure":"H"},{"description":"","displayCharacter":"AMP-binding domain","value":0,"secondaryStructure":"H"},{"description":"","displayCharacter":"AMP-binding domain","value":0,"secondaryStructure":"H"},{"description":"","displayCharacter":"AMP-binding domain","value":0,"secondaryStructure":"H"},{"description":"","displayCharacter":"AMP-binding domain","value":0,"secondaryStructure":"H"},{"description":"","displayCharacter":"AMP-binding domain","value":0,"secondaryStructure":"H"},{"description":"","displayCharacter":"AMP-binding domain","value":0,"secondaryStructure":"H"},{"description":"","displayCharacter":"AMP-binding domain","value":0,"secondaryStructure":"H"},{"description":"","displayCharacter":"AMP-binding domain","value":0,"secondaryStructure":"H"},{"description":"","displayCharacter":"AMP-binding domain","value":0,"secondaryStructure":"H"},{"description":"","displayCharacter":"AMP-binding domain","value":0,"secondaryStructure":"H"},{"description":"","displayCharacter":"AMP-binding domain","value":0,"secondaryStructure":"H"},{"description":"","displayCharacter":"AMP-binding domain","value":0,"secondaryStructure":"H"},{"description":"","displayCharacter":"AMP-binding domain","value":0,"secondaryStructure":"H"},{"description":"","displayCharacter":"AMP-binding domain","value":0,"secondaryStructure":"H"},{"description":"","displayCharacter":"AMP-binding domain","value":0,"secondaryStructure":"H"},{"description":"","displayCharacter":"AMP-binding domain","value":0,"secondaryStructure":"H"},{"description":"","displayCharacter":"AMP-binding domain","value":0,"secondaryStructure":"H"},{"description":"","displayCharacter":"AMP-binding domain","value":0,"secondaryStructure":"H"},{"description":"","displayCharacter":"AMP-binding domain","value":0,"secondaryStructure":"H"},{"description":"","displayCharacter":"AMP-binding domain","value":0,"secondaryStructure":"H"},{"description":"","displayCharacter":"AMP-binding domain","value":0,"secondaryStructure":"H"},{"description":"","displayCharacter":"AMP-binding domain","value":0,"secondaryStructure":"H"},{"description":"","displayCharacter":"AMP-binding domain","value":0,"secondaryStructure":"H"},{"description":"","displayCharacter":"AMP-binding domain","value":0,"secondaryStructure":"H"},{"description":"","displayCharacter":"AMP-binding domain","value":0,"secondaryStructure":"H"},{"description":"","displayCharacter":"AMP-binding domain","value":0,"secondaryStructure":"H"},{"description":"","displayCharacter":"AMP-binding domain","value":0,"secondaryStructure":"H"},{"description":"","displayCharacter":"AMP-binding domain","value":0,"secondaryStructure":"H"},{"value":0,"secondaryStructure":"\u0000"},{"value":0,"secondaryStructure":"\u0000"},{"value":0,"secondaryStructure":"\u0000"},{"value":0,"secondaryStructure":"\u0000"},{"value":0,"secondaryStructure":"\u0000"},{"value":0,"secondaryStructure":"\u0000"},{"value":0,"secondaryStructure":"\u0000"},{"value":0,"secondaryStructure":"\u0000"},{"value":0,"secondaryStructure":"\u0000"},{"value":0,"secondaryStructure":"\u0000"},{"value":0,"secondaryStructure":"\u0000"},{"value":0,"secondaryStructure":"\u0000"},{"value":0,"secondaryStructure":"\u0000"},{"value":0,"secondaryStructure":"\u0000"},{"value":0,"secondaryStructure":"\u0000"},{"value":0,"secondaryStructure":"\u0000"},{"value":0,"secondaryStructure":"\u0000"},{"value":0,"secondaryStructure":"\u0000"},{"value":0,"secondaryStructure":"\u0000"},{"value":0,"secondaryStructure":"\u0000"},{"value":0,"secondaryStructure":"\u0000"},{"value":0,"secondaryStructure":"\u0000"},{"value":0,"secondaryStructure":"\u0000"},{"value":0,"secondaryStructure":"\u0000"},{"value":0,"secondaryStructure":"\u0000"},{"value":0,"secondaryStructure":"\u0000"},{"value":0,"secondaryStructure":"\u0000"},{"description":"","displayCharacter":"ACP","value":0,"secondaryStructure":"H"},{"description":"","displayCharacter":"ACP","value":0,"secondaryStructure":"H"},{"description":"","displayCharacter":"ACP","value":0,"secondaryStructure":"H"},{"description":"","displayCharacter":"ACP","value":0,"secondaryStructure":"H"},{"description":"","displayCharacter":"ACP","value":0,"secondaryStructure":"H"},{"description":"","displayCharacter":"ACP","value":0,"secondaryStructure":"H"},{"description":"","displayCharacter":"ACP","value":0,"secondaryStructure":"H"},{"description":"","displayCharacter":"ACP","value":0,"secondaryStructure":"H"},{"description":"","displayCharacter":"ACP","value":0,"secondaryStructure":"H"},{"description":"","displayCharacter":"ACP","value":0,"secondaryStructure":"H"},{"description":"","displayCharacter":"ACP","value":0,"secondaryStructure":"H"},{"description":"","displayCharacter":"ACP","value":0,"secondaryStructure":"H"},{"description":"","displayCharacter":"ACP","value":0,"secondaryStructure":"H"},{"description":"","displayCharacter":"ACP","value":0,"secondaryStructure":"H"},{"description":"","displayCharacter":"ACP","value":0,"secondaryStructure":"H"},{"description":"","displayCharacter":"ACP","value":0,"secondaryStructure":"H"},{"description":"","displayCharacter":"ACP","value":0,"secondaryStructure":"H"},{"description":"","displayCharacter":"ACP","value":0,"secondaryStructure":"H"},{"description":"","displayCharacter":"ACP","value":0,"secondaryStructure":"H"},{"description":"","displayCharacter":"ACP","value":0,"secondaryStructure":"H"},{"description":"","displayCharacter":"ACP","value":0,"secondaryStructure":"H"},{"description":"","displayCharacter":"ACP","value":0,"secondaryStructure":"H"},{"description":"","displayCharacter":"ACP","value":0,"secondaryStructure":"H"},{"description":"","displayCharacter":"ACP","value":0,"secondaryStructure":"H"},{"description":"","displayCharacter":"ACP","value":0,"secondaryStructure":"H"},{"description":"","displayCharacter":"ACP","value":0,"secondaryStructure":"H"},{"description":"","displayCharacter":"ACP","value":0,"secondaryStructure":"H"},{"description":"","displayCharacter":"ACP","value":0,"secondaryStructure":"H"},{"description":"","displayCharacter":"ACP","value":0,"secondaryStructure":"H"},{"description":"","displayCharacter":"ACP","value":0,"secondaryStructure":"H"},{"description":"","displayCharacter":"ACP","value":0,"secondaryStructure":"H"},{"description":"","displayCharacter":"ACP","value":0,"secondaryStructure":"H"},{"description":"","displayCharacter":"ACP","value":0,"secondaryStructure":"H"},{"description":"","displayCharacter":"ACP","value":0,"secondaryStructure":"H"},{"description":"","displayCharacter":"ACP","value":0,"secondaryStructure":"H"},{"description":"","displayCharacter":"ACP","value":0,"secondaryStructure":"H"},{"description":"","displayCharacter":"ACP","value":0,"secondaryStructure":"H"},{"description":"","displayCharacter":"ACP","value":0,"secondaryStructure":"H"},{"description":"","displayCharacter":"ACP","value":0,"secondaryStructure":"H"},{"description":"","displayCharacter":"ACP","value":0,"secondaryStructure":"H"},{"description":"","displayCharacter":"ACP","value":0,"secondaryStructure":"H"},{"description":"","displayCharacter":"ACP","value":0,"secondaryStructure":"H"},{"description":"","displayCharacter":"ACP","value":0,"secondaryStructure":"H"},{"description":"","displayCharacter":"ACP","value":0,"secondaryStructure":"H"},{"description":"","displayCharacter":"ACP","value":0,"secondaryStructure":"H"},{"description":"","displayCharacter":"ACP","value":0,"secondaryStructure":"H"},{"description":"","displayCharacter":"ACP","value":0,"secondaryStructure":"H"},{"description":"","displayCharacter":"ACP","value":0,"secondaryStructure":"H"},{"description":"","displayCharacter":"ACP","value":0,"secondaryStructure":"H"},{"description":"","displayCharacter":"ACP","value":0,"secondaryStructure":"H"},{"description":"","displayCharacter":"ACP","value":0,"secondaryStructure":"H"},{"description":"","displayCharacter":"ACP","value":0,"secondaryStructure":"H"},{"description":"","displayCharacter":"ACP","value":0,"secondaryStructure":"H"},{"description":"","displayCharacter":"ACP","value":0,"secondaryStructure":"H"},{"description":"","displayCharacter":"ACP","value":0,"secondaryStructure":"H"},{"description":"","displayCharacter":"ACP","value":0,"secondaryStructure":"H"},{"description":"","displayCharacter":"ACP","value":0,"secondaryStructure":"H"},{"description":"","displayCharacter":"ACP","value":0,"secondaryStructure":"H"},{"description":"","displayCharacter":"ACP","value":0,"secondaryStructure":"H"},{"description":"","displayCharacter":"ACP","value":0,"secondaryStructure":"H"},{"description":"","displayCharacter":"ACP","value":0,"secondaryStructure":"H"},{"description":"","displayCharacter":"ACP","value":0,"secondaryStructure":"H"},{"description":"","displayCharacter":"ACP","value":0,"secondaryStructure":"H"},{"description":"","displayCharacter":"ACP","value":0,"secondaryStructure":"H"},{"description":"","displayCharacter":"ACP","value":0,"secondaryStructure":"H"},{"description":"","displayCharacter":"ACP","value":0,"secondaryStructure":"H"},{"description":"","displayCharacter":"ACP","value":0,"secondaryStructure":"H"},{"description":"","displayCharacter":"ACP","value":0,"secondaryStructure":"H"},{"description":"","displayCharacter":"ACP","value":0,"secondaryStructure":"H"},{"description":"","displayCharacter":"ACP","value":0,"secondaryStructure":"H"},{"description":"","displayCharacter":"ACP","value":0,"secondaryStructure":"H"},{"description":"","displayCharacter":"ACP","value":0,"secondaryStructure":"H"},{"description":"","displayCharacter":"ACP","value":0,"secondaryStructure":"H"},{"value":0,"secondaryStructure":"\u0000"},{"value":0,"secondaryStructure":"\u0000"},{"value":0,"secondaryStructure":"\u0000"},{"value":0,"secondaryStructure":"\u0000"},{"value":0,"secondaryStructure":"\u0000"},{"value":0,"secondaryStructure":"\u0000"},{"value":0,"secondaryStructure":"\u0000"},{"value":0,"secondaryStructure":"\u0000"},{"value":0,"secondaryStructure":"\u0000"},{"value":0,"secondaryStructure":"\u0000"},{"value":0,"secondaryStructure":"\u0000"},{"value":0,"secondaryStructure":"\u0000"},{"value":0,"secondaryStructure":"\u0000"},{"value":0,"secondaryStructure":"\u0000"},{"value":0,"secondaryStructure":"\u0000"},{"value":0,"secondaryStructure":"\u0000"},{"value":0,"secondaryStructure":"\u0000"},{"value":0,"secondaryStructure":"\u0000"},{"value":0,"secondaryStructure":"\u0000"},{"value":0,"secondaryStructure":"\u0000"},{"value":0,"secondaryStructure":"\u0000"},{"value":0,"secondaryStructure":"\u0000"},{"description":"","displayCharacter":"Ketosynthase domain","value":0,"secondaryStructure":"H"},{"description":"","displayCharacter":"Ketosynthase domain","value":0,"secondaryStructure":"H"},{"description":"","displayCharacter":"Ketosynthase domain","value":0,"secondaryStructure":"H"},{"description":"","displayCharacter":"Ketosynthase domain","value":0,"secondaryStructure":"H"},{"description":"","displayCharacter":"Ketosynthase domain","value":0,"secondaryStructure":"H"},{"description":"","displayCharacter":"Ketosynthase domain","value":0,"secondaryStructure":"H"},{"description":"","displayCharacter":"Ketosynthase domain","value":0,"secondaryStructure":"H"},{"description":"","displayCharacter":"Ketosynthase domain","value":0,"secondaryStructure":"H"},{"description":"","displayCharacter":"Ketosynthase domain","value":0,"secondaryStructure":"H"},{"description":"","displayCharacter":"Ketosynthase domain","value":0,"secondaryStructure":"H"},{"description":"","displayCharacter":"Ketosynthase domain","value":0,"secondaryStructure":"H"},{"description":"","displayCharacter":"Ketosynthase domain","value":0,"secondaryStructure":"H"},{"description":"","displayCharacter":"Ketosynthase domain","value":0,"secondaryStructure":"H"},{"description":"","displayCharacter":"Ketosynthase domain","value":0,"secondaryStructure":"H"},{"description":"","displayCharacter":"Ketosynthase domain","value":0,"secondaryStructure":"H"},{"description":"","displayCharacter":"Ketosynthase domain","value":0,"secondaryStructure":"H"},{"description":"","displayCharacter":"Ketosynthase domain","value":0,"secondaryStructure":"H"},{"description":"","displayCharacter":"Ketosynthase domain","value":0,"secondaryStructure":"H"},{"description":"","displayCharacter":"Ketosynthase domain","value":0,"secondaryStructure":"H"},{"description":"","displayCharacter":"Ketosynthase domain","value":0,"secondaryStructure":"H"},{"description":"","displayCharacter":"Ketosynthase domain","value":0,"secondaryStructure":"H"},{"description":"","displayCharacter":"Ketosynthase domain","value":0,"secondaryStructure":"H"},{"description":"","displayCharacter":"Ketosynthase domain","value":0,"secondaryStructure":"H"},{"description":"","displayCharacter":"Ketosynthase domain","value":0,"secondaryStructure":"H"},{"description":"","displayCharacter":"Ketosynthase domain","value":0,"secondaryStructure":"H"},{"description":"","displayCharacter":"Ketosynthase domain","value":0,"secondaryStructure":"H"},{"description":"","displayCharacter":"Ketosynthase domain","value":0,"secondaryStructure":"H"},{"description":"","displayCharacter":"Ketosynthase domain","value":0,"secondaryStructure":"H"},{"description":"","displayCharacter":"Ketosynthase domain","value":0,"secondaryStructure":"H"},{"description":"","displayCharacter":"Ketosynthase domain","value":0,"secondaryStructure":"H"},{"description":"","displayCharacter":"Ketosynthase domain","value":0,"secondaryStructure":"H"},{"description":"","displayCharacter":"Ketosynthase domain","value":0,"secondaryStructure":"H"},{"description":"","displayCharacter":"Ketosynthase domain","value":0,"secondaryStructure":"H"},{"description":"","displayCharacter":"Ketosynthase domain","value":0,"secondaryStructure":"H"},{"description":"","displayCharacter":"Ketosynthase domain","value":0,"secondaryStructure":"H"},{"description":"","displayCharacter":"Ketosynthase domain","value":0,"secondaryStructure":"H"},{"description":"","displayCharacter":"Ketosynthase domain","value":0,"secondaryStructure":"H"},{"description":"","displayCharacter":"Ketosynthase domain","value":0,"secondaryStructure":"H"},{"description":"","displayCharacter":"Ketosynthase domain","value":0,"secondaryStructure":"H"},{"description":"","displayCharacter":"Ketosynthase domain","value":0,"secondaryStructure":"H"},{"description":"","displayCharacter":"Ketosynthase domain","value":0,"secondaryStructure":"H"},{"description":"","displayCharacter":"Ketosynthase domain","value":0,"secondaryStructure":"H"},{"description":"","displayCharacter":"Ketosynthase domain","value":0,"secondaryStructure":"H"},{"description":"","displayCharacter":"Ketosynthase domain","value":0,"secondaryStructure":"H"},{"description":"","displayCharacter":"Ketosynthase domain","value":0,"secondaryStructure":"H"},{"description":"","displayCharacter":"Ketosynthase domain","value":0,"secondaryStructure":"H"},{"description":"","displayCharacter":"Ketosynthase domain","value":0,"secondaryStructure":"H"},{"description":"","displayCharacter":"Ketosynthase domain","value":0,"secondaryStructure":"H"},{"description":"","displayCharacter":"Ketosynthase domain","value":0,"secondaryStructure":"H"},{"description":"","displayCharacter":"Ketosynthase domain","value":0,"secondaryStructure":"H"},{"description":"","displayCharacter":"Ketosynthase domain","value":0,"secondaryStructure":"H"},{"description":"","displayCharacter":"Ketosynthase domain","value":0,"secondaryStructure":"H"},{"description":"","displayCharacter":"Ketosynthase domain","value":0,"secondaryStructure":"H"},{"description":"","displayCharacter":"Ketosynthase domain","value":0,"secondaryStructure":"H"},{"description":"","displayCharacter":"Ketosynthase domain","value":0,"secondaryStructure":"H"},{"description":"","displayCharacter":"Ketosynthase domain","value":0,"secondaryStructure":"H"},{"description":"","displayCharacter":"Ketosynthase domain","value":0,"secondaryStructure":"H"},{"description":"","displayCharacter":"Ketosynthase domain","value":0,"secondaryStructure":"H"},{"description":"","displayCharacter":"Ketosynthase domain","value":0,"secondaryStructure":"H"},{"description":"","displayCharacter":"Ketosynthase domain","value":0,"secondaryStructure":"H"},{"description":"","displayCharacter":"Ketosynthase domain","value":0,"secondaryStructure":"H"},{"description":"","displayCharacter":"Ketosynthase domain","value":0,"secondaryStructure":"H"},{"description":"","displayCharacter":"Ketosynthase domain","value":0,"secondaryStructure":"H"},{"description":"","displayCharacter":"Ketosynthase domain","value":0,"secondaryStructure":"H"},{"description":"","displayCharacter":"Ketosynthase domain","value":0,"secondaryStructure":"H"},{"description":"","displayCharacter":"Ketosynthase domain","value":0,"secondaryStructure":"H"},{"description":"","displayCharacter":"Ketosynthase domain","value":0,"secondaryStructure":"H"},{"description":"","displayCharacter":"Ketosynthase domain","value":0,"secondaryStructure":"H"},{"description":"","displayCharacter":"Ketosynthase domain","value":0,"secondaryStructure":"H"},{"description":"","displayCharacter":"Ketosynthase domain","value":0,"secondaryStructure":"H"},{"description":"","displayCharacter":"Ketosynthase domain","value":0,"secondaryStructure":"H"},{"description":"","displayCharacter":"Ketosynthase domain","value":0,"secondaryStructure":"H"},{"description":"","displayCharacter":"Ketosynthase domain","value":0,"secondaryStructure":"H"},{"description":"","displayCharacter":"Ketosynthase domain","value":0,"secondaryStructure":"H"},{"description":"","displayCharacter":"Ketosynthase domain","value":0,"secondaryStructure":"H"},{"description":"","displayCharacter":"Ketosynthase domain","value":0,"secondaryStructure":"H"},{"description":"","displayCharacter":"Ketosynthase domain","value":0,"secondaryStructure":"H"},{"description":"","displayCharacter":"Ketosynthase domain","value":0,"secondaryStructure":"H"},{"description":"","displayCharacter":"Ketosynthase domain","value":0,"secondaryStructure":"H"},{"description":"","displayCharacter":"Ketosynthase domain","value":0,"secondaryStructure":"H"},{"description":"","displayCharacter":"Ketosynthase domain","value":0,"secondaryStructure":"H"},{"description":"","displayCharacter":"Ketosynthase domain","value":0,"secondaryStructure":"H"},{"description":"","displayCharacter":"Ketosynthase domain","value":0,"secondaryStructure":"H"},{"description":"","displayCharacter":"Ketosynthase domain","value":0,"secondaryStructure":"H"},{"description":"","displayCharacter":"Ketosynthase domain","value":0,"secondaryStructure":"H"},{"description":"","displayCharacter":"Ketosynthase domain","value":0,"secondaryStructure":"H"},{"description":"","displayCharacter":"Ketosynthase domain","value":0,"secondaryStructure":"H"},{"description":"","displayCharacter":"Ketosynthase domain","value":0,"secondaryStructure":"H"},{"description":"","displayCharacter":"Ketosynthase domain","value":0,"secondaryStructure":"H"},{"description":"","displayCharacter":"Ketosynthase domain","value":0,"secondaryStructure":"H"},{"description":"","displayCharacter":"Ketosynthase domain","value":0,"secondaryStructure":"H"},{"description":"","displayCharacter":"Ketosynthase domain","value":0,"secondaryStructure":"H"},{"description":"","displayCharacter":"Ketosynthase domain","value":0,"secondaryStructure":"H"},{"description":"","displayCharacter":"Ketosynthase domain","value":0,"secondaryStructure":"H"},{"description":"","displayCharacter":"Ketosynthase domain","value":0,"secondaryStructure":"H"},{"description":"","displayCharacter":"Ketosynthase domain","value":0,"secondaryStructure":"H"},{"description":"","displayCharacter":"Ketosynthase domain","value":0,"secondaryStructure":"H"},{"description":"","displayCharacter":"Ketosynthase domain","value":0,"secondaryStructure":"H"},{"description":"","displayCharacter":"Ketosynthase domain","value":0,"secondaryStructure":"H"},{"description":"","displayCharacter":"Ketosynthase domain","value":0,"secondaryStructure":"H"},{"description":"","displayCharacter":"Ketosynthase domain","value":0,"secondaryStructure":"H"},{"description":"","displayCharacter":"Ketosynthase domain","value":0,"secondaryStructure":"H"},{"description":"","displayCharacter":"Ketosynthase domain","value":0,"secondaryStructure":"H"},{"description":"","displayCharacter":"Ketosynthase domain","value":0,"secondaryStructure":"H"},{"description":"","displayCharacter":"Ketosynthase domain","value":0,"secondaryStructure":"H"},{"description":"","displayCharacter":"Ketosynthase domain","value":0,"secondaryStructure":"H"},{"description":"","displayCharacter":"Ketosynthase domain","value":0,"secondaryStructure":"H"},{"description":"","displayCharacter":"Ketosynthase domain","value":0,"secondaryStructure":"H"},{"description":"","displayCharacter":"Ketosynthase domain","value":0,"secondaryStructure":"H"},{"description":"","displayCharacter":"Ketosynthase domain","value":0,"secondaryStructure":"H"},{"description":"","displayCharacter":"Ketosynthase domain","value":0,"secondaryStructure":"H"},{"description":"","displayCharacter":"Ketosynthase domain","value":0,"secondaryStructure":"H"},{"description":"","displayCharacter":"Ketosynthase domain","value":0,"secondaryStructure":"H"},{"description":"","displayCharacter":"Ketosynthase domain","value":0,"secondaryStructure":"H"},{"description":"","displayCharacter":"Ketosynthase domain","value":0,"secondaryStructure":"H"},{"description":"","displayCharacter":"Ketosynthase domain","value":0,"secondaryStructure":"H"},{"description":"","displayCharacter":"Ketosynthase domain","value":0,"secondaryStructure":"H"},{"description":"","displayCharacter":"Ketosynthase domain","value":0,"secondaryStructure":"H"},{"description":"","displayCharacter":"Ketosynthase domain","value":0,"secondaryStructure":"H"},{"description":"","displayCharacter":"Ketosynthase domain","value":0,"secondaryStructure":"H"},{"description":"","displayCharacter":"Ketosynthase domain","value":0,"secondaryStructure":"H"},{"description":"","displayCharacter":"Ketosynthase domain","value":0,"secondaryStructure":"H"},{"description":"","displayCharacter":"Ketosynthase domain","value":0,"secondaryStructure":"H"},{"description":"","displayCharacter":"Ketosynthase domain","value":0,"secondaryStructure":"H"},{"description":"","displayCharacter":"Ketosynthase domain","value":0,"secondaryStructure":"H"},{"description":"","displayCharacter":"Ketosynthase domain","value":0,"secondaryStructure":"H"},{"description":"","displayCharacter":"Ketosynthase domain","value":0,"secondaryStructure":"H"},{"description":"","displayCharacter":"Ketosynthase domain","value":0,"secondaryStructure":"H"},{"description":"","displayCharacter":"Ketosynthase domain","value":0,"secondaryStructure":"H"},{"description":"","displayCharacter":"Ketosynthase domain","value":0,"secondaryStructure":"H"},{"description":"","displayCharacter":"Ketosynthase domain","value":0,"secondaryStructure":"H"},{"description":"","displayCharacter":"Ketosynthase domain","value":0,"secondaryStructure":"H"},{"description":"","displayCharacter":"Ketosynthase domain","value":0,"secondaryStructure":"H"},{"description":"","displayCharacter":"Ketosynthase domain","value":0,"secondaryStructure":"H"},{"description":"","displayCharacter":"Ketosynthase domain","value":0,"secondaryStructure":"H"},{"description":"","displayCharacter":"Ketosynthase domain","value":0,"secondaryStructure":"H"},{"description":"","displayCharacter":"Ketosynthase domain","value":0,"secondaryStructure":"H"},{"description":"","displayCharacter":"Ketosynthase domain","value":0,"secondaryStructure":"H"},{"description":"","displayCharacter":"Ketosynthase domain","value":0,"secondaryStructure":"H"},{"description":"","displayCharacter":"Ketosynthase domain","value":0,"secondaryStructure":"H"},{"description":"","displayCharacter":"Ketosynthase domain","value":0,"secondaryStructure":"H"},{"description":"","displayCharacter":"Ketosynthase domain","value":0,"secondaryStructure":"H"},{"description":"","displayCharacter":"Ketosynthase domain","value":0,"secondaryStructure":"H"},{"description":"","displayCharacter":"Ketosynthase domain","value":0,"secondaryStructure":"H"},{"description":"","displayCharacter":"Ketosynthase domain","value":0,"secondaryStructure":"H"},{"description":"","displayCharacter":"Ketosynthase domain","value":0,"secondaryStructure":"H"},{"description":"","displayCharacter":"Ketosynthase domain","value":0,"secondaryStructure":"H"},{"description":"","displayCharacter":"Ketosynthase domain","value":0,"secondaryStructure":"H"},{"description":"","displayCharacter":"Ketosynthase domain","value":0,"secondaryStructure":"H"},{"description":"","displayCharacter":"Ketosynthase domain","value":0,"secondaryStructure":"H"},{"description":"","displayCharacter":"Ketosynthase domain","value":0,"secondaryStructure":"H"},{"description":"","displayCharacter":"Ketosynthase domain","value":0,"secondaryStructure":"H"},{"description":"","displayCharacter":"Ketosynthase domain","value":0,"secondaryStructure":"H"},{"description":"","displayCharacter":"Ketosynthase domain","value":0,"secondaryStructure":"H"},{"description":"","displayCharacter":"Ketosynthase domain","value":0,"secondaryStructure":"H"},{"description":"","displayCharacter":"Ketosynthase domain","value":0,"secondaryStructure":"H"},{"description":"","displayCharacter":"Ketosynthase domain","value":0,"secondaryStructure":"H"},{"description":"","displayCharacter":"Ketosynthase domain","value":0,"secondaryStructure":"H"},{"description":"","displayCharacter":"Ketosynthase domain","value":0,"secondaryStructure":"H"},{"description":"","displayCharacter":"Ketosynthase domain","value":0,"secondaryStructure":"H"},{"description":"","displayCharacter":"Ketosynthase domain","value":0,"secondaryStructure":"H"},{"description":"","displayCharacter":"Ketosynthase domain","value":0,"secondaryStructure":"H"},{"description":"","displayCharacter":"Ketosynthase domain","value":0,"secondaryStructure":"H"},{"description":"","displayCharacter":"Ketosynthase domain","value":0,"secondaryStructure":"H"},{"description":"","displayCharacter":"Ketosynthase domain","value":0,"secondaryStructure":"H"},{"description":"","displayCharacter":"Ketosynthase domain","value":0,"secondaryStructure":"H"},{"description":"","displayCharacter":"Ketosynthase domain","value":0,"secondaryStructure":"H"},{"description":"","displayCharacter":"Ketosynthase domain","value":0,"secondaryStructure":"H"},{"description":"","displayCharacter":"Ketosynthase domain","value":0,"secondaryStructure":"H"},{"description":"","displayCharacter":"Ketosynthase domain","value":0,"secondaryStructure":"H"},{"description":"","displayCharacter":"Ketosynthase domain","value":0,"secondaryStructure":"H"},{"description":"","displayCharacter":"Ketosynthase domain","value":0,"secondaryStructure":"H"},{"description":"","displayCharacter":"Ketosynthase domain","value":0,"secondaryStructure":"H"},{"description":"","displayCharacter":"Ketosynthase domain","value":0,"secondaryStructure":"H"},{"description":"","displayCharacter":"Ketosynthase domain","value":0,"secondaryStructure":"H"},{"description":"","displayCharacter":"Ketosynthase domain","value":0,"secondaryStructure":"H"},{"description":"","displayCharacter":"Ketosynthase domain","value":0,"secondaryStructure":"H"},{"description":"","displayCharacter":"Ketosynthase domain","value":0,"secondaryStructure":"H"},{"description":"","displayCharacter":"Ketosynthase domain","value":0,"secondaryStructure":"H"},{"description":"","displayCharacter":"Ketosynthase domain","value":0,"secondaryStructure":"H"},{"description":"","displayCharacter":"Ketosynthase domain","value":0,"secondaryStructure":"H"},{"description":"","displayCharacter":"Ketosynthase domain","value":0,"secondaryStructure":"H"},{"description":"","displayCharacter":"Ketosynthase domain","value":0,"secondaryStructure":"H"},{"description":"","displayCharacter":"Ketosynthase domain","value":0,"secondaryStructure":"H"},{"description":"","displayCharacter":"Ketosynthase domain","value":0,"secondaryStructure":"H"},{"description":"","displayCharacter":"Ketosynthase domain","value":0,"secondaryStructure":"H"},{"description":"","displayCharacter":"Ketosynthase domain","value":0,"secondaryStructure":"H"},{"description":"","displayCharacter":"Ketosynthase domain","value":0,"secondaryStructure":"H"},{"description":"","displayCharacter":"Ketosynthase domain","value":0,"secondaryStructure":"H"},{"description":"","displayCharacter":"Ketosynthase domain","value":0,"secondaryStructure":"H"},{"description":"","displayCharacter":"Ketosynthase domain","value":0,"secondaryStructure":"H"},{"description":"","displayCharacter":"Ketosynthase domain","value":0,"secondaryStructure":"H"},{"description":"","displayCharacter":"Ketosynthase domain","value":0,"secondaryStructure":"H"},{"description":"","displayCharacter":"Ketosynthase domain","value":0,"secondaryStructure":"H"},{"description":"","displayCharacter":"Ketosynthase domain","value":0,"secondaryStructure":"H"},{"description":"","displayCharacter":"Ketosynthase domain","value":0,"secondaryStructure":"H"},{"description":"","displayCharacter":"Ketosynthase domain","value":0,"secondaryStructure":"H"},{"description":"","displayCharacter":"Ketosynthase domain","value":0,"secondaryStructure":"H"},{"description":"","displayCharacter":"Ketosynthase domain","value":0,"secondaryStructure":"H"},{"description":"","displayCharacter":"Ketosynthase domain","value":0,"secondaryStructure":"H"},{"description":"","displayCharacter":"Ketosynthase domain","value":0,"secondaryStructure":"H"},{"description":"","displayCharacter":"Ketosynthase domain","value":0,"secondaryStructure":"H"},{"description":"","displayCharacter":"Ketosynthase domain","value":0,"secondaryStructure":"H"},{"description":"","displayCharacter":"Ketosynthase domain","value":0,"secondaryStructure":"H"},{"description":"","displayCharacter":"Ketosynthase domain","value":0,"secondaryStructure":"H"},{"description":"","displayCharacter":"Ketosynthase domain","value":0,"secondaryStructure":"H"},{"description":"","displayCharacter":"Ketosynthase domain","value":0,"secondaryStructure":"H"},{"description":"","displayCharacter":"Ketosynthase domain","value":0,"secondaryStructure":"H"},{"description":"","displayCharacter":"Ketosynthase domain","value":0,"secondaryStructure":"H"},{"description":"","displayCharacter":"Ketosynthase domain","value":0,"secondaryStructure":"H"},{"description":"","displayCharacter":"Ketosynthase domain","value":0,"secondaryStructure":"H"},{"description":"","displayCharacter":"Ketosynthase domain","value":0,"secondaryStructure":"H"},{"description":"","displayCharacter":"Ketosynthase domain","value":0,"secondaryStructure":"H"},{"description":"","displayCharacter":"Ketosynthase domain","value":0,"secondaryStructure":"H"},{"description":"","displayCharacter":"Ketosynthase domain","value":0,"secondaryStructure":"H"},{"description":"","displayCharacter":"Ketosynthase domain","value":0,"secondaryStructure":"H"},{"description":"","displayCharacter":"Ketosynthase domain","value":0,"secondaryStructure":"H"},{"description":"","displayCharacter":"Ketosynthase domain","value":0,"secondaryStructure":"H"},{"description":"","displayCharacter":"Ketosynthase domain","value":0,"secondaryStructure":"H"},{"description":"","displayCharacter":"Ketosynthase domain","value":0,"secondaryStructure":"H"},{"description":"","displayCharacter":"Ketosynthase domain","value":0,"secondaryStructure":"H"},{"description":"","displayCharacter":"Ketosynthase domain","value":0,"secondaryStructure":"H"},{"description":"","displayCharacter":"Ketosynthase domain","value":0,"secondaryStructure":"H"},{"description":"","displayCharacter":"Ketosynthase domain","value":0,"secondaryStructure":"H"},{"description":"","displayCharacter":"Ketosynthase domain","value":0,"secondaryStructure":"H"},{"description":"","displayCharacter":"Ketosynthase domain","value":0,"secondaryStructure":"H"},{"description":"","displayCharacter":"Ketosynthase domain","value":0,"secondaryStructure":"H"},{"description":"","displayCharacter":"Ketosynthase domain","value":0,"secondaryStructure":"H"},{"description":"","displayCharacter":"Ketosynthase domain","value":0,"secondaryStructure":"H"},{"description":"","displayCharacter":"Ketosynthase domain","value":0,"secondaryStructure":"H"},{"description":"","displayCharacter":"Ketosynthase domain","value":0,"secondaryStructure":"H"},{"description":"","displayCharacter":"Ketosynthase domain","value":0,"secondaryStructure":"H"},{"description":"","displayCharacter":"Ketosynthase domain","value":0,"secondaryStructure":"H"},{"description":"","displayCharacter":"Ketosynthase domain","value":0,"secondaryStructure":"H"},{"description":"","displayCharacter":"Ketosynthase domain","value":0,"secondaryStructure":"H"},{"description":"","displayCharacter":"Ketosynthase domain","value":0,"secondaryStructure":"H"},{"description":"","displayCharacter":"Ketosynthase domain","value":0,"secondaryStructure":"H"},{"description":"","displayCharacter":"Ketosynthase domain","value":0,"secondaryStructure":"H"},{"description":"","displayCharacter":"Ketosynthase domain","value":0,"secondaryStructure":"H"},{"description":"","displayCharacter":"Ketosynthase domain","value":0,"secondaryStructure":"H"},{"description":"","displayCharacter":"Ketosynthase domain","value":0,"secondaryStructure":"H"},{"description":"","displayCharacter":"Ketosynthase domain","value":0,"secondaryStructure":"H"},{"description":"","displayCharacter":"Ketosynthase domain","value":0,"secondaryStructure":"H"},{"description":"","displayCharacter":"Ketosynthase domain","value":0,"secondaryStructure":"H"},{"description":"","displayCharacter":"Ketosynthase domain","value":0,"secondaryStructure":"H"},{"description":"","displayCharacter":"Ketosynthase domain","value":0,"secondaryStructure":"H"},{"description":"","displayCharacter":"Ketosynthase domain","value":0,"secondaryStructure":"H"},{"description":"","displayCharacter":"Ketosynthase domain","value":0,"secondaryStructure":"H"},{"description":"","displayCharacter":"Ketosynthase domain","value":0,"secondaryStructure":"H"},{"description":"","displayCharacter":"Ketosynthase domain","value":0,"secondaryStructure":"H"},{"description":"","displayCharacter":"Ketosynthase domain","value":0,"secondaryStructure":"H"},{"description":"","displayCharacter":"Ketosynthase domain","value":0,"secondaryStructure":"H"},{"description":"","displayCharacter":"Ketosynthase domain","value":0,"secondaryStructure":"H"},{"description":"","displayCharacter":"Ketosynthase domain","value":0,"secondaryStructure":"H"},{"description":"","displayCharacter":"Ketosynthase domain","value":0,"secondaryStructure":"H"},{"description":"","displayCharacter":"Ketosynthase domain","value":0,"secondaryStructure":"H"},{"description":"","displayCharacter":"Ketosynthase domain","value":0,"secondaryStructure":"H"},{"description":"","displayCharacter":"Ketosynthase domain","value":0,"secondaryStructure":"H"},{"description":"","displayCharacter":"Ketosynthase domain","value":0,"secondaryStructure":"H"},{"description":"","displayCharacter":"Ketosynthase domain","value":0,"secondaryStructure":"H"},{"description":"","displayCharacter":"Ketosynthase domain","value":0,"secondaryStructure":"H"},{"description":"","displayCharacter":"Ketosynthase domain","value":0,"secondaryStructure":"H"},{"description":"","displayCharacter":"Ketosynthase domain","value":0,"secondaryStructure":"H"},{"description":"","displayCharacter":"Ketosynthase domain","value":0,"secondaryStructure":"H"},{"description":"","displayCharacter":"Ketosynthase domain","value":0,"secondaryStructure":"H"},{"description":"","displayCharacter":"Ketosynthase domain","value":0,"secondaryStructure":"H"},{"description":"","displayCharacter":"Ketosynthase domain","value":0,"secondaryStructure":"H"},{"description":"","displayCharacter":"Ketosynthase domain","value":0,"secondaryStructure":"H"},{"description":"","displayCharacter":"Ketosynthase domain","value":0,"secondaryStructure":"H"},{"description":"","displayCharacter":"Ketosynthase domain","value":0,"secondaryStructure":"H"},{"description":"","displayCharacter":"Ketosynthase domain","value":0,"secondaryStructure":"H"},{"description":"","displayCharacter":"Ketosynthase domain","value":0,"secondaryStructure":"H"},{"description":"","displayCharacter":"Ketosynthase domain","value":0,"secondaryStructure":"H"},{"description":"","displayCharacter":"Ketosynthase domain","value":0,"secondaryStructure":"H"},{"description":"","displayCharacter":"Ketosynthase domain","value":0,"secondaryStructure":"H"},{"description":"","displayCharacter":"Ketosynthase domain","value":0,"secondaryStructure":"H"},{"description":"","displayCharacter":"Ketosynthase domain","value":0,"secondaryStructure":"H"},{"description":"","displayCharacter":"Ketosynthase domain","value":0,"secondaryStructure":"H"},{"description":"","displayCharacter":"Ketosynthase domain","value":0,"secondaryStructure":"H"},{"description":"","displayCharacter":"Ketosynthase domain","value":0,"secondaryStructure":"H"},{"description":"","displayCharacter":"Ketosynthase domain","value":0,"secondaryStructure":"H"},{"description":"","displayCharacter":"Ketosynthase domain","value":0,"secondaryStructure":"H"},{"description":"","displayCharacter":"Ketosynthase domain","value":0,"secondaryStructure":"H"},{"description":"","displayCharacter":"Ketosynthase domain","value":0,"secondaryStructure":"H"},{"description":"","displayCharacter":"Ketosynthase domain","value":0,"secondaryStructure":"H"},{"description":"","displayCharacter":"Ketosynthase domain","value":0,"secondaryStructure":"H"},{"description":"","displayCharacter":"Ketosynthase domain","value":0,"secondaryStructure":"H"},{"description":"","displayCharacter":"Ketosynthase domain","value":0,"secondaryStructure":"H"},{"description":"","displayCharacter":"Ketosynthase domain","value":0,"secondaryStructure":"H"},{"description":"","displayCharacter":"Ketosynthase domain","value":0,"secondaryStructure":"H"},{"description":"","displayCharacter":"Ketosynthase domain","value":0,"secondaryStructure":"H"},{"description":"","displayCharacter":"Ketosynthase domain","value":0,"secondaryStructure":"H"},{"description":"","displayCharacter":"Ketosynthase domain","value":0,"secondaryStructure":"H"},{"description":"","displayCharacter":"Ketosynthase domain","value":0,"secondaryStructure":"H"},{"description":"","displayCharacter":"Ketosynthase domain","value":0,"secondaryStructure":"H"},{"description":"","displayCharacter":"Ketosynthase domain","value":0,"secondaryStructure":"H"},{"description":"","displayCharacter":"Ketosynthase domain","value":0,"secondaryStructure":"H"},{"description":"","displayCharacter":"Ketosynthase domain","value":0,"secondaryStructure":"H"},{"description":"","displayCharacter":"Ketosynthase domain","value":0,"secondaryStructure":"H"},{"description":"","displayCharacter":"Ketosynthase domain","value":0,"secondaryStructure":"H"},{"description":"","displayCharacter":"Ketosynthase domain","value":0,"secondaryStructure":"H"},{"description":"","displayCharacter":"Ketosynthase domain","value":0,"secondaryStructure":"H"},{"description":"","displayCharacter":"Ketosynthase domain","value":0,"secondaryStructure":"H"},{"description":"","displayCharacter":"Ketosynthase domain","value":0,"secondaryStructure":"H"},{"description":"","displayCharacter":"Ketosynthase domain","value":0,"secondaryStructure":"H"},{"description":"","displayCharacter":"Ketosynthase domain","value":0,"secondaryStructure":"H"},{"description":"","displayCharacter":"Ketosynthase domain","value":0,"secondaryStructure":"H"},{"description":"","displayCharacter":"Ketosynthase domain","value":0,"secondaryStructure":"H"},{"description":"","displayCharacter":"Ketosynthase domain","value":0,"secondaryStructure":"H"},{"description":"","displayCharacter":"Ketosynthase domain","value":0,"secondaryStructure":"H"},{"description":"","displayCharacter":"Ketosynthase domain","value":0,"secondaryStructure":"H"},{"description":"","displayCharacter":"Ketosynthase domain","value":0,"secondaryStructure":"H"},{"description":"","displayCharacter":"Ketosynthase domain","value":0,"secondaryStructure":"H"},{"description":"","displayCharacter":"Ketosynthase domain","value":0,"secondaryStructure":"H"},{"description":"","displayCharacter":"Ketosynthase domain","value":0,"secondaryStructure":"H"},{"description":"","displayCharacter":"Ketosynthase domain","value":0,"secondaryStructure":"H"},{"description":"","displayCharacter":"Ketosynthase domain","value":0,"secondaryStructure":"H"},{"description":"","displayCharacter":"Ketosynthase domain","value":0,"secondaryStructure":"H"},{"description":"","displayCharacter":"Ketosynthase domain","value":0,"secondaryStructure":"H"},{"description":"","displayCharacter":"Ketosynthase domain","value":0,"secondaryStructure":"H"},{"description":"","displayCharacter":"Ketosynthase domain","value":0,"secondaryStructure":"H"},{"description":"","displayCharacter":"Ketosynthase domain","value":0,"secondaryStructure":"H"},{"description":"","displayCharacter":"Ketosynthase domain","value":0,"secondaryStructure":"H"},{"description":"","displayCharacter":"Ketosynthase domain","value":0,"secondaryStructure":"H"},{"description":"","displayCharacter":"Ketosynthase domain","value":0,"secondaryStructure":"H"},{"description":"","displayCharacter":"Ketosynthase domain","value":0,"secondaryStructure":"H"},{"description":"","displayCharacter":"Ketosynthase domain","value":0,"secondaryStructure":"H"},{"description":"","displayCharacter":"Ketosynthase domain","value":0,"secondaryStructure":"H"},{"description":"","displayCharacter":"Ketosynthase domain","value":0,"secondaryStructure":"H"},{"description":"","displayCharacter":"Ketosynthase domain","value":0,"secondaryStructure":"H"},{"description":"","displayCharacter":"Ketosynthase domain","value":0,"secondaryStructure":"H"},{"description":"","displayCharacter":"Ketosynthase domain","value":0,"secondaryStructure":"H"},{"description":"","displayCharacter":"Ketosynthase domain","value":0,"secondaryStructure":"H"},{"description":"","displayCharacter":"Ketosynthase domain","value":0,"secondaryStructure":"H"},{"description":"","displayCharacter":"Ketosynthase domain","value":0,"secondaryStructure":"H"},{"description":"","displayCharacter":"Ketosynthase domain","value":0,"secondaryStructure":"H"},{"description":"","displayCharacter":"Ketosynthase domain","value":0,"secondaryStructure":"H"},{"description":"","displayCharacter":"Ketosynthase domain","value":0,"secondaryStructure":"H"},{"description":"","displayCharacter":"Ketosynthase domain","value":0,"secondaryStructure":"H"},{"description":"","displayCharacter":"Ketosynthase domain","value":0,"secondaryStructure":"H"},{"description":"","displayCharacter":"Ketosynthase domain","value":0,"secondaryStructure":"H"},{"description":"","displayCharacter":"Ketosynthase domain","value":0,"secondaryStructure":"H"},{"description":"","displayCharacter":"Ketosynthase domain","value":0,"secondaryStructure":"H"},{"description":"","displayCharacter":"Ketosynthase domain","value":0,"secondaryStructure":"H"},{"description":"","displayCharacter":"Ketosynthase domain","value":0,"secondaryStructure":"H"},{"description":"","displayCharacter":"Ketosynthase domain","value":0,"secondaryStructure":"H"},{"description":"","displayCharacter":"Ketosynthase domain","value":0,"secondaryStructure":"H"},{"description":"","displayCharacter":"Ketosynthase domain","value":0,"secondaryStructure":"H"},{"description":"","displayCharacter":"Ketosynthase domain","value":0,"secondaryStructure":"H"},{"description":"","displayCharacter":"Ketosynthase domain","value":0,"secondaryStructure":"H"},{"description":"","displayCharacter":"Ketosynthase domain","value":0,"secondaryStructure":"H"},{"description":"","displayCharacter":"Ketosynthase domain","value":0,"secondaryStructure":"H"},{"description":"","displayCharacter":"Ketosynthase domain","value":0,"secondaryStructure":"H"},{"description":"","displayCharacter":"Ketosynthase domain","value":0,"secondaryStructure":"H"},{"description":"","displayCharacter":"Ketosynthase domain","value":0,"secondaryStructure":"H"},{"description":"","displayCharacter":"Ketosynthase domain","value":0,"secondaryStructure":"H"},{"description":"","displayCharacter":"Ketosynthase domain","value":0,"secondaryStructure":"H"},{"description":"","displayCharacter":"Ketosynthase domain","value":0,"secondaryStructure":"H"},{"description":"","displayCharacter":"Ketosynthase domain","value":0,"secondaryStructure":"H"},{"description":"","displayCharacter":"Ketosynthase domain","value":0,"secondaryStructure":"H"},{"description":"","displayCharacter":"Ketosynthase domain","value":0,"secondaryStructure":"H"},{"description":"","displayCharacter":"Ketosynthase domain","value":0,"secondaryStructure":"H"},{"description":"","displayCharacter":"Ketosynthase domain","value":0,"secondaryStructure":"H"},{"description":"","displayCharacter":"Ketosynthase domain","value":0,"secondaryStructure":"H"},{"description":"","displayCharacter":"Ketosynthase domain","value":0,"secondaryStructure":"H"},{"description":"","displayCharacter":"Ketosynthase domain","value":0,"secondaryStructure":"H"},{"description":"","displayCharacter":"Ketosynthase domain","value":0,"secondaryStructure":"H"},{"description":"","displayCharacter":"Ketosynthase domain","value":0,"secondaryStructure":"H"},{"description":"","displayCharacter":"Ketosynthase domain","value":0,"secondaryStructure":"H"},{"description":"","displayCharacter":"Ketosynthase domain","value":0,"secondaryStructure":"H"},{"description":"","displayCharacter":"Ketosynthase domain","value":0,"secondaryStructure":"H"},{"description":"","displayCharacter":"Ketosynthase domain","value":0,"secondaryStructure":"H"},{"description":"","displayCharacter":"Ketosynthase domain","value":0,"secondaryStructure":"H"},{"description":"","displayCharacter":"Ketosynthase domain","value":0,"secondaryStructure":"H"},{"description":"","displayCharacter":"Ketosynthase domain","value":0,"secondaryStructure":"H"},{"description":"","displayCharacter":"Ketosynthase domain","value":0,"secondaryStructure":"H"},{"description":"","displayCharacter":"Ketosynthase domain","value":0,"secondaryStructure":"H"},{"description":"","displayCharacter":"Ketosynthase domain","value":0,"secondaryStructure":"H"},{"description":"","displayCharacter":"Ketosynthase domain","value":0,"secondaryStructure":"H"},{"description":"","displayCharacter":"Ketosynthase domain","value":0,"secondaryStructure":"H"},{"description":"","displayCharacter":"Ketosynthase domain","value":0,"secondaryStructure":"H"},{"description":"","displayCharacter":"Ketosynthase domain","value":0,"secondaryStructure":"H"},{"description":"","displayCharacter":"Ketosynthase domain","value":0,"secondaryStructure":"H"},{"description":"","displayCharacter":"Ketosynthase domain","value":0,"secondaryStructure":"H"},{"description":"","displayCharacter":"Ketosynthase domain","value":0,"secondaryStructure":"H"},{"description":"","displayCharacter":"Ketosynthase domain","value":0,"secondaryStructure":"H"},{"description":"","displayCharacter":"Ketosynthase domain","value":0,"secondaryStructure":"H"},{"description":"","displayCharacter":"Ketosynthase domain","value":0,"secondaryStructure":"H"},{"description":"","displayCharacter":"Ketosynthase domain","value":0,"secondaryStructure":"H"},{"description":"","displayCharacter":"Ketosynthase domain","value":0,"secondaryStructure":"H"},{"description":"","displayCharacter":"Ketosynthase domain","value":0,"secondaryStructure":"H"},{"description":"","displayCharacter":"Ketosynthase domain","value":0,"secondaryStructure":"H"},{"description":"","displayCharacter":"Ketosynthase domain","value":0,"secondaryStructure":"H"},{"description":"","displayCharacter":"Ketosynthase domain","value":0,"secondaryStructure":"H"},{"description":"","displayCharacter":"Ketosynthase domain","value":0,"secondaryStructure":"H"},{"description":"","displayCharacter":"Ketosynthase domain","value":0,"secondaryStructure":"H"},{"description":"","displayCharacter":"Ketosynthase domain","value":0,"secondaryStructure":"H"},{"description":"","displayCharacter":"Ketosynthase domain","value":0,"secondaryStructure":"H"},{"description":"","displayCharacter":"Ketosynthase domain","value":0,"secondaryStructure":"H"},{"description":"","displayCharacter":"Ketosynthase domain","value":0,"secondaryStructure":"H"},{"description":"","displayCharacter":"Ketosynthase domain","value":0,"secondaryStructure":"H"},{"description":"","displayCharacter":"Ketosynthase domain","value":0,"secondaryStructure":"H"},{"description":"","displayCharacter":"Ketosynthase domain","value":0,"secondaryStructure":"H"},{"description":"","displayCharacter":"Ketosynthase domain","value":0,"secondaryStructure":"H"},{"description":"","displayCharacter":"Ketosynthase domain","value":0,"secondaryStructure":"H"},{"description":"","displayCharacter":"Ketosynthase domain","value":0,"secondaryStructure":"H"},{"description":"","displayCharacter":"Ketosynthase domain","value":0,"secondaryStructure":"H"},{"description":"","displayCharacter":"Ketosynthase domain","value":0,"secondaryStructure":"H"},{"description":"","displayCharacter":"Ketosynthase domain","value":0,"secondaryStructure":"H"},{"description":"","displayCharacter":"Ketosynthase domain","value":0,"secondaryStructure":"H"},{"description":"","displayCharacter":"Ketosynthase domain","value":0,"secondaryStructure":"H"},{"description":"","displayCharacter":"Ketosynthase domain","value":0,"secondaryStructure":"H"},{"description":"","displayCharacter":"Ketosynthase domain","value":0,"secondaryStructure":"H"},{"description":"","displayCharacter":"Ketosynthase domain","value":0,"secondaryStructure":"H"},{"description":"","displayCharacter":"Ketosynthase domain","value":0,"secondaryStructure":"H"},{"description":"","displayCharacter":"Ketosynthase domain","value":0,"secondaryStructure":"H"},{"description":"","displayCharacter":"Ketosynthase domain","value":0,"secondaryStructure":"H"},{"description":"","displayCharacter":"Ketosynthase domain","value":0,"secondaryStructure":"H"},{"description":"","displayCharacter":"Ketosynthase domain","value":0,"secondaryStructure":"H"},{"description":"","displayCharacter":"Ketosynthase domain","value":0,"secondaryStructure":"H"},{"description":"","displayCharacter":"Ketosynthase domain","value":0,"secondaryStructure":"H"},{"description":"","displayCharacter":"Ketosynthase domain","value":0,"secondaryStructure":"H"},{"description":"","displayCharacter":"Ketosynthase domain","value":0,"secondaryStructure":"H"},{"description":"","displayCharacter":"Ketosynthase domain","value":0,"secondaryStructure":"H"},{"description":"","displayCharacter":"Ketosynthase domain","value":0,"secondaryStructure":"H"},{"description":"","displayCharacter":"Ketosynthase domain","value":0,"secondaryStructure":"H"},{"description":"","displayCharacter":"Ketosynthase domain","value":0,"secondaryStructure":"H"},{"description":"","displayCharacter":"Ketosynthase domain","value":0,"secondaryStructure":"H"},{"description":"","displayCharacter":"Ketosynthase domain","value":0,"secondaryStructure":"H"},{"description":"","displayCharacter":"Ketosynthase domain","value":0,"secondaryStructure":"H"},{"description":"","displayCharacter":"Ketosynthase domain","value":0,"secondaryStructure":"H"},{"description":"","displayCharacter":"Ketosynthase domain","value":0,"secondaryStructure":"H"},{"description":"","displayCharacter":"Ketosynthase domain","value":0,"secondaryStructure":"H"},{"description":"","displayCharacter":"Ketosynthase domain","value":0,"secondaryStructure":"H"},{"description":"","displayCharacter":"Ketosynthase domain","value":0,"secondaryStructure":"H"},{"description":"","displayCharacter":"Ketosynthase domain","value":0,"secondaryStructure":"H"},{"description":"","displayCharacter":"Ketosynthase domain","value":0,"secondaryStructure":"H"},{"description":"","displayCharacter":"Ketosynthase domain","value":0,"secondaryStructure":"H"},{"description":"","displayCharacter":"Ketosynthase domain","value":0,"secondaryStructure":"H"},{"description":"","displayCharacter":"Ketosynthase domain","value":0,"secondaryStructure":"H"},{"description":"","displayCharacter":"Ketosynthase domain","value":0,"secondaryStructure":"H"},{"description":"","displayCharacter":"Ketosynthase domain","value":0,"secondaryStructure":"H"},{"description":"","displayCharacter":"Ketosynthase domain","value":0,"secondaryStructure":"H"},{"description":"","displayCharacter":"Ketosynthase domain","value":0,"secondaryStructure":"H"},{"description":"","displayCharacter":"Ketosynthase domain","value":0,"secondaryStructure":"H"},{"description":"","displayCharacter":"Ketosynthase domain","value":0,"secondaryStructure":"H"},{"description":"","displayCharacter":"Ketosynthase domain","value":0,"secondaryStructure":"H"},{"description":"","displayCharacter":"Ketosynthase domain","value":0,"secondaryStructure":"H"},{"description":"","displayCharacter":"Ketosynthase domain","value":0,"secondaryStructure":"H"},{"description":"","displayCharacter":"Ketosynthase domain","value":0,"secondaryStructure":"H"},{"description":"","displayCharacter":"Ketosynthase domain","value":0,"secondaryStructure":"H"},{"description":"","displayCharacter":"Ketosynthase domain","value":0,"secondaryStructure":"H"},{"description":"","displayCharacter":"Ketosynthase domain","value":0,"secondaryStructure":"H"},{"description":"","displayCharacter":"Ketosynthase domain","value":0,"secondaryStructure":"H"},{"description":"","displayCharacter":"Ketosynthase domain","value":0,"secondaryStructure":"H"},{"description":"","displayCharacter":"Ketosynthase domain","value":0,"secondaryStructure":"H"},{"description":"","displayCharacter":"Ketosynthase domain","value":0,"secondaryStructure":"H"},{"description":"","displayCharacter":"Ketosynthase domain","value":0,"secondaryStructure":"H"},{"description":"","displayCharacter":"Ketosynthase domain","value":0,"secondaryStructure":"H"},{"description":"","displayCharacter":"Ketosynthase domain","value":0,"secondaryStructure":"H"},{"description":"","displayCharacter":"Ketosynthase domain","value":0,"secondaryStructure":"H"},{"description":"","displayCharacter":"Ketosynthase domain","value":0,"secondaryStructure":"H"},{"description":"","displayCharacter":"Ketosynthase domain","value":0,"secondaryStructure":"H"},{"description":"","displayCharacter":"Ketosynthase domain","value":0,"secondaryStructure":"H"},{"description":"","displayCharacter":"Ketosynthase domain","value":0,"secondaryStructure":"H"},{"description":"","displayCharacter":"Ketosynthase domain","value":0,"secondaryStructure":"H"},{"description":"","displayCharacter":"Ketosynthase domain","value":0,"secondaryStructure":"H"},{"description":"","displayCharacter":"Ketosynthase domain","value":0,"secondaryStructure":"H"},{"description":"","displayCharacter":"Ketosynthase domain","value":0,"secondaryStructure":"H"},{"description":"","displayCharacter":"Ketosynthase domain","value":0,"secondaryStructure":"H"},{"description":"","displayCharacter":"Ketosynthase domain","value":0,"secondaryStructure":"H"},{"description":"","displayCharacter":"Ketosynthase domain","value":0,"secondaryStructure":"H"},{"description":"","displayCharacter":"Ketosynthase domain","value":0,"secondaryStructure":"H"},{"description":"","displayCharacter":"Ketosynthase domain","value":0,"secondaryStructure":"H"},{"description":"","displayCharacter":"Ketosynthase domain","value":0,"secondaryStructure":"H"},{"description":"","displayCharacter":"Ketosynthase domain","value":0,"secondaryStructure":"H"},{"description":"","displayCharacter":"Ketosynthase domain","value":0,"secondaryStructure":"H"},{"description":"","displayCharacter":"Ketosynthase domain","value":0,"secondaryStructure":"H"},{"description":"","displayCharacter":"Ketosynthase domain","value":0,"secondaryStructure":"H"},{"description":"","displayCharacter":"Ketosynthase domain","value":0,"secondaryStructure":"H"},{"description":"","displayCharacter":"Ketosynthase domain","value":0,"secondaryStructure":"H"},{"description":"","displayCharacter":"Ketosynthase domain","value":0,"secondaryStructure":"H"},{"description":"","displayCharacter":"Ketosynthase domain","value":0,"secondaryStructure":"H"},{"description":"","displayCharacter":"Ketosynthase domain","value":0,"secondaryStructure":"H"},{"description":"","displayCharacter":"Ketosynthase domain","value":0,"secondaryStructure":"H"},{"description":"","displayCharacter":"Ketosynthase domain","value":0,"secondaryStructure":"H"},{"description":"","displayCharacter":"Ketosynthase domain","value":0,"secondaryStructure":"H"},{"description":"","displayCharacter":"Ketosynthase domain","value":0,"secondaryStructure":"H"},{"description":"","displayCharacter":"Ketosynthase domain","value":0,"secondaryStructure":"H"},{"description":"","displayCharacter":"Ketosynthase domain","value":0,"secondaryStructure":"H"},{"description":"","displayCharacter":"Ketosynthase domain","value":0,"secondaryStructure":"H"},{"description":"","displayCharacter":"Ketosynthase domain","value":0,"secondaryStructure":"H"},{"description":"","displayCharacter":"Ketosynthase domain","value":0,"secondaryStructure":"H"},{"description":"","displayCharacter":"Ketosynthase domain","value":0,"secondaryStructure":"H"},{"description":"","displayCharacter":"Ketosynthase domain","value":0,"secondaryStructure":"H"},{"description":"","displayCharacter":"Ketosynthase domain","value":0,"secondaryStructure":"H"},{"description":"","displayCharacter":"Ketosynthase domain","value":0,"secondaryStructure":"H"},{"description":"","displayCharacter":"Ketosynthase domain","value":0,"secondaryStructure":"H"},{"description":"","displayCharacter":"Ketosynthase domain","value":0,"secondaryStructure":"H"},{"description":"","displayCharacter":"Ketosynthase domain","value":0,"secondaryStructure":"H"},{"description":"","displayCharacter":"Ketosynthase domain","value":0,"secondaryStructure":"H"},{"description":"","displayCharacter":"Ketosynthase domain","value":0,"secondaryStructure":"H"},{"description":"","displayCharacter":"Ketosynthase domain","value":0,"secondaryStructure":"H"},{"description":"","displayCharacter":"Ketosynthase domain","value":0,"secondaryStructure":"H"},{"description":"","displayCharacter":"Ketosynthase domain","value":0,"secondaryStructure":"H"},{"description":"","displayCharacter":"Ketosynthase domain","value":0,"secondaryStructure":"H"},{"description":"","displayCharacter":"Ketosynthase domain","value":0,"secondaryStructure":"H"},{"description":"","displayCharacter":"Ketosynthase domain","value":0,"secondaryStructure":"H"},{"description":"","displayCharacter":"Ketosynthase domain","value":0,"secondaryStructure":"H"},{"description":"","displayCharacter":"Ketosynthase domain","value":0,"secondaryStructure":"H"},{"description":"","displayCharacter":"Ketosynthase domain","value":0,"secondaryStructure":"H"},{"description":"","displayCharacter":"Ketosynthase domain","value":0,"secondaryStructure":"H"},{"description":"","displayCharacter":"Ketosynthase domain","value":0,"secondaryStructure":"H"},{"description":"","displayCharacter":"Ketosynthase domain","value":0,"secondaryStructure":"H"},{"description":"","displayCharacter":"Ketosynthase domain","value":0,"secondaryStructure":"H"},{"description":"","displayCharacter":"Ketosynthase domain","value":0,"secondaryStructure":"H"},{"description":"","displayCharacter":"Ketosynthase domain","value":0,"secondaryStructure":"H"},{"description":"","displayCharacter":"Ketosynthase domain","value":0,"secondaryStructure":"H"},{"description":"","displayCharacter":"Ketosynthase domain","value":0,"secondaryStructure":"H"},{"description":"","displayCharacter":"Ketosynthase domain","value":0,"secondaryStructure":"H"},{"description":"","displayCharacter":"Ketosynthase domain","value":0,"secondaryStructure":"H"},{"description":"","displayCharacter":"Ketosynthase domain","value":0,"secondaryStructure":"H"},{"description":"","displayCharacter":"Ketosynthase domain","value":0,"secondaryStructure":"H"},{"description":"","displayCharacter":"Ketosynthase domain","value":0,"secondaryStructure":"H"},{"description":"","displayCharacter":"Ketosynthase domain","value":0,"secondaryStructure":"H"},{"description":"","displayCharacter":"Ketosynthase domain","value":0,"secondaryStructure":"H"},{"description":"","displayCharacter":"Ketosynthase domain","value":0,"secondaryStructure":"H"},{"description":"","displayCharacter":"Ketosynthase domain","value":0,"secondaryStructure":"H"},{"description":"","displayCharacter":"Ketosynthase domain","value":0,"secondaryStructure":"H"},{"description":"","displayCharacter":"Ketosynthase domain","value":0,"secondaryStructure":"H"},{"description":"","displayCharacter":"Ketosynthase domain","value":0,"secondaryStructure":"H"},{"description":"","displayCharacter":"Ketosynthase domain","value":0,"secondaryStructure":"H"},{"description":"","displayCharacter":"Ketosynthase domain","value":0,"secondaryStructure":"H"},{"description":"","displayCharacter":"Ketosynthase domain","value":0,"secondaryStructure":"H"},{"value":0,"secondaryStructure":"\u0000"},{"value":0,"secondaryStructure":"\u0000"},{"value":0,"secondaryStructure":"\u0000"},{"value":0,"secondaryStructure":"\u0000"},{"value":0,"secondaryStructure":"\u0000"},{"value":0,"secondaryStructure":"\u0000"},{"value":0,"secondaryStructure":"\u0000"},{"value":0,"secondaryStructure":"\u0000"},{"value":0,"secondaryStructure":"\u0000"},{"value":0,"secondaryStructure":"\u0000"},{"value":0,"secondaryStructure":"\u0000"},{"value":0,"secondaryStructure":"\u0000"},{"value":0,"secondaryStructure":"\u0000"},{"value":0,"secondaryStructure":"\u0000"},{"description":"","displayCharacter":"Acyltransferase domain","value":0,"secondaryStructure":"H"},{"description":"","displayCharacter":"Acyltransferase domain","value":0,"secondaryStructure":"H"},{"description":"","displayCharacter":"Acyltransferase domain","value":0,"secondaryStructure":"H"},{"description":"","displayCharacter":"Acyltransferase domain","value":0,"secondaryStructure":"H"},{"description":"","displayCharacter":"Acyltransferase domain","value":0,"secondaryStructure":"H"},{"description":"","displayCharacter":"Acyltransferase domain","value":0,"secondaryStructure":"H"},{"description":"","displayCharacter":"Acyltransferase domain","value":0,"secondaryStructure":"H"},{"description":"","displayCharacter":"Acyltransferase domain","value":0,"secondaryStructure":"H"},{"description":"","displayCharacter":"Acyltransferase domain","value":0,"secondaryStructure":"H"},{"description":"","displayCharacter":"Acyltransferase domain","value":0,"secondaryStructure":"H"},{"description":"","displayCharacter":"Acyltransferase domain","value":0,"secondaryStructure":"H"},{"description":"","displayCharacter":"Acyltransferase domain","value":0,"secondaryStructure":"H"},{"description":"","displayCharacter":"Acyltransferase domain","value":0,"secondaryStructure":"H"},{"description":"","displayCharacter":"Acyltransferase domain","value":0,"secondaryStructure":"H"},{"description":"","displayCharacter":"Acyltransferase domain","value":0,"secondaryStructure":"H"},{"description":"","displayCharacter":"Acyltransferase domain","value":0,"secondaryStructure":"H"},{"description":"","displayCharacter":"Acyltransferase domain","value":0,"secondaryStructure":"H"},{"description":"","displayCharacter":"Acyltransferase domain","value":0,"secondaryStructure":"H"},{"description":"","displayCharacter":"Acyltransferase domain","value":0,"secondaryStructure":"H"},{"description":"","displayCharacter":"Acyltransferase domain","value":0,"secondaryStructure":"H"},{"description":"","displayCharacter":"Acyltransferase domain","value":0,"secondaryStructure":"H"},{"description":"","displayCharacter":"Acyltransferase domain","value":0,"secondaryStructure":"H"},{"description":"","displayCharacter":"Acyltransferase domain","value":0,"secondaryStructure":"H"},{"description":"","displayCharacter":"Acyltransferase domain","value":0,"secondaryStructure":"H"},{"description":"","displayCharacter":"Acyltransferase domain","value":0,"secondaryStructure":"H"},{"description":"","displayCharacter":"Acyltransferase domain","value":0,"secondaryStructure":"H"},{"description":"","displayCharacter":"Acyltransferase domain","value":0,"secondaryStructure":"H"},{"description":"","displayCharacter":"Acyltransferase domain","value":0,"secondaryStructure":"H"},{"description":"","displayCharacter":"Acyltransferase domain","value":0,"secondaryStructure":"H"},{"description":"","displayCharacter":"Acyltransferase domain","value":0,"secondaryStructure":"H"},{"description":"","displayCharacter":"Acyltransferase domain","value":0,"secondaryStructure":"H"},{"description":"","displayCharacter":"Acyltransferase domain","value":0,"secondaryStructure":"H"},{"description":"","displayCharacter":"Acyltransferase domain","value":0,"secondaryStructure":"H"},{"description":"","displayCharacter":"Acyltransferase domain","value":0,"secondaryStructure":"H"},{"description":"","displayCharacter":"Acyltransferase domain","value":0,"secondaryStructure":"H"},{"description":"","displayCharacter":"Acyltransferase domain","value":0,"secondaryStructure":"H"},{"description":"","displayCharacter":"Acyltransferase domain","value":0,"secondaryStructure":"H"},{"description":"","displayCharacter":"Acyltransferase domain","value":0,"secondaryStructure":"H"},{"description":"","displayCharacter":"Acyltransferase domain","value":0,"secondaryStructure":"H"},{"description":"","displayCharacter":"Acyltransferase domain","value":0,"secondaryStructure":"H"},{"description":"","displayCharacter":"Acyltransferase domain","value":0,"secondaryStructure":"H"},{"description":"","displayCharacter":"Acyltransferase domain","value":0,"secondaryStructure":"H"},{"description":"","displayCharacter":"Acyltransferase domain","value":0,"secondaryStructure":"H"},{"description":"","displayCharacter":"Acyltransferase domain","value":0,"secondaryStructure":"H"},{"description":"","displayCharacter":"Acyltransferase domain","value":0,"secondaryStructure":"H"},{"description":"","displayCharacter":"Acyltransferase domain","value":0,"secondaryStructure":"H"},{"description":"","displayCharacter":"Acyltransferase domain","value":0,"secondaryStructure":"H"},{"description":"","displayCharacter":"Acyltransferase domain","value":0,"secondaryStructure":"H"},{"description":"","displayCharacter":"Acyltransferase domain","value":0,"secondaryStructure":"H"},{"description":"","displayCharacter":"Acyltransferase domain","value":0,"secondaryStructure":"H"},{"description":"","displayCharacter":"Acyltransferase domain","value":0,"secondaryStructure":"H"},{"description":"","displayCharacter":"Acyltransferase domain","value":0,"secondaryStructure":"H"},{"description":"","displayCharacter":"Acyltransferase domain","value":0,"secondaryStructure":"H"},{"description":"","displayCharacter":"Acyltransferase domain","value":0,"secondaryStructure":"H"},{"description":"","displayCharacter":"Acyltransferase domain","value":0,"secondaryStructure":"H"},{"description":"","displayCharacter":"Acyltransferase domain","value":0,"secondaryStructure":"H"},{"description":"","displayCharacter":"Acyltransferase domain","value":0,"secondaryStructure":"H"},{"description":"","displayCharacter":"Acyltransferase domain","value":0,"secondaryStructure":"H"},{"description":"","displayCharacter":"Acyltransferase domain","value":0,"secondaryStructure":"H"},{"description":"","displayCharacter":"Acyltransferase domain","value":0,"secondaryStructure":"H"},{"description":"","displayCharacter":"Acyltransferase domain","value":0,"secondaryStructure":"H"},{"description":"","displayCharacter":"Acyltransferase domain","value":0,"secondaryStructure":"H"},{"description":"","displayCharacter":"Acyltransferase domain","value":0,"secondaryStructure":"H"},{"description":"","displayCharacter":"Acyltransferase domain","value":0,"secondaryStructure":"H"},{"description":"","displayCharacter":"Acyltransferase domain","value":0,"secondaryStructure":"H"},{"description":"","displayCharacter":"Acyltransferase domain","value":0,"secondaryStructure":"H"},{"description":"","displayCharacter":"Acyltransferase domain","value":0,"secondaryStructure":"H"},{"description":"","displayCharacter":"Acyltransferase domain","value":0,"secondaryStructure":"H"},{"description":"","displayCharacter":"Acyltransferase domain","value":0,"secondaryStructure":"H"},{"description":"","displayCharacter":"Acyltransferase domain","value":0,"secondaryStructure":"H"},{"description":"","displayCharacter":"Acyltransferase domain","value":0,"secondaryStructure":"H"},{"description":"","displayCharacter":"Acyltransferase domain","value":0,"secondaryStructure":"H"},{"description":"","displayCharacter":"Acyltransferase domain","value":0,"secondaryStructure":"H"},{"description":"","displayCharacter":"Acyltransferase domain","value":0,"secondaryStructure":"H"},{"description":"","displayCharacter":"Acyltransferase domain","value":0,"secondaryStructure":"H"},{"description":"","displayCharacter":"Acyltransferase domain","value":0,"secondaryStructure":"H"},{"description":"","displayCharacter":"Acyltransferase domain","value":0,"secondaryStructure":"H"},{"description":"","displayCharacter":"Acyltransferase domain","value":0,"secondaryStructure":"H"},{"description":"","displayCharacter":"Acyltransferase domain","value":0,"secondaryStructure":"H"},{"description":"","displayCharacter":"Acyltransferase domain","value":0,"secondaryStructure":"H"},{"description":"","displayCharacter":"Acyltransferase domain","value":0,"secondaryStructure":"H"},{"description":"","displayCharacter":"Acyltransferase domain","value":0,"secondaryStructure":"H"},{"description":"","displayCharacter":"Acyltransferase domain","value":0,"secondaryStructure":"H"},{"description":"","displayCharacter":"Acyltransferase domain","value":0,"secondaryStructure":"H"},{"description":"","displayCharacter":"Acyltransferase domain","value":0,"secondaryStructure":"H"},{"description":"","displayCharacter":"Acyltransferase domain","value":0,"secondaryStructure":"H"},{"description":"","displayCharacter":"Acyltransferase domain","value":0,"secondaryStructure":"H"},{"description":"","displayCharacter":"Acyltransferase domain","value":0,"secondaryStructure":"H"},{"description":"","displayCharacter":"Acyltransferase domain","value":0,"secondaryStructure":"H"},{"description":"","displayCharacter":"Acyltransferase domain","value":0,"secondaryStructure":"H"},{"description":"","displayCharacter":"Acyltransferase domain","value":0,"secondaryStructure":"H"},{"description":"","displayCharacter":"Acyltransferase domain","value":0,"secondaryStructure":"H"},{"description":"","displayCharacter":"Acyltransferase domain","value":0,"secondaryStructure":"H"},{"description":"","displayCharacter":"Acyltransferase domain","value":0,"secondaryStructure":"H"},{"description":"","displayCharacter":"Acyltransferase domain","value":0,"secondaryStructure":"H"},{"description":"","displayCharacter":"Acyltransferase domain","value":0,"secondaryStructure":"H"},{"description":"","displayCharacter":"Acyltransferase domain","value":0,"secondaryStructure":"H"},{"description":"","displayCharacter":"Acyltransferase domain","value":0,"secondaryStructure":"H"},{"description":"","displayCharacter":"Acyltransferase domain","value":0,"secondaryStructure":"H"},{"description":"","displayCharacter":"Acyltransferase domain","value":0,"secondaryStructure":"H"},{"description":"","displayCharacter":"Acyltransferase domain","value":0,"secondaryStructure":"H"},{"description":"","displayCharacter":"Acyltransferase domain","value":0,"secondaryStructure":"H"},{"description":"","displayCharacter":"Acyltransferase domain","value":0,"secondaryStructure":"H"},{"description":"","displayCharacter":"Acyltransferase domain","value":0,"secondaryStructure":"H"},{"description":"","displayCharacter":"Acyltransferase domain","value":0,"secondaryStructure":"H"},{"description":"","displayCharacter":"Acyltransferase domain","value":0,"secondaryStructure":"H"},{"description":"","displayCharacter":"Acyltransferase domain","value":0,"secondaryStructure":"H"},{"description":"","displayCharacter":"Acyltransferase domain","value":0,"secondaryStructure":"H"},{"description":"","displayCharacter":"Acyltransferase domain","value":0,"secondaryStructure":"H"},{"description":"","displayCharacter":"Acyltransferase domain","value":0,"secondaryStructure":"H"},{"description":"","displayCharacter":"Acyltransferase domain","value":0,"secondaryStructure":"H"},{"description":"","displayCharacter":"Acyltransferase domain","value":0,"secondaryStructure":"H"},{"description":"","displayCharacter":"Acyltransferase domain","value":0,"secondaryStructure":"H"},{"description":"","displayCharacter":"Acyltransferase domain","value":0,"secondaryStructure":"H"},{"description":"","displayCharacter":"Acyltransferase domain","value":0,"secondaryStructure":"H"},{"description":"","displayCharacter":"Acyltransferase domain","value":0,"secondaryStructure":"H"},{"description":"","displayCharacter":"Acyltransferase domain","value":0,"secondaryStructure":"H"},{"description":"","displayCharacter":"Acyltransferase domain","value":0,"secondaryStructure":"H"},{"description":"","displayCharacter":"Acyltransferase domain","value":0,"secondaryStructure":"H"},{"description":"","displayCharacter":"Acyltransferase domain","value":0,"secondaryStructure":"H"},{"description":"","displayCharacter":"Acyltransferase domain","value":0,"secondaryStructure":"H"},{"description":"","displayCharacter":"Acyltransferase domain","value":0,"secondaryStructure":"H"},{"description":"","displayCharacter":"Acyltransferase domain","value":0,"secondaryStructure":"H"},{"description":"","displayCharacter":"Acyltransferase domain","value":0,"secondaryStructure":"H"},{"description":"","displayCharacter":"Acyltransferase domain","value":0,"secondaryStructure":"H"},{"description":"","displayCharacter":"Acyltransferase domain","value":0,"secondaryStructure":"H"},{"description":"","displayCharacter":"Acyltransferase domain","value":0,"secondaryStructure":"H"},{"description":"","displayCharacter":"Acyltransferase domain","value":0,"secondaryStructure":"H"},{"description":"","displayCharacter":"Acyltransferase domain","value":0,"secondaryStructure":"H"},{"description":"","displayCharacter":"Acyltransferase domain","value":0,"secondaryStructure":"H"},{"description":"","displayCharacter":"Acyltransferase domain","value":0,"secondaryStructure":"H"},{"description":"","displayCharacter":"Acyltransferase domain","value":0,"secondaryStructure":"H"},{"description":"","displayCharacter":"Acyltransferase domain","value":0,"secondaryStructure":"H"},{"description":"","displayCharacter":"Acyltransferase domain","value":0,"secondaryStructure":"H"},{"description":"","displayCharacter":"Acyltransferase domain","value":0,"secondaryStructure":"H"},{"description":"","displayCharacter":"Acyltransferase domain","value":0,"secondaryStructure":"H"},{"description":"","displayCharacter":"Acyltransferase domain","value":0,"secondaryStructure":"H"},{"description":"","displayCharacter":"Acyltransferase domain","value":0,"secondaryStructure":"H"},{"description":"","displayCharacter":"Acyltransferase domain","value":0,"secondaryStructure":"H"},{"description":"","displayCharacter":"Acyltransferase domain","value":0,"secondaryStructure":"H"},{"description":"","displayCharacter":"Acyltransferase domain","value":0,"secondaryStructure":"H"},{"description":"","displayCharacter":"Acyltransferase domain","value":0,"secondaryStructure":"H"},{"description":"","displayCharacter":"Acyltransferase domain","value":0,"secondaryStructure":"H"},{"description":"","displayCharacter":"Acyltransferase domain","value":0,"secondaryStructure":"H"},{"description":"","displayCharacter":"Acyltransferase domain","value":0,"secondaryStructure":"H"},{"description":"","displayCharacter":"Acyltransferase domain","value":0,"secondaryStructure":"H"},{"description":"","displayCharacter":"Acyltransferase domain","value":0,"secondaryStructure":"H"},{"description":"","displayCharacter":"Acyltransferase domain","value":0,"secondaryStructure":"H"},{"description":"","displayCharacter":"Acyltransferase domain","value":0,"secondaryStructure":"H"},{"description":"","displayCharacter":"Acyltransferase domain","value":0,"secondaryStructure":"H"},{"description":"","displayCharacter":"Acyltransferase domain","value":0,"secondaryStructure":"H"},{"description":"","displayCharacter":"Acyltransferase domain","value":0,"secondaryStructure":"H"},{"description":"","displayCharacter":"Acyltransferase domain","value":0,"secondaryStructure":"H"},{"description":"","displayCharacter":"Acyltransferase domain","value":0,"secondaryStructure":"H"},{"description":"","displayCharacter":"Acyltransferase domain","value":0,"secondaryStructure":"H"},{"description":"","displayCharacter":"Acyltransferase domain","value":0,"secondaryStructure":"H"},{"description":"","displayCharacter":"Acyltransferase domain","value":0,"secondaryStructure":"H"},{"description":"","displayCharacter":"Acyltransferase domain","value":0,"secondaryStructure":"H"},{"description":"","displayCharacter":"Acyltransferase domain","value":0,"secondaryStructure":"H"},{"description":"","displayCharacter":"Acyltransferase domain","value":0,"secondaryStructure":"H"},{"description":"","displayCharacter":"Acyltransferase domain","value":0,"secondaryStructure":"H"},{"description":"","displayCharacter":"Acyltransferase domain","value":0,"secondaryStructure":"H"},{"description":"","displayCharacter":"Acyltransferase domain","value":0,"secondaryStructure":"H"},{"description":"","displayCharacter":"Acyltransferase domain","value":0,"secondaryStructure":"H"},{"description":"","displayCharacter":"Acyltransferase domain","value":0,"secondaryStructure":"H"},{"description":"","displayCharacter":"Acyltransferase domain","value":0,"secondaryStructure":"H"},{"description":"","displayCharacter":"Acyltransferase domain","value":0,"secondaryStructure":"H"},{"description":"","displayCharacter":"Acyltransferase domain","value":0,"secondaryStructure":"H"},{"description":"","displayCharacter":"Acyltransferase domain","value":0,"secondaryStructure":"H"},{"description":"","displayCharacter":"Acyltransferase domain","value":0,"secondaryStructure":"H"},{"description":"","displayCharacter":"Acyltransferase domain","value":0,"secondaryStructure":"H"},{"description":"","displayCharacter":"Acyltransferase domain","value":0,"secondaryStructure":"H"},{"description":"","displayCharacter":"Acyltransferase domain","value":0,"secondaryStructure":"H"},{"description":"","displayCharacter":"Acyltransferase domain","value":0,"secondaryStructure":"H"},{"description":"","displayCharacter":"Acyltransferase domain","value":0,"secondaryStructure":"H"},{"description":"","displayCharacter":"Acyltransferase domain","value":0,"secondaryStructure":"H"},{"description":"","displayCharacter":"Acyltransferase domain","value":0,"secondaryStructure":"H"},{"description":"","displayCharacter":"Acyltransferase domain","value":0,"secondaryStructure":"H"},{"description":"","displayCharacter":"Acyltransferase domain","value":0,"secondaryStructure":"H"},{"description":"","displayCharacter":"Acyltransferase domain","value":0,"secondaryStructure":"H"},{"description":"","displayCharacter":"Acyltransferase domain","value":0,"secondaryStructure":"H"},{"description":"","displayCharacter":"Acyltransferase domain","value":0,"secondaryStructure":"H"},{"description":"","displayCharacter":"Acyltransferase domain","value":0,"secondaryStructure":"H"},{"description":"","displayCharacter":"Acyltransferase domain","value":0,"secondaryStructure":"H"},{"description":"","displayCharacter":"Acyltransferase domain","value":0,"secondaryStructure":"H"},{"description":"","displayCharacter":"Acyltransferase domain","value":0,"secondaryStructure":"H"},{"description":"","displayCharacter":"Acyltransferase domain","value":0,"secondaryStructure":"H"},{"description":"","displayCharacter":"Acyltransferase domain","value":0,"secondaryStructure":"H"},{"description":"","displayCharacter":"Acyltransferase domain","value":0,"secondaryStructure":"H"},{"description":"","displayCharacter":"Acyltransferase domain","value":0,"secondaryStructure":"H"},{"description":"","displayCharacter":"Acyltransferase domain","value":0,"secondaryStructure":"H"},{"description":"","displayCharacter":"Acyltransferase domain","value":0,"secondaryStructure":"H"},{"description":"","displayCharacter":"Acyltransferase domain","value":0,"secondaryStructure":"H"},{"description":"","displayCharacter":"Acyltransferase domain","value":0,"secondaryStructure":"H"},{"description":"","displayCharacter":"Acyltransferase domain","value":0,"secondaryStructure":"H"},{"description":"","displayCharacter":"Acyltransferase domain","value":0,"secondaryStructure":"H"},{"description":"","displayCharacter":"Acyltransferase domain","value":0,"secondaryStructure":"H"},{"description":"","displayCharacter":"Acyltransferase domain","value":0,"secondaryStructure":"H"},{"description":"","displayCharacter":"Acyltransferase domain","value":0,"secondaryStructure":"H"},{"description":"","displayCharacter":"Acyltransferase domain","value":0,"secondaryStructure":"H"},{"description":"","displayCharacter":"Acyltransferase domain","value":0,"secondaryStructure":"H"},{"description":"","displayCharacter":"Acyltransferase domain","value":0,"secondaryStructure":"H"},{"description":"","displayCharacter":"Acyltransferase domain","value":0,"secondaryStructure":"H"},{"description":"","displayCharacter":"Acyltransferase domain","value":0,"secondaryStructure":"H"},{"description":"","displayCharacter":"Acyltransferase domain","value":0,"secondaryStructure":"H"},{"description":"","displayCharacter":"Acyltransferase domain","value":0,"secondaryStructure":"H"},{"description":"","displayCharacter":"Acyltransferase domain","value":0,"secondaryStructure":"H"},{"description":"","displayCharacter":"Acyltransferase domain","value":0,"secondaryStructure":"H"},{"description":"","displayCharacter":"Acyltransferase domain","value":0,"secondaryStructure":"H"},{"description":"","displayCharacter":"Acyltransferase domain","value":0,"secondaryStructure":"H"},{"description":"","displayCharacter":"Acyltransferase domain","value":0,"secondaryStructure":"H"},{"description":"","displayCharacter":"Acyltransferase domain","value":0,"secondaryStructure":"H"},{"description":"","displayCharacter":"Acyltransferase domain","value":0,"secondaryStructure":"H"},{"description":"","displayCharacter":"Acyltransferase domain","value":0,"secondaryStructure":"H"},{"description":"","displayCharacter":"Acyltransferase domain","value":0,"secondaryStructure":"H"},{"description":"","displayCharacter":"Acyltransferase domain","value":0,"secondaryStructure":"H"},{"description":"","displayCharacter":"Acyltransferase domain","value":0,"secondaryStructure":"H"},{"description":"","displayCharacter":"Acyltransferase domain","value":0,"secondaryStructure":"H"},{"description":"","displayCharacter":"Acyltransferase domain","value":0,"secondaryStructure":"H"},{"description":"","displayCharacter":"Acyltransferase domain","value":0,"secondaryStructure":"H"},{"description":"","displayCharacter":"Acyltransferase domain","value":0,"secondaryStructure":"H"},{"description":"","displayCharacter":"Acyltransferase domain","value":0,"secondaryStructure":"H"},{"description":"","displayCharacter":"Acyltransferase domain","value":0,"secondaryStructure":"H"},{"description":"","displayCharacter":"Acyltransferase domain","value":0,"secondaryStructure":"H"},{"description":"","displayCharacter":"Acyltransferase domain","value":0,"secondaryStructure":"H"},{"description":"","displayCharacter":"Acyltransferase domain","value":0,"secondaryStructure":"H"},{"description":"","displayCharacter":"Acyltransferase domain","value":0,"secondaryStructure":"H"},{"description":"","displayCharacter":"Acyltransferase domain","value":0,"secondaryStructure":"H"},{"description":"","displayCharacter":"Acyltransferase domain","value":0,"secondaryStructure":"H"},{"description":"","displayCharacter":"Acyltransferase domain","value":0,"secondaryStructure":"H"},{"description":"","displayCharacter":"Acyltransferase domain","value":0,"secondaryStructure":"H"},{"description":"","displayCharacter":"Acyltransferase domain","value":0,"secondaryStructure":"H"},{"description":"","displayCharacter":"Acyltransferase domain","value":0,"secondaryStructure":"H"},{"description":"","displayCharacter":"Acyltransferase domain","value":0,"secondaryStructure":"H"},{"description":"","displayCharacter":"Acyltransferase domain","value":0,"secondaryStructure":"H"},{"description":"","displayCharacter":"Acyltransferase domain","value":0,"secondaryStructure":"H"},{"description":"","displayCharacter":"Acyltransferase domain","value":0,"secondaryStructure":"H"},{"description":"","displayCharacter":"Acyltransferase domain","value":0,"secondaryStructure":"H"},{"description":"","displayCharacter":"Acyltransferase domain","value":0,"secondaryStructure":"H"},{"description":"","displayCharacter":"Acyltransferase domain","value":0,"secondaryStructure":"H"},{"description":"","displayCharacter":"Acyltransferase domain","value":0,"secondaryStructure":"H"},{"description":"","displayCharacter":"Acyltransferase domain","value":0,"secondaryStructure":"H"},{"description":"","displayCharacter":"Acyltransferase domain","value":0,"secondaryStructure":"H"},{"description":"","displayCharacter":"Acyltransferase domain","value":0,"secondaryStructure":"H"},{"description":"","displayCharacter":"Acyltransferase domain","value":0,"secondaryStructure":"H"},{"description":"","displayCharacter":"Acyltransferase domain","value":0,"secondaryStructure":"H"},{"description":"","displayCharacter":"Acyltransferase domain","value":0,"secondaryStructure":"H"},{"description":"","displayCharacter":"Acyltransferase domain","value":0,"secondaryStructure":"H"},{"description":"","displayCharacter":"Acyltransferase domain","value":0,"secondaryStructure":"H"},{"description":"","displayCharacter":"Acyltransferase domain","value":0,"secondaryStructure":"H"},{"description":"","displayCharacter":"Acyltransferase domain","value":0,"secondaryStructure":"H"},{"description":"","displayCharacter":"Acyltransferase domain","value":0,"secondaryStructure":"H"},{"description":"","displayCharacter":"Acyltransferase domain","value":0,"secondaryStructure":"H"},{"description":"","displayCharacter":"Acyltransferase domain","value":0,"secondaryStructure":"H"},{"description":"","displayCharacter":"Acyltransferase domain","value":0,"secondaryStructure":"H"},{"description":"","displayCharacter":"Acyltransferase domain","value":0,"secondaryStructure":"H"},{"description":"","displayCharacter":"Acyltransferase domain","value":0,"secondaryStructure":"H"},{"description":"","displayCharacter":"Acyltransferase domain","value":0,"secondaryStructure":"H"},{"description":"","displayCharacter":"Acyltransferase domain","value":0,"secondaryStructure":"H"},{"description":"","displayCharacter":"Acyltransferase domain","value":0,"secondaryStructure":"H"},{"description":"","displayCharacter":"Acyltransferase domain","value":0,"secondaryStructure":"H"},{"description":"","displayCharacter":"Acyltransferase domain","value":0,"secondaryStructure":"H"},{"description":"","displayCharacter":"Acyltransferase domain","value":0,"secondaryStructure":"H"},{"description":"","displayCharacter":"Acyltransferase domain","value":0,"secondaryStructure":"H"},{"description":"","displayCharacter":"Acyltransferase domain","value":0,"secondaryStructure":"H"},{"description":"","displayCharacter":"Acyltransferase domain","value":0,"secondaryStructure":"H"},{"description":"","displayCharacter":"Acyltransferase domain","value":0,"secondaryStructure":"H"},{"description":"","displayCharacter":"Acyltransferase domain","value":0,"secondaryStructure":"H"},{"description":"","displayCharacter":"Acyltransferase domain","value":0,"secondaryStructure":"H"},{"description":"","displayCharacter":"Acyltransferase domain","value":0,"secondaryStructure":"H"},{"description":"","displayCharacter":"Acyltransferase domain","value":0,"secondaryStructure":"H"},{"description":"","displayCharacter":"Acyltransferase domain","value":0,"secondaryStructure":"H"},{"description":"","displayCharacter":"Acyltransferase domain","value":0,"secondaryStructure":"H"},{"description":"","displayCharacter":"Acyltransferase domain","value":0,"secondaryStructure":"H"},{"description":"","displayCharacter":"Acyltransferase domain","value":0,"secondaryStructure":"H"},{"description":"","displayCharacter":"Acyltransferase domain","value":0,"secondaryStructure":"H"},{"description":"","displayCharacter":"Acyltransferase domain","value":0,"secondaryStructure":"H"},{"description":"","displayCharacter":"Acyltransferase domain","value":0,"secondaryStructure":"H"},{"description":"","displayCharacter":"Acyltransferase domain","value":0,"secondaryStructure":"H"},{"description":"","displayCharacter":"Acyltransferase domain","value":0,"secondaryStructure":"H"},{"description":"","displayCharacter":"Acyltransferase domain","value":0,"secondaryStructure":"H"},{"description":"","displayCharacter":"Acyltransferase domain","value":0,"secondaryStructure":"H"},{"description":"","displayCharacter":"Acyltransferase domain","value":0,"secondaryStructure":"H"},{"description":"","displayCharacter":"Acyltransferase domain","value":0,"secondaryStructure":"H"},{"description":"","displayCharacter":"Acyltransferase domain","value":0,"secondaryStructure":"H"},{"description":"","displayCharacter":"Acyltransferase domain","value":0,"secondaryStructure":"H"},{"description":"","displayCharacter":"Acyltransferase domain","value":0,"secondaryStructure":"H"},{"description":"","displayCharacter":"Acyltransferase domain","value":0,"secondaryStructure":"H"},{"description":"","displayCharacter":"Acyltransferase domain","value":0,"secondaryStructure":"H"},{"description":"","displayCharacter":"Acyltransferase domain","value":0,"secondaryStructure":"H"},{"description":"","displayCharacter":"Acyltransferase domain","value":0,"secondaryStructure":"H"},{"description":"","displayCharacter":"Acyltransferase domain","value":0,"secondaryStructure":"H"},{"description":"","displayCharacter":"Acyltransferase domain","value":0,"secondaryStructure":"H"},{"description":"","displayCharacter":"Acyltransferase domain","value":0,"secondaryStructure":"H"},{"description":"","displayCharacter":"Acyltransferase domain","value":0,"secondaryStructure":"H"},{"description":"","displayCharacter":"Acyltransferase domain","value":0,"secondaryStructure":"H"},{"description":"","displayCharacter":"Acyltransferase domain","value":0,"secondaryStructure":"H"},{"description":"","displayCharacter":"Acyltransferase domain","value":0,"secondaryStructure":"H"},{"description":"","displayCharacter":"Acyltransferase domain","value":0,"secondaryStructure":"H"},{"description":"","displayCharacter":"Acyltransferase domain","value":0,"secondaryStructure":"H"},{"description":"","displayCharacter":"Acyltransferase domain","value":0,"secondaryStructure":"H"},{"value":0,"secondaryStructure":"\u0000"},{"value":0,"secondaryStructure":"\u0000"},{"value":0,"secondaryStructure":"\u0000"},{"value":0,"secondaryStructure":"\u0000"},{"value":0,"secondaryStructure":"\u0000"},{"value":0,"secondaryStructure":"\u0000"},{"value":0,"secondaryStructure":"\u0000"},{"value":0,"secondaryStructure":"\u0000"},{"value":0,"secondaryStructure":"\u0000"},{"value":0,"secondaryStructure":"\u0000"},{"value":0,"secondaryStructure":"\u0000"},{"value":0,"secondaryStructure":"\u0000"},{"value":0,"secondaryStructure":"\u0000"},{"value":0,"secondaryStructure":"\u0000"},{"value":0,"secondaryStructure":"\u0000"},{"value":0,"secondaryStructure":"\u0000"},{"value":0,"secondaryStructure":"\u0000"},{"value":0,"secondaryStructure":"\u0000"},{"value":0,"secondaryStructure":"\u0000"},{"value":0,"secondaryStructure":"\u0000"},{"value":0,"secondaryStructure":"\u0000"},{"value":0,"secondaryStructure":"\u0000"},{"value":0,"secondaryStructure":"\u0000"},{"value":0,"secondaryStructure":"\u0000"},{"value":0,"secondaryStructure":"\u0000"},{"value":0,"secondaryStructure":"\u0000"},{"value":0,"secondaryStructure":"\u0000"},{"value":0,"secondaryStructure":"\u0000"},{"value":0,"secondaryStructure":"\u0000"},{"value":0,"secondaryStructure":"\u0000"},{"value":0,"secondaryStructure":"\u0000"},{"value":0,"secondaryStructure":"\u0000"},{"value":0,"secondaryStructure":"\u0000"},{"value":0,"secondaryStructure":"\u0000"},{"value":0,"secondaryStructure":"\u0000"},{"value":0,"secondaryStructure":"\u0000"},{"value":0,"secondaryStructure":"\u0000"},{"value":0,"secondaryStructure":"\u0000"},{"value":0,"secondaryStructure":"\u0000"},{"value":0,"secondaryStructure":"\u0000"},{"value":0,"secondaryStructure":"\u0000"},{"value":0,"secondaryStructure":"\u0000"},{"value":0,"secondaryStructure":"\u0000"},{"value":0,"secondaryStructure":"\u0000"},{"value":0,"secondaryStructure":"\u0000"},{"value":0,"secondaryStructure":"\u0000"},{"value":0,"secondaryStructure":"\u0000"},{"value":0,"secondaryStructure":"\u0000"},{"value":0,"secondaryStructure":"\u0000"},{"value":0,"secondaryStructure":"\u0000"},{"value":0,"secondaryStructure":"\u0000"},{"value":0,"secondaryStructure":"\u0000"},{"value":0,"secondaryStructure":"\u0000"},{"value":0,"secondaryStructure":"\u0000"},{"value":0,"secondaryStructure":"\u0000"},{"value":0,"secondaryStructure":"\u0000"},{"value":0,"secondaryStructure":"\u0000"},{"value":0,"secondaryStructure":"\u0000"},{"value":0,"secondaryStructure":"\u0000"},{"value":0,"secondaryStructure":"\u0000"},{"value":0,"secondaryStructure":"\u0000"},{"value":0,"secondaryStructure":"\u0000"},{"value":0,"secondaryStructure":"\u0000"},{"value":0,"secondaryStructure":"\u0000"},{"value":0,"secondaryStructure":"\u0000"},{"value":0,"secondaryStructure":"\u0000"},{"value":0,"secondaryStructure":"\u0000"},{"value":0,"secondaryStructure":"\u0000"},{"value":0,"secondaryStructure":"\u0000"},{"value":0,"secondaryStructure":"\u0000"},{"value":0,"secondaryStructure":"\u0000"},{"value":0,"secondaryStructure":"\u0000"},{"value":0,"secondaryStructure":"\u0000"},{"value":0,"secondaryStructure":"\u0000"},{"value":0,"secondaryStructure":"\u0000"},{"value":0,"secondaryStructure":"\u0000"},{"value":0,"secondaryStructure":"\u0000"},{"value":0,"secondaryStructure":"\u0000"},{"value":0,"secondaryStructure":"\u0000"},{"value":0,"secondaryStructure":"\u0000"},{"value":0,"secondaryStructure":"\u0000"},{"value":0,"secondaryStructure":"\u0000"},{"value":0,"secondaryStructure":"\u0000"},{"value":0,"secondaryStructure":"\u0000"},{"value":0,"secondaryStructure":"\u0000"},{"value":0,"secondaryStructure":"\u0000"},{"value":0,"secondaryStructure":"\u0000"},{"value":0,"secondaryStructure":"\u0000"},{"value":0,"secondaryStructure":"\u0000"},{"value":0,"secondaryStructure":"\u0000"},{"description":"","displayCharacter":"Dehydratase domain","value":0,"secondaryStructure":"H"},{"description":"","displayCharacter":"Dehydratase domain","value":0,"secondaryStructure":"H"},{"description":"","displayCharacter":"Dehydratase domain","value":0,"secondaryStructure":"H"},{"description":"","displayCharacter":"Dehydratase domain","value":0,"secondaryStructure":"H"},{"description":"","displayCharacter":"Dehydratase domain","value":0,"secondaryStructure":"H"},{"description":"","displayCharacter":"Dehydratase domain","value":0,"secondaryStructure":"H"},{"description":"","displayCharacter":"Dehydratase domain","value":0,"secondaryStructure":"H"},{"description":"","displayCharacter":"Dehydratase domain","value":0,"secondaryStructure":"H"},{"description":"","displayCharacter":"Dehydratase domain","value":0,"secondaryStructure":"H"},{"description":"","displayCharacter":"Dehydratase domain","value":0,"secondaryStructure":"H"},{"description":"","displayCharacter":"Dehydratase domain","value":0,"secondaryStructure":"H"},{"description":"","displayCharacter":"Dehydratase domain","value":0,"secondaryStructure":"H"},{"description":"","displayCharacter":"Dehydratase domain","value":0,"secondaryStructure":"H"},{"description":"","displayCharacter":"Dehydratase domain","value":0,"secondaryStructure":"H"},{"description":"","displayCharacter":"Dehydratase domain","value":0,"secondaryStructure":"H"},{"description":"","displayCharacter":"Dehydratase domain","value":0,"secondaryStructure":"H"},{"description":"","displayCharacter":"Dehydratase domain","value":0,"secondaryStructure":"H"},{"description":"","displayCharacter":"Dehydratase domain","value":0,"secondaryStructure":"H"},{"description":"","displayCharacter":"Dehydratase domain","value":0,"secondaryStructure":"H"},{"description":"","displayCharacter":"Dehydratase domain","value":0,"secondaryStructure":"H"},{"description":"","displayCharacter":"Dehydratase domain","value":0,"secondaryStructure":"H"},{"description":"","displayCharacter":"Dehydratase domain","value":0,"secondaryStructure":"H"},{"description":"","displayCharacter":"Dehydratase domain","value":0,"secondaryStructure":"H"},{"description":"","displayCharacter":"Dehydratase domain","value":0,"secondaryStructure":"H"},{"description":"","displayCharacter":"Dehydratase domain","value":0,"secondaryStructure":"H"},{"description":"","displayCharacter":"Dehydratase domain","value":0,"secondaryStructure":"H"},{"description":"","displayCharacter":"Dehydratase domain","value":0,"secondaryStructure":"H"},{"description":"","displayCharacter":"Dehydratase domain","value":0,"secondaryStructure":"H"},{"description":"","displayCharacter":"Dehydratase domain","value":0,"secondaryStructure":"H"},{"description":"","displayCharacter":"Dehydratase domain","value":0,"secondaryStructure":"H"},{"description":"","displayCharacter":"Dehydratase domain","value":0,"secondaryStructure":"H"},{"description":"","displayCharacter":"Dehydratase domain","value":0,"secondaryStructure":"H"},{"description":"","displayCharacter":"Dehydratase domain","value":0,"secondaryStructure":"H"},{"description":"","displayCharacter":"Dehydratase domain","value":0,"secondaryStructure":"H"},{"description":"","displayCharacter":"Dehydratase domain","value":0,"secondaryStructure":"H"},{"description":"","displayCharacter":"Dehydratase domain","value":0,"secondaryStructure":"H"},{"description":"","displayCharacter":"Dehydratase domain","value":0,"secondaryStructure":"H"},{"description":"","displayCharacter":"Dehydratase domain","value":0,"secondaryStructure":"H"},{"description":"","displayCharacter":"Dehydratase domain","value":0,"secondaryStructure":"H"},{"description":"","displayCharacter":"Dehydratase domain","value":0,"secondaryStructure":"H"},{"description":"","displayCharacter":"Dehydratase domain","value":0,"secondaryStructure":"H"},{"description":"","displayCharacter":"Dehydratase domain","value":0,"secondaryStructure":"H"},{"description":"","displayCharacter":"Dehydratase domain","value":0,"secondaryStructure":"H"},{"description":"","displayCharacter":"Dehydratase domain","value":0,"secondaryStructure":"H"},{"description":"","displayCharacter":"Dehydratase domain","value":0,"secondaryStructure":"H"},{"description":"","displayCharacter":"Dehydratase domain","value":0,"secondaryStructure":"H"},{"description":"","displayCharacter":"Dehydratase domain","value":0,"secondaryStructure":"H"},{"description":"","displayCharacter":"Dehydratase domain","value":0,"secondaryStructure":"H"},{"description":"","displayCharacter":"Dehydratase domain","value":0,"secondaryStructure":"H"},{"description":"","displayCharacter":"Dehydratase domain","value":0,"secondaryStructure":"H"},{"description":"","displayCharacter":"Dehydratase domain","value":0,"secondaryStructure":"H"},{"description":"","displayCharacter":"Dehydratase domain","value":0,"secondaryStructure":"H"},{"description":"","displayCharacter":"Dehydratase domain","value":0,"secondaryStructure":"H"},{"description":"","displayCharacter":"Dehydratase domain","value":0,"secondaryStructure":"H"},{"description":"","displayCharacter":"Dehydratase domain","value":0,"secondaryStructure":"H"},{"description":"","displayCharacter":"Dehydratase domain","value":0,"secondaryStructure":"H"},{"description":"","displayCharacter":"Dehydratase domain","value":0,"secondaryStructure":"H"},{"description":"","displayCharacter":"Dehydratase domain","value":0,"secondaryStructure":"H"},{"description":"","displayCharacter":"Dehydratase domain","value":0,"secondaryStructure":"H"},{"description":"","displayCharacter":"Dehydratase domain","value":0,"secondaryStructure":"H"},{"description":"","displayCharacter":"Dehydratase domain","value":0,"secondaryStructure":"H"},{"description":"","displayCharacter":"Dehydratase domain","value":0,"secondaryStructure":"H"},{"description":"","displayCharacter":"Dehydratase domain","value":0,"secondaryStructure":"H"},{"description":"","displayCharacter":"Dehydratase domain","value":0,"secondaryStructure":"H"},{"description":"","displayCharacter":"Dehydratase domain","value":0,"secondaryStructure":"H"},{"description":"","displayCharacter":"Dehydratase domain","value":0,"secondaryStructure":"H"},{"description":"","displayCharacter":"Dehydratase domain","value":0,"secondaryStructure":"H"},{"description":"","displayCharacter":"Dehydratase domain","value":0,"secondaryStructure":"H"},{"description":"","displayCharacter":"Dehydratase domain","value":0,"secondaryStructure":"H"},{"description":"","displayCharacter":"Dehydratase domain","value":0,"secondaryStructure":"H"},{"description":"","displayCharacter":"Dehydratase domain","value":0,"secondaryStructure":"H"},{"description":"","displayCharacter":"Dehydratase domain","value":0,"secondaryStructure":"H"},{"description":"","displayCharacter":"Dehydratase domain","value":0,"secondaryStructure":"H"},{"description":"","displayCharacter":"Dehydratase domain","value":0,"secondaryStructure":"H"},{"description":"","displayCharacter":"Dehydratase domain","value":0,"secondaryStructure":"H"},{"description":"","displayCharacter":"Dehydratase domain","value":0,"secondaryStructure":"H"},{"description":"","displayCharacter":"Dehydratase domain","value":0,"secondaryStructure":"H"},{"description":"","displayCharacter":"Dehydratase domain","value":0,"secondaryStructure":"H"},{"description":"","displayCharacter":"Dehydratase domain","value":0,"secondaryStructure":"H"},{"description":"","displayCharacter":"Dehydratase domain","value":0,"secondaryStructure":"H"},{"description":"","displayCharacter":"Dehydratase domain","value":0,"secondaryStructure":"H"},{"description":"","displayCharacter":"Dehydratase domain","value":0,"secondaryStructure":"H"},{"description":"","displayCharacter":"Dehydratase domain","value":0,"secondaryStructure":"H"},{"description":"","displayCharacter":"Dehydratase domain","value":0,"secondaryStructure":"H"},{"description":"","displayCharacter":"Dehydratase domain","value":0,"secondaryStructure":"H"},{"description":"","displayCharacter":"Dehydratase domain","value":0,"secondaryStructure":"H"},{"description":"","displayCharacter":"Dehydratase domain","value":0,"secondaryStructure":"H"},{"description":"","displayCharacter":"Dehydratase domain","value":0,"secondaryStructure":"H"},{"description":"","displayCharacter":"Dehydratase domain","value":0,"secondaryStructure":"H"},{"description":"","displayCharacter":"Dehydratase domain","value":0,"secondaryStructure":"H"},{"description":"","displayCharacter":"Dehydratase domain","value":0,"secondaryStructure":"H"},{"description":"","displayCharacter":"Dehydratase domain","value":0,"secondaryStructure":"H"},{"description":"","displayCharacter":"Dehydratase domain","value":0,"secondaryStructure":"H"},{"description":"","displayCharacter":"Dehydratase domain","value":0,"secondaryStructure":"H"},{"description":"","displayCharacter":"Dehydratase domain","value":0,"secondaryStructure":"H"},{"description":"","displayCharacter":"Dehydratase domain","value":0,"secondaryStructure":"H"},{"description":"","displayCharacter":"Dehydratase domain","value":0,"secondaryStructure":"H"},{"description":"","displayCharacter":"Dehydratase domain","value":0,"secondaryStructure":"H"},{"description":"","displayCharacter":"Dehydratase domain","value":0,"secondaryStructure":"H"},{"description":"","displayCharacter":"Dehydratase domain","value":0,"secondaryStructure":"H"},{"description":"","displayCharacter":"Dehydratase domain","value":0,"secondaryStructure":"H"},{"description":"","displayCharacter":"Dehydratase domain","value":0,"secondaryStructure":"H"},{"description":"","displayCharacter":"Dehydratase domain","value":0,"secondaryStructure":"H"},{"description":"","displayCharacter":"Dehydratase domain","value":0,"secondaryStructure":"H"},{"description":"","displayCharacter":"Dehydratase domain","value":0,"secondaryStructure":"H"},{"description":"","displayCharacter":"Dehydratase domain","value":0,"secondaryStructure":"H"},{"description":"","displayCharacter":"Dehydratase domain","value":0,"secondaryStructure":"H"},{"description":"","displayCharacter":"Dehydratase domain","value":0,"secondaryStructure":"H"},{"description":"","displayCharacter":"Dehydratase domain","value":0,"secondaryStructure":"H"},{"description":"","displayCharacter":"Dehydratase domain","value":0,"secondaryStructure":"H"},{"description":"","displayCharacter":"Dehydratase domain","value":0,"secondaryStructure":"H"},{"description":"","displayCharacter":"Dehydratase domain","value":0,"secondaryStructure":"H"},{"description":"","displayCharacter":"Dehydratase domain","value":0,"secondaryStructure":"H"},{"description":"","displayCharacter":"Dehydratase domain","value":0,"secondaryStructure":"H"},{"description":"","displayCharacter":"Dehydratase domain","value":0,"secondaryStructure":"H"},{"description":"","displayCharacter":"Dehydratase domain","value":0,"secondaryStructure":"H"},{"description":"","displayCharacter":"Dehydratase domain","value":0,"secondaryStructure":"H"},{"description":"","displayCharacter":"Dehydratase domain","value":0,"secondaryStructure":"H"},{"description":"","displayCharacter":"Dehydratase domain","value":0,"secondaryStructure":"H"},{"description":"","displayCharacter":"Dehydratase domain","value":0,"secondaryStructure":"H"},{"description":"","displayCharacter":"Dehydratase domain","value":0,"secondaryStructure":"H"},{"description":"","displayCharacter":"Dehydratase domain","value":0,"secondaryStructure":"H"},{"description":"","displayCharacter":"Dehydratase domain","value":0,"secondaryStructure":"H"},{"description":"","displayCharacter":"Dehydratase domain","value":0,"secondaryStructure":"H"},{"description":"","displayCharacter":"Dehydratase domain","value":0,"secondaryStructure":"H"},{"description":"","displayCharacter":"Dehydratase domain","value":0,"secondaryStructure":"H"},{"description":"","displayCharacter":"Dehydratase domain","value":0,"secondaryStructure":"H"},{"description":"","displayCharacter":"Dehydratase domain","value":0,"secondaryStructure":"H"},{"description":"","displayCharacter":"Dehydratase domain","value":0,"secondaryStructure":"H"},{"description":"","displayCharacter":"Dehydratase domain","value":0,"secondaryStructure":"H"},{"description":"","displayCharacter":"Dehydratase domain","value":0,"secondaryStructure":"H"},{"description":"","displayCharacter":"Dehydratase domain","value":0,"secondaryStructure":"H"},{"description":"","displayCharacter":"Dehydratase domain","value":0,"secondaryStructure":"H"},{"description":"","displayCharacter":"Dehydratase domain","value":0,"secondaryStructure":"H"},{"description":"","displayCharacter":"Dehydratase domain","value":0,"secondaryStructure":"H"},{"description":"","displayCharacter":"Dehydratase domain","value":0,"secondaryStructure":"H"},{"description":"","displayCharacter":"Dehydratase domain","value":0,"secondaryStructure":"H"},{"description":"","displayCharacter":"Dehydratase domain","value":0,"secondaryStructure":"H"},{"description":"","displayCharacter":"Dehydratase domain","value":0,"secondaryStructure":"H"},{"description":"","displayCharacter":"Dehydratase domain","value":0,"secondaryStructure":"H"},{"description":"","displayCharacter":"Dehydratase domain","value":0,"secondaryStructure":"H"},{"description":"","displayCharacter":"Dehydratase domain","value":0,"secondaryStructure":"H"},{"description":"","displayCharacter":"Dehydratase domain","value":0,"secondaryStructure":"H"},{"description":"","displayCharacter":"Dehydratase domain","value":0,"secondaryStructure":"H"},{"description":"","displayCharacter":"Dehydratase domain","value":0,"secondaryStructure":"H"},{"description":"","displayCharacter":"Dehydratase domain","value":0,"secondaryStructure":"H"},{"description":"","displayCharacter":"Dehydratase domain","value":0,"secondaryStructure":"H"},{"description":"","displayCharacter":"Dehydratase domain","value":0,"secondaryStructure":"H"},{"description":"","displayCharacter":"Dehydratase domain","value":0,"secondaryStructure":"H"},{"description":"","displayCharacter":"Dehydratase domain","value":0,"secondaryStructure":"H"},{"description":"","displayCharacter":"Dehydratase domain","value":0,"secondaryStructure":"H"},{"description":"","displayCharacter":"Dehydratase domain","value":0,"secondaryStructure":"H"},{"description":"","displayCharacter":"Dehydratase domain","value":0,"secondaryStructure":"H"},{"description":"","displayCharacter":"Dehydratase domain","value":0,"secondaryStructure":"H"},{"description":"","displayCharacter":"Dehydratase domain","value":0,"secondaryStructure":"H"},{"description":"","displayCharacter":"Dehydratase domain","value":0,"secondaryStructure":"H"},{"description":"","displayCharacter":"Dehydratase domain","value":0,"secondaryStructure":"H"},{"description":"","displayCharacter":"Dehydratase domain","value":0,"secondaryStructure":"H"},{"description":"","displayCharacter":"Dehydratase domain","value":0,"secondaryStructure":"H"},{"description":"","displayCharacter":"Dehydratase domain","value":0,"secondaryStructure":"H"},{"description":"","displayCharacter":"Dehydratase domain","value":0,"secondaryStructure":"H"},{"description":"","displayCharacter":"Dehydratase domain","value":0,"secondaryStructure":"H"},{"description":"","displayCharacter":"Dehydratase domain","value":0,"secondaryStructure":"H"},{"description":"","displayCharacter":"Dehydratase domain","value":0,"secondaryStructure":"H"},{"description":"","displayCharacter":"Dehydratase domain","value":0,"secondaryStructure":"H"},{"description":"","displayCharacter":"Dehydratase domain","value":0,"secondaryStructure":"H"},{"description":"","displayCharacter":"Dehydratase domain","value":0,"secondaryStructure":"H"},{"description":"","displayCharacter":"Dehydratase domain","value":0,"secondaryStructure":"H"},{"description":"","displayCharacter":"Dehydratase domain","value":0,"secondaryStructure":"H"},{"description":"","displayCharacter":"Dehydratase domain","value":0,"secondaryStructure":"H"},{"description":"","displayCharacter":"Dehydratase domain","value":0,"secondaryStructure":"H"},{"description":"","displayCharacter":"Dehydratase domain","value":0,"secondaryStructure":"H"},{"description":"","displayCharacter":"Dehydratase domain","value":0,"secondaryStructure":"H"},{"description":"","displayCharacter":"Dehydratase domain","value":0,"secondaryStructure":"H"},{"description":"","displayCharacter":"Dehydratase domain","value":0,"secondaryStructure":"H"},{"description":"","displayCharacter":"Dehydratase domain","value":0,"secondaryStructure":"H"},{"description":"","displayCharacter":"Dehydratase domain","value":0,"secondaryStructure":"H"},{"description":"","displayCharacter":"Dehydratase domain","value":0,"secondaryStructure":"H"},{"description":"","displayCharacter":"Dehydratase domain","value":0,"secondaryStructure":"H"},{"description":"","displayCharacter":"Dehydratase domain","value":0,"secondaryStructure":"H"},{"description":"","displayCharacter":"Dehydratase domain","value":0,"secondaryStructure":"H"},{"description":"","displayCharacter":"Dehydratase domain","value":0,"secondaryStructure":"H"},{"description":"","displayCharacter":"Dehydratase domain","value":0,"secondaryStructure":"H"},{"description":"","displayCharacter":"Dehydratase domain","value":0,"secondaryStructure":"H"},{"description":"","displayCharacter":"Dehydratase domain","value":0,"secondaryStructure":"H"},{"description":"","displayCharacter":"Dehydratase domain","value":0,"secondaryStructure":"H"},{"description":"","displayCharacter":"Dehydratase domain","value":0,"secondaryStructure":"H"},{"description":"","displayCharacter":"Dehydratase domain","value":0,"secondaryStructure":"H"},{"description":"","displayCharacter":"Dehydratase domain","value":0,"secondaryStructure":"H"},{"description":"","displayCharacter":"Dehydratase domain","value":0,"secondaryStructure":"H"},{"description":"","displayCharacter":"Dehydratase domain","value":0,"secondaryStructure":"H"},{"description":"","displayCharacter":"Dehydratase domain","value":0,"secondaryStructure":"H"},{"description":"","displayCharacter":"Dehydratase domain","value":0,"secondaryStructure":"H"},{"description":"","displayCharacter":"Dehydratase domain","value":0,"secondaryStructure":"H"},{"description":"","displayCharacter":"Dehydratase domain","value":0,"secondaryStructure":"H"},{"description":"","displayCharacter":"Dehydratase domain","value":0,"secondaryStructure":"H"},{"description":"","displayCharacter":"Dehydratase domain","value":0,"secondaryStructure":"H"},{"description":"","displayCharacter":"Dehydratase domain","value":0,"secondaryStructure":"H"},{"description":"","displayCharacter":"Dehydratase domain","value":0,"secondaryStructure":"H"},{"description":"","displayCharacter":"Dehydratase domain","value":0,"secondaryStructure":"H"},{"description":"","displayCharacter":"Dehydratase domain","value":0,"secondaryStructure":"H"},{"description":"","displayCharacter":"Dehydratase domain","value":0,"secondaryStructure":"H"},{"description":"","displayCharacter":"Dehydratase domain","value":0,"secondaryStructure":"H"},{"description":"","displayCharacter":"Dehydratase domain","value":0,"secondaryStructure":"H"},{"description":"","displayCharacter":"Dehydratase domain","value":0,"secondaryStructure":"H"},{"description":"","displayCharacter":"Dehydratase domain","value":0,"secondaryStructure":"H"},{"description":"","displayCharacter":"Dehydratase domain","value":0,"secondaryStructure":"H"},{"description":"","displayCharacter":"Dehydratase domain","value":0,"secondaryStructure":"H"},{"description":"","displayCharacter":"Dehydratase domain","value":0,"secondaryStructure":"H"},{"description":"","displayCharacter":"Dehydratase domain","value":0,"secondaryStructure":"H"},{"description":"","displayCharacter":"Dehydratase domain","value":0,"secondaryStructure":"H"},{"description":"","displayCharacter":"Dehydratase domain","value":0,"secondaryStructure":"H"},{"description":"","displayCharacter":"Dehydratase domain","value":0,"secondaryStructure":"H"},{"description":"","displayCharacter":"Dehydratase domain","value":0,"secondaryStructure":"H"},{"description":"","displayCharacter":"Dehydratase domain","value":0,"secondaryStructure":"H"},{"description":"","displayCharacter":"Dehydratase domain","value":0,"secondaryStructure":"H"},{"description":"","displayCharacter":"Dehydratase domain","value":0,"secondaryStructure":"H"},{"description":"","displayCharacter":"Dehydratase domain","value":0,"secondaryStructure":"H"},{"description":"","displayCharacter":"Dehydratase domain","value":0,"secondaryStructure":"H"},{"description":"","displayCharacter":"Dehydratase domain","value":0,"secondaryStructure":"H"},{"description":"","displayCharacter":"Dehydratase domain","value":0,"secondaryStructure":"H"},{"description":"","displayCharacter":"Dehydratase domain","value":0,"secondaryStructure":"H"},{"description":"","displayCharacter":"Dehydratase domain","value":0,"secondaryStructure":"H"},{"description":"","displayCharacter":"Dehydratase domain","value":0,"secondaryStructure":"H"},{"description":"","displayCharacter":"Dehydratase domain","value":0,"secondaryStructure":"H"},{"description":"","displayCharacter":"Dehydratase domain","value":0,"secondaryStructure":"H"},{"description":"","displayCharacter":"Dehydratase domain","value":0,"secondaryStructure":"H"},{"description":"","displayCharacter":"Dehydratase domain","value":0,"secondaryStructure":"H"},{"description":"","displayCharacter":"Dehydratase domain","value":0,"secondaryStructure":"H"},{"description":"","displayCharacter":"Dehydratase domain","value":0,"secondaryStructure":"H"},{"description":"","displayCharacter":"Dehydratase domain","value":0,"secondaryStructure":"H"},{"description":"","displayCharacter":"Dehydratase domain","value":0,"secondaryStructure":"H"},{"description":"","displayCharacter":"Dehydratase domain","value":0,"secondaryStructure":"H"},{"description":"","displayCharacter":"Dehydratase domain","value":0,"secondaryStructure":"H"},{"description":"","displayCharacter":"Dehydratase domain","value":0,"secondaryStructure":"H"},{"description":"","displayCharacter":"Dehydratase domain","value":0,"secondaryStructure":"H"},{"description":"","displayCharacter":"Dehydratase domain","value":0,"secondaryStructure":"H"},{"description":"","displayCharacter":"Dehydratase domain","value":0,"secondaryStructure":"H"},{"description":"","displayCharacter":"Dehydratase domain","value":0,"secondaryStructure":"H"},{"description":"","displayCharacter":"Dehydratase domain","value":0,"secondaryStructure":"H"},{"description":"","displayCharacter":"Dehydratase domain","value":0,"secondaryStructure":"H"},{"description":"","displayCharacter":"Dehydratase domain","value":0,"secondaryStructure":"H"},{"description":"","displayCharacter":"Dehydratase domain","value":0,"secondaryStructure":"H"},{"description":"","displayCharacter":"Dehydratase domain","value":0,"secondaryStructure":"H"},{"description":"","displayCharacter":"Dehydratase domain","value":0,"secondaryStructure":"H"},{"description":"","displayCharacter":"Dehydratase domain","value":0,"secondaryStructure":"H"},{"value":0,"secondaryStructure":"\u0000"},{"value":0,"secondaryStructure":"\u0000"},{"value":0,"secondaryStructure":"\u0000"},{"value":0,"secondaryStructure":"\u0000"},{"value":0,"secondaryStructure":"\u0000"},{"value":0,"secondaryStructure":"\u0000"},{"value":0,"secondaryStructure":"\u0000"},{"value":0,"secondaryStructure":"\u0000"},{"value":0,"secondaryStructure":"\u0000"},{"value":0,"secondaryStructure":"\u0000"},{"value":0,"secondaryStructure":"\u0000"},{"value":0,"secondaryStructure":"\u0000"},{"value":0,"secondaryStructure":"\u0000"},{"value":0,"secondaryStructure":"\u0000"},{"value":0,"secondaryStructure":"\u0000"},{"value":0,"secondaryStructure":"\u0000"},{"value":0,"secondaryStructure":"\u0000"},{"value":0,"secondaryStructure":"\u0000"},{"value":0,"secondaryStructure":"\u0000"},{"value":0,"secondaryStructure":"\u0000"},{"value":0,"secondaryStructure":"\u0000"},{"value":0,"secondaryStructure":"\u0000"},{"value":0,"secondaryStructure":"\u0000"},{"value":0,"secondaryStructure":"\u0000"},{"value":0,"secondaryStructure":"\u0000"},{"value":0,"secondaryStructure":"\u0000"},{"value":0,"secondaryStructure":"\u0000"},{"value":0,"secondaryStructure":"\u0000"},{"value":0,"secondaryStructure":"\u0000"},{"value":0,"secondaryStructure":"\u0000"},{"value":0,"secondaryStructure":"\u0000"},{"value":0,"secondaryStructure":"\u0000"},{"value":0,"secondaryStructure":"\u0000"},{"value":0,"secondaryStructure":"\u0000"},{"value":0,"secondaryStructure":"\u0000"},{"value":0,"secondaryStructure":"\u0000"},{"value":0,"secondaryStructure":"\u0000"},{"value":0,"secondaryStructure":"\u0000"},{"value":0,"secondaryStructure":"\u0000"},{"value":0,"secondaryStructure":"\u0000"},{"value":0,"secondaryStructure":"\u0000"},{"value":0,"secondaryStructure":"\u0000"},{"value":0,"secondaryStructure":"\u0000"},{"value":0,"secondaryStructure":"\u0000"},{"value":0,"secondaryStructure":"\u0000"},{"value":0,"secondaryStructure":"\u0000"},{"value":0,"secondaryStructure":"\u0000"},{"value":0,"secondaryStructure":"\u0000"},{"value":0,"secondaryStructure":"\u0000"},{"value":0,"secondaryStructure":"\u0000"},{"value":0,"secondaryStructure":"\u0000"},{"value":0,"secondaryStructure":"\u0000"},{"value":0,"secondaryStructure":"\u0000"},{"value":0,"secondaryStructure":"\u0000"},{"value":0,"secondaryStructure":"\u0000"},{"value":0,"secondaryStructure":"\u0000"},{"value":0,"secondaryStructure":"\u0000"},{"value":0,"secondaryStructure":"\u0000"},{"value":0,"secondaryStructure":"\u0000"},{"value":0,"secondaryStructure":"\u0000"},{"value":0,"secondaryStructure":"\u0000"},{"value":0,"secondaryStructure":"\u0000"},{"value":0,"secondaryStructure":"\u0000"},{"value":0,"secondaryStructure":"\u0000"},{"value":0,"secondaryStructure":"\u0000"},{"value":0,"secondaryStructure":"\u0000"},{"value":0,"secondaryStructure":"\u0000"},{"value":0,"secondaryStructure":"\u0000"},{"value":0,"secondaryStructure":"\u0000"},{"value":0,"secondaryStructure":"\u0000"},{"value":0,"secondaryStructure":"\u0000"},{"value":0,"secondaryStructure":"\u0000"},{"value":0,"secondaryStructure":"\u0000"},{"value":0,"secondaryStructure":"\u0000"},{"value":0,"secondaryStructure":"\u0000"},{"value":0,"secondaryStructure":"\u0000"},{"value":0,"secondaryStructure":"\u0000"},{"value":0,"secondaryStructure":"\u0000"},{"value":0,"secondaryStructure":"\u0000"},{"value":0,"secondaryStructure":"\u0000"},{"value":0,"secondaryStructure":"\u0000"},{"value":0,"secondaryStructure":"\u0000"},{"value":0,"secondaryStructure":"\u0000"},{"value":0,"secondaryStructure":"\u0000"},{"value":0,"secondaryStructure":"\u0000"},{"value":0,"secondaryStructure":"\u0000"},{"value":0,"secondaryStructure":"\u0000"},{"value":0,"secondaryStructure":"\u0000"},{"value":0,"secondaryStructure":"\u0000"},{"value":0,"secondaryStructure":"\u0000"},{"value":0,"secondaryStructure":"\u0000"},{"value":0,"secondaryStructure":"\u0000"},{"value":0,"secondaryStructure":"\u0000"},{"value":0,"secondaryStructure":"\u0000"},{"value":0,"secondaryStructure":"\u0000"},{"value":0,"secondaryStructure":"\u0000"},{"value":0,"secondaryStructure":"\u0000"},{"value":0,"secondaryStructure":"\u0000"},{"value":0,"secondaryStructure":"\u0000"},{"value":0,"secondaryStructure":"\u0000"},{"value":0,"secondaryStructure":"\u0000"},{"value":0,"secondaryStructure":"\u0000"},{"value":0,"secondaryStructure":"\u0000"},{"value":0,"secondaryStructure":"\u0000"},{"value":0,"secondaryStructure":"\u0000"},{"value":0,"secondaryStructure":"\u0000"},{"value":0,"secondaryStructure":"\u0000"},{"value":0,"secondaryStructure":"\u0000"},{"value":0,"secondaryStructure":"\u0000"},{"value":0,"secondaryStructure":"\u0000"},{"value":0,"secondaryStructure":"\u0000"},{"value":0,"secondaryStructure":"\u0000"},{"value":0,"secondaryStructure":"\u0000"},{"value":0,"secondaryStructure":"\u0000"},{"value":0,"secondaryStructure":"\u0000"},{"value":0,"secondaryStructure":"\u0000"},{"value":0,"secondaryStructure":"\u0000"},{"value":0,"secondaryStructure":"\u0000"},{"value":0,"secondaryStructure":"\u0000"},{"value":0,"secondaryStructure":"\u0000"},{"value":0,"secondaryStructure":"\u0000"},{"value":0,"secondaryStructure":"\u0000"},{"value":0,"secondaryStructure":"\u0000"},{"value":0,"secondaryStructure":"\u0000"},{"value":0,"secondaryStructure":"\u0000"},{"value":0,"secondaryStructure":"\u0000"},{"value":0,"secondaryStructure":"\u0000"},{"value":0,"secondaryStructure":"\u0000"},{"value":0,"secondaryStructure":"\u0000"},{"value":0,"secondaryStructure":"\u0000"},{"value":0,"secondaryStructure":"\u0000"},{"value":0,"secondaryStructure":"\u0000"},{"value":0,"secondaryStructure":"\u0000"},{"value":0,"secondaryStructure":"\u0000"},{"value":0,"secondaryStructure":"\u0000"},{"value":0,"secondaryStructure":"\u0000"},{"value":0,"secondaryStructure":"\u0000"},{"value":0,"secondaryStructure":"\u0000"},{"value":0,"secondaryStructure":"\u0000"},{"value":0,"secondaryStructure":"\u0000"},{"value":0,"secondaryStructure":"\u0000"},{"value":0,"secondaryStructure":"\u0000"},{"value":0,"secondaryStructure":"\u0000"},{"value":0,"secondaryStructure":"\u0000"},{"value":0,"secondaryStructure":"\u0000"},{"value":0,"secondaryStructure":"\u0000"},{"value":0,"secondaryStructure":"\u0000"},{"value":0,"secondaryStructure":"\u0000"},{"value":0,"secondaryStructure":"\u0000"},{"value":0,"secondaryStructure":"\u0000"},{"value":0,"secondaryStructure":"\u0000"},{"value":0,"secondaryStructure":"\u0000"},{"value":0,"secondaryStructure":"\u0000"},{"value":0,"secondaryStructure":"\u0000"},{"value":0,"secondaryStructure":"\u0000"},{"value":0,"secondaryStructure":"\u0000"},{"value":0,"secondaryStructure":"\u0000"},{"value":0,"secondaryStructure":"\u0000"},{"value":0,"secondaryStructure":"\u0000"},{"value":0,"secondaryStructure":"\u0000"},{"value":0,"secondaryStructure":"\u0000"},{"value":0,"secondaryStructure":"\u0000"},{"value":0,"secondaryStructure":"\u0000"},{"value":0,"secondaryStructure":"\u0000"},{"value":0,"secondaryStructure":"\u0000"},{"value":0,"secondaryStructure":"\u0000"},{"value":0,"secondaryStructure":"\u0000"},{"value":0,"secondaryStructure":"\u0000"},{"value":0,"secondaryStructure":"\u0000"},{"value":0,"secondaryStructure":"\u0000"},{"value":0,"secondaryStructure":"\u0000"},{"value":0,"secondaryStructure":"\u0000"},{"value":0,"secondaryStructure":"\u0000"},{"value":0,"secondaryStructure":"\u0000"},{"value":0,"secondaryStructure":"\u0000"},{"value":0,"secondaryStructure":"\u0000"},{"value":0,"secondaryStructure":"\u0000"},{"value":0,"secondaryStructure":"\u0000"},{"value":0,"secondaryStructure":"\u0000"},{"value":0,"secondaryStructure":"\u0000"},{"value":0,"secondaryStructure":"\u0000"},{"value":0,"secondaryStructure":"\u0000"},{"value":0,"secondaryStructure":"\u0000"},{"value":0,"secondaryStructure":"\u0000"},{"value":0,"secondaryStructure":"\u0000"},{"value":0,"secondaryStructure":"\u0000"},{"value":0,"secondaryStructure":"\u0000"},{"value":0,"secondaryStructure":"\u0000"},{"value":0,"secondaryStructure":"\u0000"},{"value":0,"secondaryStructure":"\u0000"},{"value":0,"secondaryStructure":"\u0000"},{"value":0,"secondaryStructure":"\u0000"},{"value":0,"secondaryStructure":"\u0000"},{"value":0,"secondaryStructure":"\u0000"},{"value":0,"secondaryStructure":"\u0000"},{"value":0,"secondaryStructure":"\u0000"},{"value":0,"secondaryStructure":"\u0000"},{"value":0,"secondaryStructure":"\u0000"},{"value":0,"secondaryStructure":"\u0000"},{"value":0,"secondaryStructure":"\u0000"},{"value":0,"secondaryStructure":"\u0000"},{"value":0,"secondaryStructure":"\u0000"},{"value":0,"secondaryStructure":"\u0000"},{"value":0,"secondaryStructure":"\u0000"},{"value":0,"secondaryStructure":"\u0000"},{"value":0,"secondaryStructure":"\u0000"},{"value":0,"secondaryStructure":"\u0000"},{"value":0,"secondaryStructure":"\u0000"},{"value":0,"secondaryStructure":"\u0000"},{"value":0,"secondaryStructure":"\u0000"},{"value":0,"secondaryStructure":"\u0000"},{"value":0,"secondaryStructure":"\u0000"},{"value":0,"secondaryStructure":"\u0000"},{"value":0,"secondaryStructure":"\u0000"},{"value":0,"secondaryStructure":"\u0000"},{"value":0,"secondaryStructure":"\u0000"},{"value":0,"secondaryStructure":"\u0000"},{"value":0,"secondaryStructure":"\u0000"},{"value":0,"secondaryStructure":"\u0000"},{"value":0,"secondaryStructure":"\u0000"},{"value":0,"secondaryStructure":"\u0000"},{"value":0,"secondaryStructure":"\u0000"},{"value":0,"secondaryStructure":"\u0000"},{"value":0,"secondaryStructure":"\u0000"},{"value":0,"secondaryStructure":"\u0000"},{"value":0,"secondaryStructure":"\u0000"},{"value":0,"secondaryStructure":"\u0000"},{"value":0,"secondaryStructure":"\u0000"},{"value":0,"secondaryStructure":"\u0000"},{"value":0,"secondaryStructure":"\u0000"},{"value":0,"secondaryStructure":"\u0000"},{"value":0,"secondaryStructure":"\u0000"},{"value":0,"secondaryStructure":"\u0000"},{"value":0,"secondaryStructure":"\u0000"},{"value":0,"secondaryStructure":"\u0000"},{"value":0,"secondaryStructure":"\u0000"},{"value":0,"secondaryStructure":"\u0000"},{"value":0,"secondaryStructure":"\u0000"},{"value":0,"secondaryStructure":"\u0000"},{"value":0,"secondaryStructure":"\u0000"},{"value":0,"secondaryStructure":"\u0000"},{"value":0,"secondaryStructure":"\u0000"},{"value":0,"secondaryStructure":"\u0000"},{"value":0,"secondaryStructure":"\u0000"},{"value":0,"secondaryStructure":"\u0000"},{"value":0,"secondaryStructure":"\u0000"},{"value":0,"secondaryStructure":"\u0000"},{"value":0,"secondaryStructure":"\u0000"},{"value":0,"secondaryStructure":"\u0000"},{"value":0,"secondaryStructure":"\u0000"},{"value":0,"secondaryStructure":"\u0000"},{"value":0,"secondaryStructure":"\u0000"},{"value":0,"secondaryStructure":"\u0000"},{"value":0,"secondaryStructure":"\u0000"},{"value":0,"secondaryStructure":"\u0000"},{"value":0,"secondaryStructure":"\u0000"},{"value":0,"secondaryStructure":"\u0000"},{"value":0,"secondaryStructure":"\u0000"},{"value":0,"secondaryStructure":"\u0000"},{"value":0,"secondaryStructure":"\u0000"},{"value":0,"secondaryStructure":"\u0000"},{"value":0,"secondaryStructure":"\u0000"},{"value":0,"secondaryStructure":"\u0000"},{"value":0,"secondaryStructure":"\u0000"},{"value":0,"secondaryStructure":"\u0000"},{"value":0,"secondaryStructure":"\u0000"},{"value":0,"secondaryStructure":"\u0000"},{"value":0,"secondaryStructure":"\u0000"},{"value":0,"secondaryStructure":"\u0000"},{"value":0,"secondaryStructure":"\u0000"},{"value":0,"secondaryStructure":"\u0000"},{"value":0,"secondaryStructure":"\u0000"},{"value":0,"secondaryStructure":"\u0000"},{"value":0,"secondaryStructure":"\u0000"},{"value":0,"secondaryStructure":"\u0000"},{"value":0,"secondaryStructure":"\u0000"},{"value":0,"secondaryStructure":"\u0000"},{"value":0,"secondaryStructure":"\u0000"},{"value":0,"secondaryStructure":"\u0000"},{"value":0,"secondaryStructure":"\u0000"},{"value":0,"secondaryStructure":"\u0000"},{"value":0,"secondaryStructure":"\u0000"},{"value":0,"secondaryStructure":"\u0000"},{"value":0,"secondaryStructure":"\u0000"},{"value":0,"secondaryStructure":"\u0000"},{"value":0,"secondaryStructure":"\u0000"},{"value":0,"secondaryStructure":"\u0000"},{"value":0,"secondaryStructure":"\u0000"},{"description":"","displayCharacter":"Ketoreductase domain","value":0,"secondaryStructure":"H"},{"description":"","displayCharacter":"Ketoreductase domain","value":0,"secondaryStructure":"H"},{"description":"","displayCharacter":"Ketoreductase domain","value":0,"secondaryStructure":"H"},{"description":"","displayCharacter":"Ketoreductase domain","value":0,"secondaryStructure":"H"},{"description":"","displayCharacter":"Ketoreductase domain","value":0,"secondaryStructure":"H"},{"description":"","displayCharacter":"Ketoreductase domain","value":0,"secondaryStructure":"H"},{"description":"","displayCharacter":"Ketoreductase domain","value":0,"secondaryStructure":"H"},{"description":"","displayCharacter":"Ketoreductase domain","value":0,"secondaryStructure":"H"},{"description":"","displayCharacter":"Ketoreductase domain","value":0,"secondaryStructure":"H"},{"description":"","displayCharacter":"Ketoreductase domain","value":0,"secondaryStructure":"H"},{"description":"","displayCharacter":"Ketoreductase domain","value":0,"secondaryStructure":"H"},{"description":"","displayCharacter":"Ketoreductase domain","value":0,"secondaryStructure":"H"},{"description":"","displayCharacter":"Ketoreductase domain","value":0,"secondaryStructure":"H"},{"description":"","displayCharacter":"Ketoreductase domain","value":0,"secondaryStructure":"H"},{"description":"","displayCharacter":"Ketoreductase domain","value":0,"secondaryStructure":"H"},{"description":"","displayCharacter":"Ketoreductase domain","value":0,"secondaryStructure":"H"},{"description":"","displayCharacter":"Ketoreductase domain","value":0,"secondaryStructure":"H"},{"description":"","displayCharacter":"Ketoreductase domain","value":0,"secondaryStructure":"H"},{"description":"","displayCharacter":"Ketoreductase domain","value":0,"secondaryStructure":"H"},{"description":"","displayCharacter":"Ketoreductase domain","value":0,"secondaryStructure":"H"},{"description":"","displayCharacter":"Ketoreductase domain","value":0,"secondaryStructure":"H"},{"description":"","displayCharacter":"Ketoreductase domain","value":0,"secondaryStructure":"H"},{"description":"","displayCharacter":"Ketoreductase domain","value":0,"secondaryStructure":"H"},{"description":"","displayCharacter":"Ketoreductase domain","value":0,"secondaryStructure":"H"},{"description":"","displayCharacter":"Ketoreductase domain","value":0,"secondaryStructure":"H"},{"description":"","displayCharacter":"Ketoreductase domain","value":0,"secondaryStructure":"H"},{"description":"","displayCharacter":"Ketoreductase domain","value":0,"secondaryStructure":"H"},{"description":"","displayCharacter":"Ketoreductase domain","value":0,"secondaryStructure":"H"},{"description":"","displayCharacter":"Ketoreductase domain","value":0,"secondaryStructure":"H"},{"description":"","displayCharacter":"Ketoreductase domain","value":0,"secondaryStructure":"H"},{"description":"","displayCharacter":"Ketoreductase domain","value":0,"secondaryStructure":"H"},{"description":"","displayCharacter":"Ketoreductase domain","value":0,"secondaryStructure":"H"},{"description":"","displayCharacter":"Ketoreductase domain","value":0,"secondaryStructure":"H"},{"description":"","displayCharacter":"Ketoreductase domain","value":0,"secondaryStructure":"H"},{"description":"","displayCharacter":"Ketoreductase domain","value":0,"secondaryStructure":"H"},{"description":"","displayCharacter":"Ketoreductase domain","value":0,"secondaryStructure":"H"},{"description":"","displayCharacter":"Ketoreductase domain","value":0,"secondaryStructure":"H"},{"description":"","displayCharacter":"Ketoreductase domain","value":0,"secondaryStructure":"H"},{"description":"","displayCharacter":"Ketoreductase domain","value":0,"secondaryStructure":"H"},{"description":"","displayCharacter":"Ketoreductase domain","value":0,"secondaryStructure":"H"},{"description":"","displayCharacter":"Ketoreductase domain","value":0,"secondaryStructure":"H"},{"description":"","displayCharacter":"Ketoreductase domain","value":0,"secondaryStructure":"H"},{"description":"","displayCharacter":"Ketoreductase domain","value":0,"secondaryStructure":"H"},{"description":"","displayCharacter":"Ketoreductase domain","value":0,"secondaryStructure":"H"},{"description":"","displayCharacter":"Ketoreductase domain","value":0,"secondaryStructure":"H"},{"description":"","displayCharacter":"Ketoreductase domain","value":0,"secondaryStructure":"H"},{"description":"","displayCharacter":"Ketoreductase domain","value":0,"secondaryStructure":"H"},{"description":"","displayCharacter":"Ketoreductase domain","value":0,"secondaryStructure":"H"},{"description":"","displayCharacter":"Ketoreductase domain","value":0,"secondaryStructure":"H"},{"description":"","displayCharacter":"Ketoreductase domain","value":0,"secondaryStructure":"H"},{"description":"","displayCharacter":"Ketoreductase domain","value":0,"secondaryStructure":"H"},{"description":"","displayCharacter":"Ketoreductase domain","value":0,"secondaryStructure":"H"},{"description":"","displayCharacter":"Ketoreductase domain","value":0,"secondaryStructure":"H"},{"description":"","displayCharacter":"Ketoreductase domain","value":0,"secondaryStructure":"H"},{"description":"","displayCharacter":"Ketoreductase domain","value":0,"secondaryStructure":"H"},{"description":"","displayCharacter":"Ketoreductase domain","value":0,"secondaryStructure":"H"},{"description":"","displayCharacter":"Ketoreductase domain","value":0,"secondaryStructure":"H"},{"description":"","displayCharacter":"Ketoreductase domain","value":0,"secondaryStructure":"H"},{"description":"","displayCharacter":"Ketoreductase domain","value":0,"secondaryStructure":"H"},{"description":"","displayCharacter":"Ketoreductase domain","value":0,"secondaryStructure":"H"},{"description":"","displayCharacter":"Ketoreductase domain","value":0,"secondaryStructure":"H"},{"description":"","displayCharacter":"Ketoreductase domain","value":0,"secondaryStructure":"H"},{"description":"","displayCharacter":"Ketoreductase domain","value":0,"secondaryStructure":"H"},{"description":"","displayCharacter":"Ketoreductase domain","value":0,"secondaryStructure":"H"},{"description":"","displayCharacter":"Ketoreductase domain","value":0,"secondaryStructure":"H"},{"description":"","displayCharacter":"Ketoreductase domain","value":0,"secondaryStructure":"H"},{"description":"","displayCharacter":"Ketoreductase domain","value":0,"secondaryStructure":"H"},{"description":"","displayCharacter":"Ketoreductase domain","value":0,"secondaryStructure":"H"},{"description":"","displayCharacter":"Ketoreductase domain","value":0,"secondaryStructure":"H"},{"description":"","displayCharacter":"Ketoreductase domain","value":0,"secondaryStructure":"H"},{"description":"","displayCharacter":"Ketoreductase domain","value":0,"secondaryStructure":"H"},{"description":"","displayCharacter":"Ketoreductase domain","value":0,"secondaryStructure":"H"},{"description":"","displayCharacter":"Ketoreductase domain","value":0,"secondaryStructure":"H"},{"description":"","displayCharacter":"Ketoreductase domain","value":0,"secondaryStructure":"H"},{"description":"","displayCharacter":"Ketoreductase domain","value":0,"secondaryStructure":"H"},{"description":"","displayCharacter":"Ketoreductase domain","value":0,"secondaryStructure":"H"},{"description":"","displayCharacter":"Ketoreductase domain","value":0,"secondaryStructure":"H"},{"description":"","displayCharacter":"Ketoreductase domain","value":0,"secondaryStructure":"H"},{"description":"","displayCharacter":"Ketoreductase domain","value":0,"secondaryStructure":"H"},{"description":"","displayCharacter":"Ketoreductase domain","value":0,"secondaryStructure":"H"},{"description":"","displayCharacter":"Ketoreductase domain","value":0,"secondaryStructure":"H"},{"description":"","displayCharacter":"Ketoreductase domain","value":0,"secondaryStructure":"H"},{"description":"","displayCharacter":"Ketoreductase domain","value":0,"secondaryStructure":"H"},{"description":"","displayCharacter":"Ketoreductase domain","value":0,"secondaryStructure":"H"},{"description":"","displayCharacter":"Ketoreductase domain","value":0,"secondaryStructure":"H"},{"description":"","displayCharacter":"Ketoreductase domain","value":0,"secondaryStructure":"H"},{"description":"","displayCharacter":"Ketoreductase domain","value":0,"secondaryStructure":"H"},{"description":"","displayCharacter":"Ketoreductase domain","value":0,"secondaryStructure":"H"},{"description":"","displayCharacter":"Ketoreductase domain","value":0,"secondaryStructure":"H"},{"description":"","displayCharacter":"Ketoreductase domain","value":0,"secondaryStructure":"H"},{"description":"","displayCharacter":"Ketoreductase domain","value":0,"secondaryStructure":"H"},{"description":"","displayCharacter":"Ketoreductase domain","value":0,"secondaryStructure":"H"},{"description":"","displayCharacter":"Ketoreductase domain","value":0,"secondaryStructure":"H"},{"description":"","displayCharacter":"Ketoreductase domain","value":0,"secondaryStructure":"H"},{"description":"","displayCharacter":"Ketoreductase domain","value":0,"secondaryStructure":"H"},{"description":"","displayCharacter":"Ketoreductase domain","value":0,"secondaryStructure":"H"},{"description":"","displayCharacter":"Ketoreductase domain","value":0,"secondaryStructure":"H"},{"description":"","displayCharacter":"Ketoreductase domain","value":0,"secondaryStructure":"H"},{"description":"","displayCharacter":"Ketoreductase domain","value":0,"secondaryStructure":"H"},{"description":"","displayCharacter":"Ketoreductase domain","value":0,"secondaryStructure":"H"},{"description":"","displayCharacter":"Ketoreductase domain","value":0,"secondaryStructure":"H"},{"description":"","displayCharacter":"Ketoreductase domain","value":0,"secondaryStructure":"H"},{"description":"","displayCharacter":"Ketoreductase domain","value":0,"secondaryStructure":"H"},{"description":"","displayCharacter":"Ketoreductase domain","value":0,"secondaryStructure":"H"},{"description":"","displayCharacter":"Ketoreductase domain","value":0,"secondaryStructure":"H"},{"description":"","displayCharacter":"Ketoreductase domain","value":0,"secondaryStructure":"H"},{"description":"","displayCharacter":"Ketoreductase domain","value":0,"secondaryStructure":"H"},{"description":"","displayCharacter":"Ketoreductase domain","value":0,"secondaryStructure":"H"},{"description":"","displayCharacter":"Ketoreductase domain","value":0,"secondaryStructure":"H"},{"description":"","displayCharacter":"Ketoreductase domain","value":0,"secondaryStructure":"H"},{"description":"","displayCharacter":"Ketoreductase domain","value":0,"secondaryStructure":"H"},{"description":"","displayCharacter":"Ketoreductase domain","value":0,"secondaryStructure":"H"},{"description":"","displayCharacter":"Ketoreductase domain","value":0,"secondaryStructure":"H"},{"description":"","displayCharacter":"Ketoreductase domain","value":0,"secondaryStructure":"H"},{"description":"","displayCharacter":"Ketoreductase domain","value":0,"secondaryStructure":"H"},{"description":"","displayCharacter":"Ketoreductase domain","value":0,"secondaryStructure":"H"},{"description":"","displayCharacter":"Ketoreductase domain","value":0,"secondaryStructure":"H"},{"description":"","displayCharacter":"Ketoreductase domain","value":0,"secondaryStructure":"H"},{"description":"","displayCharacter":"Ketoreductase domain","value":0,"secondaryStructure":"H"},{"description":"","displayCharacter":"Ketoreductase domain","value":0,"secondaryStructure":"H"},{"description":"","displayCharacter":"Ketoreductase domain","value":0,"secondaryStructure":"H"},{"description":"","displayCharacter":"Ketoreductase domain","value":0,"secondaryStructure":"H"},{"description":"","displayCharacter":"Ketoreductase domain","value":0,"secondaryStructure":"H"},{"description":"","displayCharacter":"Ketoreductase domain","value":0,"secondaryStructure":"H"},{"description":"","displayCharacter":"Ketoreductase domain","value":0,"secondaryStructure":"H"},{"description":"","displayCharacter":"Ketoreductase domain","value":0,"secondaryStructure":"H"},{"description":"","displayCharacter":"Ketoreductase domain","value":0,"secondaryStructure":"H"},{"description":"","displayCharacter":"Ketoreductase domain","value":0,"secondaryStructure":"H"},{"description":"","displayCharacter":"Ketoreductase domain","value":0,"secondaryStructure":"H"},{"description":"","displayCharacter":"Ketoreductase domain","value":0,"secondaryStructure":"H"},{"description":"","displayCharacter":"Ketoreductase domain","value":0,"secondaryStructure":"H"},{"description":"","displayCharacter":"Ketoreductase domain","value":0,"secondaryStructure":"H"},{"description":"","displayCharacter":"Ketoreductase domain","value":0,"secondaryStructure":"H"},{"description":"","displayCharacter":"Ketoreductase domain","value":0,"secondaryStructure":"H"},{"description":"","displayCharacter":"Ketoreductase domain","value":0,"secondaryStructure":"H"},{"description":"","displayCharacter":"Ketoreductase domain","value":0,"secondaryStructure":"H"},{"description":"","displayCharacter":"Ketoreductase domain","value":0,"secondaryStructure":"H"},{"description":"","displayCharacter":"Ketoreductase domain","value":0,"secondaryStructure":"H"},{"description":"","displayCharacter":"Ketoreductase domain","value":0,"secondaryStructure":"H"},{"description":"","displayCharacter":"Ketoreductase domain","value":0,"secondaryStructure":"H"},{"description":"","displayCharacter":"Ketoreductase domain","value":0,"secondaryStructure":"H"},{"description":"","displayCharacter":"Ketoreductase domain","value":0,"secondaryStructure":"H"},{"description":"","displayCharacter":"Ketoreductase domain","value":0,"secondaryStructure":"H"},{"description":"","displayCharacter":"Ketoreductase domain","value":0,"secondaryStructure":"H"},{"description":"","displayCharacter":"Ketoreductase domain","value":0,"secondaryStructure":"H"},{"description":"","displayCharacter":"Ketoreductase domain","value":0,"secondaryStructure":"H"},{"description":"","displayCharacter":"Ketoreductase domain","value":0,"secondaryStructure":"H"},{"description":"","displayCharacter":"Ketoreductase domain","value":0,"secondaryStructure":"H"},{"description":"","displayCharacter":"Ketoreductase domain","value":0,"secondaryStructure":"H"},{"description":"","displayCharacter":"Ketoreductase domain","value":0,"secondaryStructure":"H"},{"description":"","displayCharacter":"Ketoreductase domain","value":0,"secondaryStructure":"H"},{"description":"","displayCharacter":"Ketoreductase domain","value":0,"secondaryStructure":"H"},{"description":"","displayCharacter":"Ketoreductase domain","value":0,"secondaryStructure":"H"},{"description":"","displayCharacter":"Ketoreductase domain","value":0,"secondaryStructure":"H"},{"description":"","displayCharacter":"Ketoreductase domain","value":0,"secondaryStructure":"H"},{"description":"","displayCharacter":"Ketoreductase domain","value":0,"secondaryStructure":"H"},{"description":"","displayCharacter":"Ketoreductase domain","value":0,"secondaryStructure":"H"},{"description":"","displayCharacter":"Ketoreductase domain","value":0,"secondaryStructure":"H"},{"description":"","displayCharacter":"Ketoreductase domain","value":0,"secondaryStructure":"H"},{"description":"","displayCharacter":"Ketoreductase domain","value":0,"secondaryStructure":"H"},{"description":"","displayCharacter":"Ketoreductase domain","value":0,"secondaryStructure":"H"},{"description":"","displayCharacter":"Ketoreductase domain","value":0,"secondaryStructure":"H"},{"description":"","displayCharacter":"Ketoreductase domain","value":0,"secondaryStructure":"H"},{"description":"","displayCharacter":"Ketoreductase domain","value":0,"secondaryStructure":"H"},{"description":"","displayCharacter":"Ketoreductase domain","value":0,"secondaryStructure":"H"},{"description":"","displayCharacter":"Ketoreductase domain","value":0,"secondaryStructure":"H"},{"description":"","displayCharacter":"Ketoreductase domain","value":0,"secondaryStructure":"H"},{"description":"","displayCharacter":"Ketoreductase domain","value":0,"secondaryStructure":"H"},{"description":"","displayCharacter":"Ketoreductase domain","value":0,"secondaryStructure":"H"},{"description":"","displayCharacter":"Ketoreductase domain","value":0,"secondaryStructure":"H"},{"description":"","displayCharacter":"Ketoreductase domain","value":0,"secondaryStructure":"H"},{"description":"","displayCharacter":"Ketoreductase domain","value":0,"secondaryStructure":"H"},{"description":"","displayCharacter":"Ketoreductase domain","value":0,"secondaryStructure":"H"},{"description":"","displayCharacter":"Ketoreductase domain","value":0,"secondaryStructure":"H"},{"description":"","displayCharacter":"Ketoreductase domain","value":0,"secondaryStructure":"H"},{"description":"","displayCharacter":"Ketoreductase domain","value":0,"secondaryStructure":"H"},{"description":"","displayCharacter":"Ketoreductase domain","value":0,"secondaryStructure":"H"},{"description":"","displayCharacter":"Ketoreductase domain","value":0,"secondaryStructure":"H"},{"description":"","displayCharacter":"Ketoreductase domain","value":0,"secondaryStructure":"H"},{"description":"","displayCharacter":"Ketoreductase domain","value":0,"secondaryStructure":"H"},{"value":0,"secondaryStructure":"\u0000"},{"value":0,"secondaryStructure":"\u0000"},{"value":0,"secondaryStructure":"\u0000"},{"value":0,"secondaryStructure":"\u0000"},{"value":0,"secondaryStructure":"\u0000"},{"value":0,"secondaryStructure":"\u0000"},{"value":0,"secondaryStructure":"\u0000"},{"value":0,"secondaryStructure":"\u0000"},{"value":0,"secondaryStructure":"\u0000"},{"value":0,"secondaryStructure":"\u0000"},{"value":0,"secondaryStructure":"\u0000"},{"value":0,"secondaryStructure":"\u0000"},{"value":0,"secondaryStructure":"\u0000"},{"value":0,"secondaryStructure":"\u0000"},{"value":0,"secondaryStructure":"\u0000"},{"value":0,"secondaryStructure":"\u0000"},{"value":0,"secondaryStructure":"\u0000"},{"value":0,"secondaryStructure":"\u0000"},{"value":0,"secondaryStructure":"\u0000"},{"value":0,"secondaryStructure":"\u0000"},{"value":0,"secondaryStructure":"\u0000"},{"value":0,"secondaryStructure":"\u0000"},{"value":0,"secondaryStructure":"\u0000"},{"value":0,"secondaryStructure":"\u0000"},{"value":0,"secondaryStructure":"\u0000"},{"value":0,"secondaryStructure":"\u0000"},{"value":0,"secondaryStructure":"\u0000"},{"value":0,"secondaryStructure":"\u0000"},{"value":0,"secondaryStructure":"\u0000"},{"value":0,"secondaryStructure":"\u0000"},{"value":0,"secondaryStructure":"\u0000"},{"value":0,"secondaryStructure":"\u0000"},{"value":0,"secondaryStructure":"\u0000"},{"value":0,"secondaryStructure":"\u0000"},{"value":0,"secondaryStructure":"\u0000"},{"value":0,"secondaryStructure":"\u0000"},{"value":0,"secondaryStructure":"\u0000"},{"value":0,"secondaryStructure":"\u0000"},{"value":0,"secondaryStructure":"\u0000"},{"value":0,"secondaryStructure":"\u0000"},{"value":0,"secondaryStructure":"\u0000"},{"value":0,"secondaryStructure":"\u0000"},{"value":0,"secondaryStructure":"\u0000"},{"value":0,"secondaryStructure":"\u0000"},{"value":0,"secondaryStructure":"\u0000"},{"value":0,"secondaryStructure":"\u0000"},{"value":0,"secondaryStructure":"\u0000"},{"value":0,"secondaryStructure":"\u0000"},{"value":0,"secondaryStructure":"\u0000"},{"value":0,"secondaryStructure":"\u0000"},{"value":0,"secondaryStructure":"\u0000"},{"value":0,"secondaryStructure":"\u0000"},{"value":0,"secondaryStructure":"\u0000"},{"value":0,"secondaryStructure":"\u0000"},{"value":0,"secondaryStructure":"\u0000"},{"value":0,"secondaryStructure":"\u0000"},{"value":0,"secondaryStructure":"\u0000"},{"value":0,"secondaryStructure":"\u0000"},{"value":0,"secondaryStructure":"\u0000"},{"value":0,"secondaryStructure":"\u0000"},{"value":0,"secondaryStructure":"\u0000"},{"value":0,"secondaryStructure":"\u0000"},{"value":0,"secondaryStructure":"\u0000"},{"value":0,"secondaryStructure":"\u0000"},{"value":0,"secondaryStructure":"\u0000"},{"value":0,"secondaryStructure":"\u0000"},{"value":0,"secondaryStructure":"\u0000"},{"value":0,"secondaryStructure":"\u0000"},{"value":0,"secondaryStructure":"\u0000"},{"value":0,"secondaryStructure":"\u0000"},{"value":0,"secondaryStructure":"\u0000"},{"value":0,"secondaryStructure":"\u0000"},{"value":0,"secondaryStructure":"\u0000"},{"value":0,"secondaryStructure":"\u0000"},{"value":0,"secondaryStructure":"\u0000"},{"value":0,"secondaryStructure":"\u0000"},{"value":0,"secondaryStructure":"\u0000"},{"value":0,"secondaryStructure":"\u0000"},{"value":0,"secondaryStructure":"\u0000"},{"value":0,"secondaryStructure":"\u0000"},{"value":0,"secondaryStructure":"\u0000"},{"value":0,"secondaryStructure":"\u0000"},{"value":0,"secondaryStructure":"\u0000"},{"value":0,"secondaryStructure":"\u0000"},{"value":0,"secondaryStructure":"\u0000"},{"value":0,"secondaryStructure":"\u0000"},{"value":0,"secondaryStructure":"\u0000"},{"description":"","displayCharacter":"ACP","value":0,"secondaryStructure":"H"},{"description":"","displayCharacter":"ACP","value":0,"secondaryStructure":"H"},{"description":"","displayCharacter":"ACP","value":0,"secondaryStructure":"H"},{"description":"","displayCharacter":"ACP","value":0,"secondaryStructure":"H"},{"description":"","displayCharacter":"ACP","value":0,"secondaryStructure":"H"},{"description":"","displayCharacter":"ACP","value":0,"secondaryStructure":"H"},{"description":"","displayCharacter":"ACP","value":0,"secondaryStructure":"H"},{"description":"","displayCharacter":"ACP","value":0,"secondaryStructure":"H"},{"description":"","displayCharacter":"ACP","value":0,"secondaryStructure":"H"},{"description":"","displayCharacter":"ACP","value":0,"secondaryStructure":"H"},{"description":"","displayCharacter":"ACP","value":0,"secondaryStructure":"H"},{"description":"","displayCharacter":"ACP","value":0,"secondaryStructure":"H"},{"description":"","displayCharacter":"ACP","value":0,"secondaryStructure":"H"},{"description":"","displayCharacter":"ACP","value":0,"secondaryStructure":"H"},{"description":"","displayCharacter":"ACP","value":0,"secondaryStructure":"H"},{"description":"","displayCharacter":"ACP","value":0,"secondaryStructure":"H"},{"description":"","displayCharacter":"ACP","value":0,"secondaryStructure":"H"},{"description":"","displayCharacter":"ACP","value":0,"secondaryStructure":"H"},{"description":"","displayCharacter":"ACP","value":0,"secondaryStructure":"H"},{"description":"","displayCharacter":"ACP","value":0,"secondaryStructure":"H"},{"description":"","displayCharacter":"ACP","value":0,"secondaryStructure":"H"},{"description":"","displayCharacter":"ACP","value":0,"secondaryStructure":"H"},{"description":"","displayCharacter":"ACP","value":0,"secondaryStructure":"H"},{"description":"","displayCharacter":"ACP","value":0,"secondaryStructure":"H"},{"description":"","displayCharacter":"ACP","value":0,"secondaryStructure":"H"},{"description":"","displayCharacter":"ACP","value":0,"secondaryStructure":"H"},{"description":"","displayCharacter":"ACP","value":0,"secondaryStructure":"H"},{"description":"","displayCharacter":"ACP","value":0,"secondaryStructure":"H"},{"description":"","displayCharacter":"ACP","value":0,"secondaryStructure":"H"},{"description":"","displayCharacter":"ACP","value":0,"secondaryStructure":"H"},{"description":"","displayCharacter":"ACP","value":0,"secondaryStructure":"H"},{"description":"","displayCharacter":"ACP","value":0,"secondaryStructure":"H"},{"description":"","displayCharacter":"ACP","value":0,"secondaryStructure":"H"},{"description":"","displayCharacter":"ACP","value":0,"secondaryStructure":"H"},{"description":"","displayCharacter":"ACP","value":0,"secondaryStructure":"H"},{"description":"","displayCharacter":"ACP","value":0,"secondaryStructure":"H"},{"description":"","displayCharacter":"ACP","value":0,"secondaryStructure":"H"},{"description":"","displayCharacter":"ACP","value":0,"secondaryStructure":"H"},{"description":"","displayCharacter":"ACP","value":0,"secondaryStructure":"H"},{"description":"","displayCharacter":"ACP","value":0,"secondaryStructure":"H"},{"description":"","displayCharacter":"ACP","value":0,"secondaryStructure":"H"},{"description":"","displayCharacter":"ACP","value":0,"secondaryStructure":"H"},{"description":"","displayCharacter":"ACP","value":0,"secondaryStructure":"H"},{"description":"","displayCharacter":"ACP","value":0,"secondaryStructure":"H"},{"description":"","displayCharacter":"ACP","value":0,"secondaryStructure":"H"},{"description":"","displayCharacter":"ACP","value":0,"secondaryStructure":"H"},{"description":"","displayCharacter":"ACP","value":0,"secondaryStructure":"H"},{"description":"","displayCharacter":"ACP","value":0,"secondaryStructure":"H"},{"description":"","displayCharacter":"ACP","value":0,"secondaryStructure":"H"},{"description":"","displayCharacter":"ACP","value":0,"secondaryStructure":"H"},{"description":"","displayCharacter":"ACP","value":0,"secondaryStructure":"H"},{"description":"","displayCharacter":"ACP","value":0,"secondaryStructure":"H"},{"description":"","displayCharacter":"ACP","value":0,"secondaryStructure":"H"},{"description":"","displayCharacter":"ACP","value":0,"secondaryStructure":"H"},{"description":"","displayCharacter":"ACP","value":0,"secondaryStructure":"H"},{"description":"","displayCharacter":"ACP","value":0,"secondaryStructure":"H"},{"description":"","displayCharacter":"ACP","value":0,"secondaryStructure":"H"},{"description":"","displayCharacter":"ACP","value":0,"secondaryStructure":"H"},{"description":"","displayCharacter":"ACP","value":0,"secondaryStructure":"H"},{"description":"","displayCharacter":"ACP","value":0,"secondaryStructure":"H"},{"description":"","displayCharacter":"ACP","value":0,"secondaryStructure":"H"},{"description":"","displayCharacter":"ACP","value":0,"secondaryStructure":"H"},{"description":"","displayCharacter":"ACP","value":0,"secondaryStructure":"H"},{"description":"","displayCharacter":"ACP","value":0,"secondaryStructure":"H"},{"description":"","displayCharacter":"ACP","value":0,"secondaryStructure":"H"},{"description":"","displayCharacter":"ACP","value":0,"secondaryStructure":"H"},{"description":"","displayCharacter":"ACP","value":0,"secondaryStructure":"H"},{"description":"","displayCharacter":"ACP","value":0,"secondaryStructure":"H"},{"description":"","displayCharacter":"ACP","value":0,"secondaryStructure":"H"},{"description":"","displayCharacter":"ACP","value":0,"secondaryStructure":"H"},{"description":"","displayCharacter":"ACP","value":0,"secondaryStructure":"H"},{"description":"","displayCharacter":"ACP","value":0,"secondaryStructure":"H"},{"description":"","displayCharacter":"ACP","value":0,"secondaryStructure":"H"},{"description":"","displayCharacter":"ACP","value":0,"secondaryStructure":"H"},{"description":"","displayCharacter":"ACP","value":0,"secondaryStructure":"H"},{"description":"","displayCharacter":"ACP","value":0,"secondaryStructure":"H"},{"description":"","displayCharacter":"ACP","value":0,"secondaryStructure":"H"},{"description":"","displayCharacter":"ACP","value":0,"secondaryStructure":"H"},{"description":"","displayCharacter":"ACP","value":0,"secondaryStructure":"H"},{"description":"","displayCharacter":"ACP","value":0,"secondaryStructure":"H"},{"description":"","displayCharacter":"ACP","value":0,"secondaryStructure":"H"},{"value":0,"secondaryStructure":"\u0000"},{"value":0,"secondaryStructure":"\u0000"},{"value":0,"secondaryStructure":"\u0000"},{"value":0,"secondaryStructure":"\u0000"},{"value":0,"secondaryStructure":"\u0000"},{"value":0,"secondaryStructure":"\u0000"},{"value":0,"secondaryStructure":"\u0000"},{"value":0,"secondaryStructure":"\u0000"},{"value":0,"secondaryStructure":"\u0000"},{"value":0,"secondaryStructure":"\u0000"},{"value":0,"secondaryStructure":"\u0000"},{"value":0,"secondaryStructure":"\u0000"},{"value":0,"secondaryStructure":"\u0000"},{"value":0,"secondaryStructure":"\u0000"}],"label":"Domains"}],"svid":"1.0","seqFeatures":[]}

  

xml version="1.0"?


nnHispSgcPKS(wt)gcPKS(Δ2)gcPKS(Δ2&C)cgPKS(wt)cgPKS(Δ2)cgPKS(Δ2&C)hsPKS(wt)hsPKS(Δ2)hsPKS(Δ2&C)DomainsConsensusnnHispSgcPKS(wt)gcPKS(Δ2)gcPKS(Δ2&C)cgPKS(wt)cgPKS(Δ2)cgPKS(Δ2&C)hsPKS(wt)hsPKS(Δ2)hsPKS(Δ2&C)DomainsConsensusnnHispSgcPKS(wt)gcPKS(Δ2)gcPKS(Δ2&C)cgPKS(wt)cgPKS(Δ2)cgPKS(Δ2&C)hsPKS(wt)hsPKS(Δ2)hsPKS(Δ2&C)DomainsConsensusnnHispSgcPKS(wt)gcPKS(Δ2)gcPKS(Δ2&C)cgPKS(wt)cgPKS(Δ2)cgPKS(Δ2&C)hsPKS(wt)hsPKS(Δ2)hsPKS(Δ2&C)DomainsConsensusnnHispSgcPKS(wt)gcPKS(Δ2)gcPKS(Δ2&C)cgPKS(wt)cgPKS(Δ2)cgPKS(Δ2&C)hsPKS(wt)hsPKS(Δ2)hsPKS(Δ2&C)DomainsConsensusnnHispSgcPKS(wt)gcPKS(Δ2)gcPKS(Δ2&C)cgPKS(wt)cgPKS(Δ2)cgPKS(Δ2&C)hsPKS(wt)hsPKS(Δ2)hsPKS(Δ2&C)DomainsConsensusnnHispSgcPKS(wt)gcPKS(Δ2)gcPKS(Δ2&C)cgPKS(wt)cgPKS(Δ2)cgPKS(Δ2&C)hsPKS(wt)hsPKS(Δ2)hsPKS(Δ2&C)DomainsConsensusnnHispSgcPKS(wt)gcPKS(Δ2)gcPKS(Δ2&C)cgPKS(wt)cgPKS(Δ2)cgPKS(Δ2&C)hsPKS(wt)hsPKS(Δ2)hsPKS(Δ2&C)DomainsConsensusnnHispSgcPKS(wt)gcPKS(Δ2)gcPKS(Δ2&C)cgPKS(wt)cgPKS(Δ2)cgPKS(Δ2&C)hsPKS(wt)hsPKS(Δ2)hsPKS(Δ2&C)DomainsConsensusnnHispSgcPKS(wt)gcPKS(Δ2)gcPKS(Δ2&C)cgPKS(wt)cgPKS(Δ2)cgPKS(Δ2&C)hsPKS(wt)hsPKS(Δ2)hsPKS(Δ2&C)DomainsConsensusnnHispSgcPKS(wt)gcPKS(Δ2)gcPKS(Δ2&C)cgPKS(wt)cgPKS(Δ2)cgPKS(Δ2&C)hsPKS(wt)hsPKS(Δ2)hsPKS(Δ2&C)DomainsConsensusnnHispSgcPKS(wt)gcPKS(Δ2)gcPKS(Δ2&C)cgPKS(wt)cgPKS(Δ2)cgPKS(Δ2&C)hsPKS(wt)hsPKS(Δ2)hsPKS(Δ2&C)DomainsConsensusnnHispSgcPKS(wt)gcPKS(Δ2)gcPKS(Δ2&C)cgPKS(wt)cgPKS(Δ2)cgPKS(Δ2&C)hsPKS(wt)hsPKS(Δ2)hsPKS(Δ2&C)DomainsConsensusnnHispSgcPKS(wt)gcPKS(Δ2)gcPKS(Δ2&C)cgPKS(wt)cgPKS(Δ2)cgPKS(Δ2&C)hsPKS(wt)hsPKS(Δ2)hsPKS(Δ2&C)DomainsConsensusnnHispSgcPKS(wt)gcPKS(Δ2)gcPKS(Δ2&C)cgPKS(wt)cgPKS(Δ2)cgPKS(Δ2&C)hsPKS(wt)hsPKS(Δ2)hsPKS(Δ2&C)DomainsConsensusnnHispSgcPKS(wt)gcPKS(Δ2)gcPKS(Δ2&C)cgPKS(wt)cgPKS(Δ2)cgPKS(Δ2&C)hsPKS(wt)hsPKS(Δ2)hsPKS(Δ2&C)DomainsConsensusnnHispSgcPKS(wt)gcPKS(Δ2)gcPKS(Δ2&C)cgPKS(wt)cgPKS(Δ2)cgPKS(Δ2&C)hsPKS(wt)hsPKS(Δ2)hsPKS(Δ2&C)DomainsConsensusnnHispSgcPKS(wt)gcPKS(Δ2)gcPKS(Δ2&C)cgPKS(wt)cgPKS(Δ2)cgPKS(Δ2&C)hsPKS(wt)hsPKS(Δ2)hsPKS(Δ2&C)DomainsConsensusnnHispSgcPKS(wt)gcPKS(Δ2)gcPKS(Δ2&C)cgPKS(wt)cgPKS(Δ2)cgPKS(Δ2&C)hsPKS(wt)hsPKS(Δ2)hsPKS(Δ2&C)DomainsConsensusnnHispSgcPKS(wt)gcPKS(Δ2)gcPKS(Δ2&C)cgPKS(wt)cgPKS(Δ2)cgPKS(Δ2&C)hsPKS(wt)hsPKS(Δ2)hsPKS(Δ2&C)DomainsConsensusnnHispSgcPKS(wt)gcPKS(Δ2)gcPKS(Δ2&C)cgPKS(wt)cgPKS(Δ2)cgPKS(Δ2&C)hsPKS(wt)hsPKS(Δ2)hsPKS(Δ2&C)DomainsConsensusnnHispSgcPKS(wt)gcPKS(Δ2)gcPKS(Δ2&C)cgPKS(wt)cgPKS(Δ2)cgPKS(Δ2&C)hsPKS(wt)hsPKS(Δ2)hsPKS(Δ2&C)DomainsConsensusnnHispSgcPKS(wt)gcPKS(Δ2)gcPKS(Δ2&C)cgPKS(wt)cgPKS(Δ2)cgPKS(Δ2&C)hsPKS(wt)hsPKS(Δ2)hsPKS(Δ2&C)DomainsConsensusnnHispSgcPKS(wt)gcPKS(Δ2)gcPKS(Δ2&C)cgPKS(wt)cgPKS(Δ2)cgPKS(Δ2&C)hsPKS(wt)hsPKS(Δ2)hsPKS(Δ2&C)DomainsConsensusnnHispSgcPKS(wt)gcPKS(Δ2)gcPKS(Δ2&C)cgPKS(wt)cgPKS(Δ2)cgPKS(Δ2&C)hsPKS(wt)hsPKS(Δ2)hsPKS(Δ2&C)DomainsConsensusnnHispSgcPKS(wt)gcPKS(Δ2)gcPKS(Δ2&C)cgPKS(wt)cgPKS(Δ2)cgPKS(Δ2&C)hsPKS(wt)hsPKS(Δ2)hsPKS(Δ2&C)DomainsConsensusMNSSKN--PPSTLLDVFLDTARNLDTASRNVLECGEHRWSYRELDTVSSALAQHLRYTVGLSPTVAVISENHPYILALMLAVWKLGGTFAPIDVHSPAELMDE------VKTLLDAFLSVARNPSTIEDPVLECGSEQWSYGDLDCISSGLALKIHQKCGLKPMVAVISENHPFVLAILLATWKLGGIFAPFDCHSPLEMMDE------VKTLLDAFLSVARNPSTIEDPVLECGSEQWSYGDLDCISSGLALKIHQKCGLKPMVAVISENHPFVLAILLATWKLGGIFAPFDCHSPLEMMDE------VKTLLDAFLSVARNPSTIEDPVLECGSEQWSYGDLDCISSGLALKIHQKCGLKPMVAVISENHPFVLAILLATWKLGGIFAPFDCHSPLEMMFCSISPPSPQTLLDSFLYAARNVETVENDVVECGYEKWSYGDLDVISTGLAIEIKETYGMKPTVATFSENHPYILAVMLATWKLGGICAPLDHHTPHELMFCSISPPSPQTLLDSFLYAARNVETVENDVVECGYEKWSYGDLDVISTGLAIEIKETYGMKPTVATFSENHPYILAVMLATWKLGGICAPLDHHTPHELMFCSISPPSPQTLLDSFLYAARNVETVENDVVECGYEKWSYGDLDVISTGLAIEIKETYGMKPTVATFSENHPYILAVMLATWKLGGICAPLDHHTPHELMAASLY--DKNTLLNAFLGVAHSAD-VDRNAVEYGNERWTYGDLDTVSTGLALEMHKKYGPKPVVAIVSENHPYTLAMLFAIWKLGGIAAPLDHNVPKDIMAASLY--DKNTLLNAFLGVAHSAD-VDRNAVEYGNERWTYGDLDTVSTGLALEMHKKYGPKPVVAIVSENHPYTLAMLFAIWKLGGIAAPLDHNVPKDIMAASLY--DKNTLLNAFLGVAHSAD-VDRNAVEYGNERWTYGDLDTVSTGLALEMHKKYGPKPVVAIVSENHPYTLAMLFAIWKLGGIAAPLDHNVPKDIAMP-binding domainM++S++PP+P+TLLDAFL+VARN+DTVERNVVECG+ERWSYGDLDTISTGLALEIH+KYGLKPTVAVISENHPYILA+LLATWKLGGIFAPLDHHSP+ELVAGMLNIVSPSCLVIPSSDVTNQTLACDLNIPVVAFHPHQSTIPELNKKYLTDS-QISP-DLPFPDPNRPALYLFTSSATSRSNLKCVPLTHTFILRNSLVEKMLENTEATCAVVPDFEDGLNGLLDKMQIPKISYS-KNTTITSLTQLYLAQVAEISPSLYPPPDLASLAAYIHTSSASSISNLKCVQLTHESIAYGCRVEKMLENTEATCAVVPDFEDGLNGLLDKMQIPKISYS-KNTTITSLTQLYLAQVAEISPSLYPPPDLASLAAYIHTSSASSISNLKCVQLTHESIAYGCRVEKMLENTEATCAVVPDFEDGLNGLLDKMQIPKISYS-KNTTITSLTQLYLAQVAEISPSLYPPPDLASLAAYIHTSSASSISNLKCVQLTHESIAYGCRVQHMIINIAPTFVVVPSSDEPIKQLLQGMNVHFMIFDVRTTSMTSLTQRFLNQSPDLSVEAFPLPSPSDIAFFLHTSSASSISNLKCVPLAHGSVFSGCKVQHMIINIAPTFVVVPSSDEPIKQLLQGMNVHFMIFDVRTTSMTSLTQRFLNQSPDLSVEAFPLPSPSDIAFFLHTSSASSISNLKCVPLAHGSVFSGCKVQHMIINIAPTFVVVPSSDEPIKQLLQGMNVHFMIFDVRTTSMTSLTQRFLNQSPDLSVEAFPLPSPSDIAFFLHTSSASSISNLKCVPLAHGSVFSGCKMERMLLNIGPTCVLVPATERVVQSIVEGISVACHAFNPKEMSITALMQKYLDLSPELTGPAFHLPNPDDIALYLHTSSASSVANVKCVPTTHASILGASAMERMLLNIGPTCVLVPATERVVQSIVEGISVACHAFNPKEMSITALMQKYLDLSPELTGPAFHLPNPDDIALYLHTSSASSVANVKCVPTTHASILGASAMERMLLNIGPTCVLVPATERVVQSIVEGISVACHAFNPKEMSITALMQKYLDLSPELTGPAFHLPNPDDIALYLHTSSASSVANVKCVPTTHASILGASAVE+ML+NI+PTCVVVPSSE+++Q+LL+GMNVP++AF+PK+TSITSLTQKYL+QSPELSP+AFPLPDP+DIALYLHTSSASSISNLKCVPLTH+SIL+GC+SKRAWCKRMRPETDFDGIRVLGWAPWSHVLAHMQDIGPLTLLNAGCYVFATTPSTYPTE-------------LKDDRDVISCAANAVMYKGVKSFACLPFSVIKWFHRAWPSVNFDKLRVLGIAPWSHIMALSYDFGAATFGTGGCYVFGVPPSGYPVGIEVGHVDDSVSGKAEEERDILDRLVDAAVKARPDVLVAVPWSVIKWFHRAWPSVNFDKLRVLGIAPWSHIMALSYDFGAATFGTGGCYVFGVPPSGYPVGIEVGHVDDSVSGKAEEERDILDRLVDAAVKARPDVLVAVPWSVIKWFHRAWPSVNFDKLRVLGIAPWSHIMALSYDFGAATFGTGGCYVFGVPPSGYPVGIEVGHVDDSVSGKAEEERDILDRLVDAAVKARPDVLVAVPWSRLTWWQKTWPDKNFMNLRVLGWSPWSHILGISHDIGGATFATAGCYLFGLIPSSYTSQQEL-------TDEYEGEFDIVSRLLNAVIRLRPDVFSAVPWSRLTWWQKTWPDKNFMNLRVLGWSPWSHILGISHDIGGATFATAGCYLFGLIPSSYTSQQEL-------TDEYEGEFDIVSRLLNAVIRLRPDVFSAVPWSRLTWWQKTWPDKNFMNLRVLGWSPWSHILGISHDIGGATFATAGCYLFGLIPSSYTSQQEL-------TDEYEGEFDIVSRLLNAVIRLRPDVFSAVPWARLAWWKRTWPAQQYTHLRVLGWSTWAHVIGLTNDLGAAMVLTAGCYIFAMPPASGAGGNA-----------AALYLDVCGQLLETAIIKQPTVFAGVPWARLAWWKRTWPAQQYTHLRVLGWSTWAHVIGLTNDLGAAMVLTAGCYIFAMPPASGAGGNA-----------AALYLDVCGQLLETAIIKQPTVFAGVPWARLAWWKRTWPAQQYTHLRVLGWSTWAHVIGLTNDLGAAMVLTAGCYIFAMPPASGAGGNA-----------AALYLDVCGQLLETAIIKQPTVFAGVPWSRLAWWKRTWP++NFD+LRVLGWSPWSHILGLS+DIGAATFLTAGCYVFG+PPSSYP+G+E+GHVDDSV+++AE+ERDI+SRLLNAAI+KRPDVFAAVPWVLGGLKALCESEPSVKAQLQVEERA---QLLKSLQHMKILECGGAMLEVSVASWAIENRIPISIGIGMTETGGALFAGPVQAI-----QTGFSSEDKFIEVLEGFKERYTRLLG------ANKEAEALRVKYALQALKCLGSGGAAMSAEMLSWIKELKINASSNIGMTELGGGLFHRKIDLLSTPDHDDGWSLEDCFFDVLEGFKERYTRLLG------ANKEAEALRVKYALQALKCLGSGGAAMSAEMLSWIKELKINASSNIGMTELGGGLFHRKIDLLSTPDHDDGWSLEDCFFDVLEGFKERYTRLLG------ANKEAEALRVKYALQALKCLGSGGAAMSAEMLSWIKELKINASSNIGMTELGGGLFHRKIDLLSTPDHDDGWSLEDCFFDVLEGFRDKWSRETV------ADKKR---VMQDVLEKMKVFGCGGAALSKEIVLWAKDMNIPVTVDIGMTELGRPLFYSKADDFD----LLGWSMKDCLIPVLEGFRDKWSRETV------ADKKR---VMQDVLEKMKVFGCGGAALSKEIVLWAKDMNIPVTVDIGMTELGRPLFYSKADDFD----LLGWSMKDCLIPVLEGFRDKWSRETV------ADKKR---VMQDVLEKMKVFGCGGAALSKEIVLWAKDMNIPVTVDIGMTELGRPLFYSKADDFD----LLGWSMKDCLIPVLEGFMRNYKQEAD------AARKQ---AIQDAVKRLKVFGSGGASTNAECIEWAIQMAIPLVLDIGMTEVGGPLFHSTIGG------PEGWLSEDCMLPVLEGFMRNYKQEAD------AARKQ---AIQDAVKRLKVFGSGGASTNAECIEWAIQMAIPLVLDIGMTEVGGPLFHSTIGG------PEGWLSEDCMLPVLEGFMRNYKQEAD------AARKQ---AIQDAVKRLKVFGSGGASTNAECIEWAIQMAIPLVLDIGMTEVGGPLFHSTIGG------PEGWLSEDCMLPVLEGFK++Y+RE++VKAQLQA+KKAEAL++QDALQ+LKVFGSGGAALSAE++SWAKEM+IP+S+DIGMTELGGPLFHSKID+++TPDH++GWSSEDCFIPDATYLLVKDDYESHAEED--INEGELVVKSRMLPRGYLGYNDPSFSVDDAGWVTFKTGDRYSVTPDGKFSWLGRNTDFIQMTSGETLDPRPIESLLCESSDVQLVLVDEDG----TEN--DQEGELIITTRHISRGYLKYDNSAFSFLPDGSTTFRTGDVYERKSDGRIVWKGRKDDYIQTASGETLDPRPIEKALSACEDVQLVLVDEDG----TEN--DQEGELIITTRHISRGYLKYDNSAFSFLPDGSTTFRTGDVYERKSDGRIVWKGRKDDYIQTASGETLDPRPIEKALSACEDVQLVLVDEDG----TEN--DQEGELIITTRHISRGYLKYDNSAFSFLPDGSTTFRTGDVYERKSDGRIVWKGRKDDYIQTASGETLDPRPIEKALSACEDAELRLVNENC----DDDDDLEEGELVITSGAISQGYLKFDNLAFTKAPDGRTTFRTGDVYTMTADDHLMWIGRKEDYIQMVSGETLDPRPIERALNTSSDAELRLVNENC----DDDDDLEEGELVITSGAISQGYLKFDNLAFTKAPDGRTTFRTGDVYTMTADDHLMWIGRKEDYIQMVSGETLDPRPIERALNTSSDAELRLVNENC----DDDDDLEEGELVITSGAISQGYLKFDNLAFTKAPDGRTTFRTGDVYTMTADDHLMWIGRKEDYIQMVSGETLDPRPIERALNTSSGAQLKLIDDSG----AEV--STEGELVVRAQNVTRGYRHYDNSSFTLENDGTVSFKTGDVYAFVGDQRLVWKGRKEDYIQMSSGESLDPRVVEAVLDKCPGAQLKLIDDSG----AEV--STEGELVVRAQNVTRGYRHYDNSSFTLENDGTVSFKTGDVYAFVGDQRLVWKGRKEDYIQMSSGESLDPRVVEAVLDKCPGAQLKLIDDSG----AEV--STEGELVVRAQNVTRGYRHYDNSSFTLENDGTVSFKTGDVYAFVGDQRLVWKGRKEDYIQMSSGESLDPRVVEAVLDKCPDAQL+LVDEDGESHA+EDDD++EGELVITSR+ISRGYLKYDNSAFT++PDG+TTFRTGDVY++T+DGRLVWKGRKEDYIQM+SGETLDPRPIE+AL++CSLISRACVIGDKFLNGPATAVCAIIELEPTT-VEKGQ---AHSRDIARIFAPINRDLPPPLRIAWSHVLVLQPSEKIPMTKKGTIFRKKIEQVFGSALGGSGVLHCCVVGNNFMRKASDAICILIEPAVTADGDSVTLTTTEIAQITKTLAAMNRGLLPPLRIPWSRVVVLEKGMRIPYTRKGMIFRKKLESLFGDVVSHLGVLHCCVVGNNFMRKASDAICILIEPAVTADGDSVTLTTTEIAQITKTLAAMNRGLLPPLRIPWSRVVVLEKGMRIPYTRKGMIFRKKLESLFGDVVSHLGVLHCCVVGNNFMRKASDAICILIEPAVTADGDSVTLTTTEIAQITKTLAAMNRGLLPPLRIPWSRVVVLEKGMRIPYTRKGMIFRKKLESLFGDVVSHLAISHSCVIGNHFLGRAAGFICVLIKPANNKAIQKTPSNAIITSEITRAVASVNRTLPPPLRIAWSRVLILDEGQEVPYTRKGTIFRKKLEGMFGGQVAGLAISHSCVIGNHFLGRAAGFICVLIKPANNKAIQKTPSNAIITSEITRAVASVNRTLPPPLRIAWSRVLILDEGQEVPYTRKGTIFRKKLEGMFGGQVAGLAISHSCVIGNHFLGRAAGFICVLIKPANNKAIQKTPSNAIITSEITRAVASVNRTLPPPLRIAWSRVLILDEGQEVPYTRKGTIFRKKLEGMFGGQVAGLAIARSCVVGNNFLKTSSQVVCAIVQPAKNT----------STTEITRAISVANRSLAPPLRISWSRVLVLSEGQEVPITKKGAIFRKKLEELFGAQLGALAIARSCVVGNNFLKTSSQVVCAIVQPAKNT----------STTEITRAISVANRSLAPPLRISWSRVLVLSEGQEVPITKKGAIFRKKLEELFGAQLGALAIARSCVVGNNFLKTSSQVVCAIVQPAKNT----------STTEITRAISVANRSLAPPLRISWSRVLVLSEGQEVPITKKGAIFRKKLEELFGAQLGALAISHSCVVGNNFL++AS+AICALIEPA+NT+++K+++++++T+EITRA+A++NR+LPPPLRIAWSRVLVL+EGQEVPYTRKGTIFRKKLE+LFG+QVGGLSG-----------------DNS-----QATTDASVVRRDE-LSNTVKHIISRVLGVSDDELL--WTLSFAELGMTSALATRIANELNEVLVGVNLPINACLEKDNLGHLALNGEINYEEDKK----PAKPSAPGIWKTED-VKSMTVVTIASILGVGTEALRAAPDTTFAEFGMDSNMAVRIVNELNSTF-SLHLPLNACLEKDNLGHLALNGEINYEEDKK----PAKPSAPGIWKTED-VKSMTVVTIASILGVGTEALRAAPDTTFAEFGMDSNMAVRIVNELNSTF-SLHLPLNACLEKDNLGHLALNGEINYEEDKK----PAKPSAPGIWKTED-VKSMTVVTIASILGVGTEALRAAPDTTFAEFGMDSNMAVRIVNELNSTF-SLHLPLNACLENGDV-------QDNQEKLAKVSDYHQEGTASSKWTKVD-VTDMVLKTVAGALQISIAVLSMHSDSSFIEFGMDSNMAVRIVNELNHLF-KLQLPLNTCLENGDV-------QDNQEKLAKVSDYHQEGTASSKWTKVD-VTDMVLKTVAGALQISIAVLSMHSDSSFIEFGMDSNMAVRIVNELNHLF-KLQLPLNTCLENGDV-------QDNQEKLAKVSDYHQEGTASSKWTKVD-VTDMVLKTVAGALQISIAVLSMHSDSSFIEFGMDSNMAVRIVNELNHLF-KLQLPLNTCLSRPEV-------DIASRAKTK----PSASSSRAQGKTRDQIASIVSNIVLQTLRISEETMDDNSQATFAELGMDSAMSTLIVNKLNRQL-DMSLPLNTCLSRPEV-------DIASRAKTK----PSASSSRAQGKTRDQIASIVSNIVLQTLRISEETMDDNSQATFAELGMDSAMSTLIVNKLNRQL-DMSLPLNTCLSRPEV-------DIASRAKTK----PSASSSRAQGKTRDQIASIVSNIVLQTLRISEETMDDNSQATFAELGMDSAMSTLIVNKLNRQL-DMSLPLNTCACPLE+++VGHLALNG+IN+E+D+KVSDYPQA+SA+S+WKT+DQV+SMV++TVA++LGIS+E+L+++SD+TFAEFGMDSNMAVRIVNELN++FV+L+LPLNTCYIHVDLPSLSNAVYAKLAHLKLPDRTPEPRKAPVENPGGKEIVIVGQAFRLPGSINDVASLRDAFLARQASSIITEIPPDRWDHASFYP----------KHTSVDLQSMCEAIMVELGIKGEQQASSGLCDQPTTTNVKEEVVIVGQALRLPGDINTPDAFWEALINKR-NDIMIPVPPDRWDHASFYRSPTSPEPPQIGHTSVDLQSMCEAIMVELGIKGEQQASSGLCDQPTTTNVKEEVVIVGQALRLPGDINTPDAFWEALINKR-NDIMIPVPPDRWDHASFYRSPTSPEPPQIGHTSVDLQSMCEAIMVELGIKGEQQASSGLCDQPTTTNVKEEVVIVGQALRLPGDINTPDAFWEALINKR-NDIMIPVPPDRWDHASFYRSPTSPEPPQIGHTYLDLVSLSGAVLTELGMNEKVTIEV-STTNPPTVQSHEEVVIVGQALRLPGDINTPESFWQALVDKR--DIMTPIPQDRWDQASFYRSPSSTAPPQDCHTYLDLVSLSGAVLTELGMNEKVTIEV-STTNPPTVQSHEEVVIVGQALRLPGDINTPESFWQALVDKR--DIMTPIPQDRWDQASFYRSPSSTAPPQDCHTYLDLVSLSGAVLTELGMNEKVTIEV-STTNPPTVQSHEEVVIVGQALRLPGDINTPESFWQALVDKR--DIMTPIPQDRWDQASFYRSPSSTAPPQDCHTHIDLVSLTNAILSDLGIDASSAKARPSTRVAPPAHEKEEIVIVGQAVRLPGDINTPDSFWRALIDKR-EDIITAVPASRWDHASFYRAPDSKEPPAPCHTHIDLVSLTNAILSDLGIDASSAKARPSTRVAPPAHEKEEIVIVGQAVRLPGDINTPDSFWRALIDKR-EDIITAVPASRWDHASFYRAPDSKEPPAPCHTHIDLVSLTNAILSDLGIDASSAKARPSTRVAPPAHEKEEIVIVGQAVRLPGDINTPDSFWRALIDKR-EDIITAVPASRWDHASFYRAPDSKEPPAPCKetosynthase domainHTHVDLVSLSNAIL+ELGI+++++++++ST++PPT+++KEEVVIVGQALRLPGDINTPDSFW+ALIDKRA+DIMTPVPPDRWDHASFYRSP+S+EPPQ+CDIRFNKAGLVDIANYDHSFFGLTATEALYLSPTMRLALEVSFEALENANIPVSQLKGSQTAVYVAT-TDDGFETLLNAEAGYDAYTRFYGTGRAASTASGDINFEKAGFLDVAHFDNAFFGISTPEAFFVSPSVRLTLETAFEALENANIPISKVKGTSMGAFVAAGLNEGYTHVLFTSLGWEAYKRTFGTGTASSTACGDINFEKAGFLDVAHFDNAFFGISTPEAFFVSPSVRLTLETAFEALENANIPISKVKGTSMGAFVAAGLNEGYTHVLFTSLGWEAYKRTFGTGTASSTACGDINFEKAGFLDVAHFDNAFFGISTPEAFFVSPSVRLTLETAFEALENANIPISKVKGTSMGAFVAAGLNEGYTHVLFTSLGWEAYKRTFGTGTASSTACGDITFEKAGFIQVESFDNSFFGISTPEAFYVSPTIRLTLETAFEALENANIPVSRVKGTNMGIFVAAGLDGGYQQLLYYDQGFGAYTRFFGTGIATSTACGDITFEKAGFIQVESFDNSFFGISTPEAFYVSPTIRLTLETAFEALENANIPVSRVKGTNMGIFVAAGLDGGYQQLLYYDQGFGAYTRFFGTGIATSTACGDITFEKAGFIQVESFDNSFFGISTPEAFYVSPTIRLTLETAFEALENANIPVSRVKGTNMGIFVAAGLDGGYQQLLYYDQGFGAYTRFFGTGIATSTACGDITLEKAGFVDSYSFDHAFFGISSAEAFHVSPNIRLSMEVAFEALENANIPPSKVKGSNMAVFVAASMDEGYIKLLFADKGWGAYTRFYGTGVATSTACGDITLEKAGFVDSYSFDHAFFGISSAEAFHVSPNIRLSMEVAFEALENANIPPSKVKGSNMAVFVAASMDEGYIKLLFADKGWGAYTRFYGTGVATSTACGDITLEKAGFVDSYSFDHAFFGISSAEAFHVSPNIRLSMEVAFEALENANIPPSKVKGSNMAVFVAASMDEGYIKLLFADKGWGAYTRFYGTGVATSTACGDITFEKAGFVDVASFDNAFFGISTPEAFYVSPTIRLTLETAFEALENANIPVSKVKGTNMGVFVAAGLDEGY++LLFAD+GWGAYTRFFGTG+ATSTACGRISYLLDVHGPSITVDTACSGGAVCIDQAIDYLQSSSAADTAIICASNTHCWPGSFMFLSAQGMVSSGGRCATFTTDADGYVPSEGAVAFILKTREAAMRRLSYLLDIHGPSMTTDTACSSGLVAFDQAVKYIQSGG-GESAMVSAASTALWPGSFGFLSANKMASVNSRCATFTTEADGYVPSEGCVAFVLKSKTAALRRLSYLLDIHGPSMTTDTACSSGLVAFDQAVKYIQSGG-GESAMVSAASTALWPGSFGFLSANKMASVNSRCATFTTEADGYVPSEGCVAFVLKSKTAALRRLSYLLDIHGPSMTTDTACSSGLVAFDQAVKYIQSGG-GESAMVSAASTALWPGSFGFLSANKMASVNSRCATFTTEADGYVPSEGCVAFVLKSKTAALRRLSYLLDVHGPSITSDTACSSGLVVFDQAVKYLQSGD-GESAIVCAVNTNLWPGSFGFLSAQKMASPHSRCATFSSEADGYVSSEGAVAFILKTRSAALRRLSYLLDVHGPSITSDTACSSGLVVFDQAVKYLQSGD-GESAIVCAVNTNLWPGSFGFLSAQKMASPHSRCATFSSEADGYVSSEGAVAFILKTRSAALRRLSYLLDVHGPSITSDTACSSGLVVFDQAVKYLQSGD-GESAIVCAVNTNLWPGSFGFLSAQKMASPHSRCATFSSEADGYVSSEGAVAFILKTRSAALRRLSYLLDVHGPSITIDTACSSGLIAFDQAVQYLQSGQ-GESAIVCGANTHAWPGTLGFLSAQKMTSSNSRCATFTNMADGYVPSEAAAGLIMKTKSAALRRLSYLLDVHGPSITIDTACSSGLIAFDQAVQYLQSGQ-GESAIVCGANTHAWPGTLGFLSAQKMTSSNSRCATFTNMADGYVPSEAAAGLIMKTKSAALRRLSYLLDVHGPSITIDTACSSGLIAFDQAVQYLQSGQ-GESAIVCGANTHAWPGTLGFLSAQKMTSSNSRCATFTNMADGYVPSEAAAGLIMKTKSAALRRLSYLLDVHGPSIT+DTACSSGLVAFDQAVKYLQSG+AGESAIVCAANTHLWPGSFGFLSAQKMASSNSRCATFTTEADGYVPSEGAVAFILKTKSAALRDKDTILATIKATQISHNGRSQGLVAPNVNSQADLHRSLLQKAGLSPADIHFIEAHGTGTSLGDLSEIQAINDAYTSSQPRTAGPLIVSASKTVIGHTEPADGDNILAIVKSTEVMHGGKSQGLVSPNVKTQIALQRSLLVQAGLQPSEIAFLEAHGTGTSLGDLIEIQGINEVFKYSHT--EDPLILGASKSCIGHTEMADGDNILAIVKSTEVMHGGKSQGLVSPNVKTQIALQRSLLVQAGLQPSEIAFLEAHGTGTSLGDLIEIQGINEVFKYSHT--EDPLILGASKSCIGHTEMADGDNILAIVKSTEVMHGGKSQGLVSPNVKTQIALQRSLLVQAGLQPSEIAFLEAHGTGTSLGDLIEIQGINEVFKYSHT--EDPLILGASKSCIGHTEMADGDSILAVVKSTDIKHGGRSQGLVSPNVNAQIALQSSLLEKAGLKPSEIDFVEAHGTGTSLGDLIEIQGINQVFQRSHS--ETPLIVGAAKSCIGHTEVADGDSILAVVKSTDIKHGGRSQGLVSPNVNAQIALQSSLLEKAGLKPSEIDFVEAHGTGTSLGDLIEIQGINQVFQRSHS--ETPLIVGAAKSCIGHTEVADGDSILAVVKSTDIKHGGRSQGLVSPNVNAQIALQSSLLEKAGLKPSEIDFVEAHGTGTSLGDLIEIQGINQVFQRSHS--ETPLIVGAAKSCIGHTEVADGDRIIGVVRSTDVQHDGRSQGLVAPNVKAQIAMQIALLEKAQLSPAQIDFIEAHGTGTSLGDLIEIQGINEVFEGSHG-ADKPLVVGAAKSCVGHAELVDGDRIIGVVRSTDVQHDGRSQGLVAPNVKAQIAMQIALLEKAQLSPAQIDFIEAHGTGTSLGDLIEIQGINEVFEGSHG-ADKPLVVGAAKSCVGHAELVDGDRIIGVVRSTDVQHDGRSQGLVAPNVKAQIAMQIALLEKAQLSPAQIDFIEAHGTGTSLGDLIEIQGINEVFEGSHG-ADKPLVVGAAKSCVGHAELVDGD+ILAVVKSTDV+HGGRSQGLVSPNVKAQIALQRSLLEKAGLSPSEIDFIEAHGTGTSLGDLIEIQGINEVF++SH+RAE+PLIVGAAKSCIGHTE+AGPLVGMLSVLNSFKEGAVPGLAHLTADNLNPALDCSSVPLLIPYQPVHLA---APKPHRAAVMSYGFSGTLGGIVLEAPDE-----E---RLEEEPPNDKSGLVGLLSAIASLKYGVVPGLVHLNEHNLNPSIDCDMVPLHIPHEVAPLP-RMPETPSRGLILSNGFAGTLAGAIIEGPGNAL-SGDLQKKQFSALNDQISGLVGLLSAIASLKYGVVPGLVHLNEHNLNPSIDCDMVPLHIPHEVAPLP-RMPETPSRGLILSNGFAGTLAGAIIEGPGNAL-SGDLQKKQFSALNDQISGLVGLLSAIASLKYGVVPGLVHLNEHNLNPSIDCDMVPLHIPHEVAPLP-RMPETPSRGLILSNGFAGTLAGAIIEGPGNAL-SGDLQKKQFSALNDQIAGLVGVLKAIASFRHSAIPGLMHLTAENMNPSIDCGIIPMHIPYELFPLPPKENNTPYRSVVLANGFAGTIAGVILEDPKHVIHSADLNSINEAETSEDFAGLVGVLKAIASFRHSAIPGLMHLTAENMNPSIDCGIIPMHIPYELFPLPPKENNTPYRSVVLANGFAGTIAGVILEDPKHVIHSADLNSINEAETSEDFAGLVGVLKAIASFRHSAIPGLMHLTAENMNPSIDCGIIPMHIPYELFPLPPKENNTPYRSVVLANGFAGTIAGVILEDPKHVIHSADLNSINEAETSEDFAGLIGVVKTLGSFAKGSVPGLVQLTADNMNPNIDCSVVPLHIPIEPTVLK-TEDNLPLRALILSNGFAGSIAGTILEAPT-----EDMQPKASANIPETMAGLIGVVKTLGSFAKGSVPGLVQLTADNMNPNIDCSVVPLHIPIEPTVLK-TEDNLPLRALILSNGFAGSIAGTILEAPT-----EDMQPKASANIPETMAGLIGVVKTLGSFAKGSVPGLVQLTADNMNPNIDCSVVPLHIPIEPTVLK-TEDNLPLRALILSNGFAGSIAGTILEAPT-----EDMQPKASANIPETMAGLVGVLKAIASFK+GAVPGLVHLTADNMNPSIDCS+VPLHIPYEP+PLPP+E+NTP+RALILSNGFAGTIAG+ILEAP++++HS+DLQ+K+EAE+PED+PMLFVVSAKTHTALIEYLGRYLEFLLQANPQDFCDICYTSCVGREHYRYRFACVANDMEDLIGQLQKRLGSKVP--PKPSY-KRGALAFAFSGQGTQFRGPMPFVVSAKTPERLCAYMEKYIAFCRKSTPSDFVNLCFTTCVGREHYRYRFACVAHNLPELIATLESRLQELRYNAQAIGNVAGPRVAFAFPGQGSQFQGPMPFVVSAKTPERLCAYMEKYIAFCRKSTPSDFVNLCFTTCVGREHYRYRFACVAHNLPELIATLESRLQELRYNAQAIGNVAGPRVAFAFPGQGSQFQGPMPFVVSAKTPERLCAYMEKYIAFCRKSTPSDFVNLCFTTCVGREHYRYRFACVAHNLPELIATLESRLQELRYNAQAIGNVAGPRVAFAFPGQGSQFQGPMLFVVSAKSAEALTQYLWKYLDFCRTSSTSDFRSICYTTCLGREHYRYRFACVVGSMGSLIKVLENRLRSTSS--PVASNPAACRIAFAFPGQGSHYQAPMLFVVSAKSAEALTQYLWKYLDFCRTSSTSDFRSICYTTCLGREHYRYRFACVVGSMGSLIKVLENRLRSTSS--PVASNPAACRIAFAFPGQGSHYQAPMLFVVSAKSAEALTQYLWKYLDFCRTSSTSDFRSICYTTCLGREHYRYRFACVVGSMGSLIKVLENRLRSTSS--PVASNPAACRIAFAFPGQGSHYQAPMTFVVSGKSQDALNEYLSLYLDFCLDADSSLFHAICYTTCIGREHYRYRFACVVNNMQDLIARLEDRLQNTSS--TSAGG-NARRILLGFPGQGSQYQGPMTFVVSGKSQDALNEYLSLYLDFCLDADSSLFHAICYTTCIGREHYRYRFACVVNNMQDLIARLEDRLQNTSS--TSAGG-NARRILLGFPGQGSQYQGPMTFVVSGKSQDALNEYLSLYLDFCLDADSSLFHAICYTTCIGREHYRYRFACVVNNMQDLIARLEDRLQNTSS--TSAGG-NARRILLGFPGQGSQYQGAcyltransferase domainPMLFVVSAKS+EAL+EYL+KYLDFCR+S+PSDF++ICYTTCVGREHYRYRFACVVNNM+DLIA+LE+RLQSTSSNAP+AGN+AA+RIAFAFPGQGSQYQGMATELAKAYSGFRKIVSDLAKRASELSGHAIDRFLLAYDIGAENVAPDSEADQICIFVYQCSVLRWLQTMGIRPSAVIGHSLGEISASVAAGALSLDSALMASELAEHFPDFKEIISALSVEASQVSGYDILSFLLDKDSPCEFLVNEGRMGQIGIFVFQSSLASWLRSLGIEPFAVLGHSLGEIAATVTAGAMDYAFALMASELAEHFPDFKEIISALSVEASQVSGYDILSFLLDKDSPCEFLVNEGRMGQIGIFVFQSSLASWLRSLGIEPFAVLGHSLGEIAATVTAGAMDYAFALMASELAEHFPDFKEIISALSVEASQVSGYDILSFLLDKDSPCEFLVNEGRMGQIGIFVFQSSLASWLRSLGIEPFAVLGHSLGEIAATVTAGAMDYAFALMASDLVTRYPGFKDILDSAASTASTLSGYPISSFLVDAKTSCDLAIDNSQVAQICIFVYQYSICTWLKQLGIEPRAVLGHSLGEIAAAVIGGALPYEIGLMASDLVTRYPGFKDILDSAASTASTLSGYPISSFLVDAKTSCDLAIDNSQVAQICIFVYQYSICTWLKQLGIEPRAVLGHSLGEIAAAVIGGALPYEIGLMASDLVTRYPGFKDILDSAASTASTLSGYPISSFLVDAKTSCDLAIDNSQVAQICIFVYQYSICTWLKQLGIEPRAVLGHSLGEIAAAVIGGALPYEIGLMGRYLANQYSGFRTIITEAANKAAGLTGYPILPYLLDESAPKGLTIDHSEVAQVCIFIFQYSVATWLESIGIHAHAVLGHSLGEIAAAVIARTFTLEIGLMGRYLANQYSGFRTIITEAANKAAGLTGYPILPYLLDESAPKGLTIDHSEVAQVCIFIFQYSVATWLESIGIHAHAVLGHSLGEIAAAVIARTFTLEIGLMGRYLANQYSGFRTIITEAANKAAGLTGYPILPYLLDESAPKGLTIDHSEVAQVCIFIFQYSVATWLESIGIHAHAVLGHSLGEIAAAVIARTFTLEIGLMASELA++YPGFK+IIS+AA++AS+LSGYPILSFLLD+D+PCEL+ID+SEVAQICIFVFQYSVATWL+SLGIEP+AVLGHSLGEIAAAVIAGAL+YEIGLDLVISRARLLRSSTNAPAGMAAMSASQDEVVELIGKLDLDKANSLSVSVINGPQNTVVSGSSAAIESIVALAKGRKIKASALNINQAFHSPYVDSAVPGLKFVIRRAEILCPEFAQSAGMALIAASEETILQRLHELGLED--HLAIAVCNGPNSHAVSGNLLAIDSLVTDAKAQGIRATKLNVTQGFHSPSIYPYLPVLKFVIRRAEILCPEFAQSAGMALIAASEETILQRLHELGLED--HLAIAVCNGPNSHAVSGNLLAIDSLVTDAKAQGIRATKLNVTQGFHSPSIYPYLPVLKFVIRRAEILCPEFAQSAGMALIAASEETILQRLHELGLED--HLAIAVCNGPNSHAVSGNLLAIDSLVTDAKAQGIRATKLNVTQGFHSPSIYPYLPVLNLVVTRARLLQCDPTHPGGMAIVGASQERILYIIHKLGLDD--RLVIAVYNDPENHVISGEIKAIDTFLSTANIWGFRGTKINVDQG--APCISSALPALNLVVTRARLLQCDPTHPGGMAIVGASQERILYIIHKLGLDD--RLVIAVYNDPENHVISGEIKAIDTFLSTANIWGFRGTKINVDQG--APCISSALPALNLVVTRARLLQCDPTHPGGMAIVGASQERILYIIHKLGLDD--RLVIAVYNDPENHVISGEIKAIDTFLSTANIWGFRGTKINVDQG--APCISSALPALQFVVERAKLLRADPTRPAGMAALQTTEARVAQYIQKLGVEG--RVAIAVYNAPDAHVVSGELKAVESVLAAAKRDGVRCTKLNVDQGFHSPAVASALPSLQFVVERAKLLRADPTRPAGMAALQTTEARVAQYIQKLGVEG--RVAIAVYNAPDAHVVSGELKAVESVLAAAKRDGVRCTKLNVDQGFHSPAVASALPSLQFVVERAKLLRADPTRPAGMAALQTTEARVAQYIQKLGVEG--RVAIAVYNAPDAHVVSGELKAVESVLAAAKRDGVRCTKLNVDQGFHSPAVASALPSL+FVV+RARLLR+DPT+PAGMAA++ASEERILQ+IHKLGLEDANRLAIAVYNGP+NHVVSGELKAIDS+LA+AK++GIRATKLNVDQGFHSP+I+SALP+LRAWSEKHISSARPLQIPLYSTLLGAQVSEGQMLNPDHWVDHARKPVQFAQAATIM-KESFTGVIIDIGPQVVAWSLLLSNGLT-SV-TALAAKRGRSQQVEAWLAGNQHELAPLKLPMYSTVYGRKIAADSELATSYWIEHAKNPVEFYRAVRELENDKDLNIILDIGPQPFIWTTLQTLQHH-KATISTSTKQPQSQNLEAWLAGNQHELAPLKLPMYSTVYGRKIAADSELATSYWIEHAKNPVEFYRAVRELENDKDLNIILDIGPQPFIWTTLQTLQHH-KATISTSTKQPQSQNLEAWLAGNQHELAPLKLPMYSTVYGRKIAADSELATSYWIEHAKNPVEFYRAVRELENDKDLNIILDIGPQPFIWTTLQTLQHH-KATISTSTKQPQSQNLQEWVSGHRHLSSPLNIPLYSTVYGKEIRGNQWLSPDYWVEHARDPVRFFDAVEALYSSKSFDIIVDIGPQPLIWTTLQSFSRK-NIAIATCGKRSNDQNAQEWVSGHRHLSSPLNIPLYSTVYGKEIRGNQWLSPDYWVEHARDPVRFFDAVEALYSSKSFDIIVDIGPQPLIWTTLQSFSRK-NIAIATCGKRSNDQNAQEWVSGHRHLSSPLNIPLYSTVYGKEIRGNQWLSPDYWVEHARDPVRFFDAVEALYSSKSFDIIVDIGPQPLIWTTLQSFSRK-NIAIATCGKRSNDQNAKMWLDNHDDAITGLEKPFFSTLRGAEIPKHERLDTQYWIAHAKSPVRFYETARVATKASSIDVIVDVGPQPTVWSNMQTPEYAGKARLAFTGKRGKDQIVKMWLDNHDDAITGLEKPFFSTLRGAEIPKHERLDTQYWIAHAKSPVRFYETARVATKASSIDVIVDVGPQPTVWSNMQTPEYAGKARLAFTGKRGKDQIVKMWLDNHDDAITGLEKPFFSTLRGAEIPKHERLDTQYWIAHAKSPVRFYETARVATKASSIDVIVDVGPQPTVWSNMQTPEYAGKARLAFTGKRGKDQIV+AWL+GH+H+++PL+IPLYSTVYGAEI+++Q+L+TDYWIEHAK+PVRFY+AVR+L+K+KS+DIIVDIGPQP+IWTTLQT++++GKA+IAT+GKRG+DQNVAFLSALADLYQDYGVVPDFVGLYAQQEDASRLKKTDILTYPFQRVRRYPSFIPSRRAP------------------------------------------AFLRGIALLFE-QGVTPNFEKLLQGS-R-SRGRKISVPTYPFQKQRHYPECIPSRYHVPK----------GSTVDRVLEFPIDQGLFDLLADHLIQGHRVAFLRGS----------------------------------------------------------------------------------------------AFLRGSGG--------------------------------------------------------------------------------------------AFLGAIASLFE-MDIAPDFGKLLAHKSQ-YGGHMCSLPTYPFQRQRHYPTSIPSRNSPPPVLLPLLPVSHTNPSSSIIHFNVDQSLCDLLLDHRIEGHRVAFLGAIASLFE-MDIAPDFGKLLAHKSQ-YGG--------------------------------------------------------------------AFLGAIASLFE-MDIAPDFGKLLAHKSQ-YGGS-------------------------------------------------------------------AMLAALSSLFE-KGFNVDFDALFSQM-P-YKFAMTDVPTYPFQRLYNYPAYICTRSSTVASILNQVET--SQQKAPTPQFVVDQSLCDFLDLHRIEGRRVAMLAALSSLFE-KGS-------------------------------------------------------------------------------------AMLAALSSLFE-KGSGGG----------------------------------------------------------------------------------Dehydratase domainAFL+A+ASLFED+GIAPDFGKLLAHKSQAYGG++T+VPTYPFQRQRHYP++IPSR+SPP+++L+++++SH+++++++++F+VDQSLCDLL+DHRIEGHRV----------------------------------------------------------------------------------------------------VPAAYLIDFFANKAPSGALQSISFHLPLVLESYDLTVNAEINGNGRFLLYNSDSSGQHVCSGILGSRVPPVLKNG-VRDQ---PPEQIKDTADVYASFKN-------------------------------------------------------------------------------------------------------------------------------------------------------------------G------------------------------------APGASLVDFFAKLCPVKSVKTIKFNAPLVLDFPEAHVVAEFTDGHHFAMYDNHSRTHIVCSGIAASRAPQSYTRQPINLNPSEQPEQVLTKDEIYKVFKN-----------------------------------------------------------------------------------------------------------------------------------------------------------------GG-------------------------------------LPGAAMVDFFARAAGSKSVKNVKFHTPLVLETPETQVRAEIDEQGAYKLVQDDGADTLICSGTISDKRGSSLGRK-VAHEPEAVPLQMMTKTQIYECFKN--------------------------------------------------------------------------------------------------------------------------------------------------------------------G-----------------------------------+PGA+LVDFFA++APSKSVK+IKFH+PLVLE+PE++V+AEI+++G+F+LY++DS++++VCSGI+GSR+P+SL+R+PV+++P+++PEQ++TK++IY++FKN----------------------------------------------------------------------------------------------------VQFGPSFRNVQEIRTWPTHADALIAVHSAAHRAPSLDRIRKLDACLHSLGAIITREVPQVRELDGAFLPSSLEGFSLHSDDLPESFVCRYYLPVDVARNY--------------------------------------------------------------------------------------------------------------------------------------------------------------------------------------------------------VEFGSAFRNIQEYRRWSSHADCLITVEPTEH--PAHDRIRKLDSCLHMFGAFSFQEVPQSRDLDGAFLPTALEDFTLHSDELPSSFICRYYLPLDVSRNF--------------------------------------------------------------------------------------------------------------------------------------------------------------------------------------------------------VQFGDPFRTVQAVRIWADYADADIRLEATAY--PAGDRIRKLDACLHMFGALSSRLAPPVDDNAGAYLPASLEDFTLHTDDMPYKFTCRYYLPLDIGRNA--------------------------------------------------------------------------------------------------------------------------------------------------------------------------------------------------------VQFG++FRNVQE+R+W++HADALI+VE+TAHRAPA+DRIRKLDACLHMFGA+S+REVPQVRDLDGAFLP+SLEDFTLHSDDLP+SF+CRYYLPLDV+RN+----------------------------------------------------------------------------------------------------HVISAAFDVFSEAGELLVSCKKYSVAWIPTGVTIQKSERNSGSTSEAQSVGWWRQTWVNKGTATE-DIGFSKYDQLLAISPTERDSRIASLANGIS------------------------------------------------------------------------------------------------------------------------------------------------------------------------------------------------------------HVMSAAFDVFSLAGALLVSCRKYSVAWIPVGIAIPNHI-S----QPSVNTQWLQQSWVARDLPSA--VPDDKLEVLCVID--QAQSQIPSLFNRMAWKT---------------------------------------------------------------------------------------------------------------------------------------------------------------------------------------------------------RVLSSCFEVFSDAGDLLVSCKKYSVAWVPKGVVHKEQK-P----QQTAPDTWIRNAWTTQNLPAPQTTSVHRFDEIIYFGNGETSRVLSSLSSSAKNCIS--------------------------------------------------------------------------------------------------------------------------------------------------------------------------------------------------------HV+SAAFDVFS+AG+LLVSCKKYSVAWIP+GV+I++++R+SGSTQ++++++W+RQ+WV+++LP++Q+++++K+D+L++I+++E++S+I+SL+N++++++S----------------------------------------------------------------------------------------------------------------------------KEVSLP---DPLEEVIQKFKDSPAVIMLDLTSIDALPMTEAFTSCYRHVLRLMQLLSSHKIDIREFVVISATSVAA--------------------------------------------------------------------------------------------------------------------------------------------------------------------------------------------------------------------HFLNLYDLLAPSQSFSLPSLSLQLHAIIDRITSTNLLIVVDVTSNHASPTSEAFCSSHRHILTLMKLLISSKVRFTSLVFITEMSVAI--------------------------------------------------------------------------------------------------------------------------------------------------------------------------------------------------------VEMPHLPRDESGKIPNN-------TKVVSP---EDMNKLPAILRGQDMLVVLDLSKSNNSPGSDQFTALYLQALMFLKHIMSHKFHISSFLALTSWSAPV--------------------------------------------------------------------------------------------------------------------------------------------------------------------------------------------------------VEMPHLPRDESG+++N+YDLLAPS++VSLPSLS++L+++I++++++++LIVLDLTS++ASP+SEAFTS+YRH+L+LMKLL+SHK++I+SFV+IT++SVA+----------------------------------------------------------------------------------------------------DVPA------GPSAPEVEAPSVAALVQGMMRVFRRETGSDDQIWALDLPRLDTISDDALRHLLLSELEGRQRGKHTDRAVAYRRRDGQEKVERLVPIFES--------------------------------------------------------------------------------------------------------------------------------------------------------------------------------------------------------GNEG--NRLLAPHLLASMTPTVGSVIQGMLRVFRREMGLDEVIWALDLPPMNTVEDGVILGIISNEICSRLHGLSTDRTVAYRNVDQTKSLSRLVPVLQS--------------------------------------------------------------------------------------------------------------------------------------------------------------------------------------------------------DLYKEGLDLFSDSKVS-SASLVGAVVQGMIRVFRRETGLDFAAWCLDLTSIDSLTDSQLQNILTSEIQARYRSEFLDTFVCYREDADKKSLSRLVPSLES--------------------------------------------------------------------------------------------------------------------------------------------------------------------------------------------------------D+++EG++L++PS+++++AP+VGAVVQGM+RVFRRETGLD++IWALDLP++DT++D++L++IL+SEI++R+RG++TDR+VAYR++D++KSLSRLVP+LES----------------------------------------------------------------------------------------------------VKADEEVGTTYHGVAVITGLGSIGASLAQPMVMKGSSKVVYIGRRPVDDTEVQTTLHRLESEIPGRIAYVQADVCELDSLKSAISDIQASHGPIGSIIHS-------------------------------------------------------------------------------------------------------------------GSGGGGSG-----------------------------------------------------------------------------IGHH--PIREVSGTSIVVGLGSIGHALAPSLA-GSHSQVVFIGRRQAHDHEVQEVLLHLQSKTGGRCAYMQADVCDPDSLRNVIISAQTLYGPIENIVHT------------------------------------------------------------------------------------------------------------------GGSGGGGSGG----------------------------------------------------------------------------LERV--PARTPSGTTVIVGMGSIGTALAASLVEVGCNPVIFFGRRPDSQEKVVNELSALPENVRKQCQYRQVDVCDMEALKKALADVNATHGGIKNIIHT--------------------------------------------------------------------------------------------------------------------SG----------------------------------------------------------------------------------Ketoreductase domain++++EEP+RT+SGT++SGG+GSIG+ALA+SLV++G+S+VVFIGRRP++D+EVQ++L++L+S+++GRCAY+QADVCD+DSLK+AI+D+QA+HGPI+NIIHT----------------------------------------------------------------------------------------------------AAVISDATIQNIDLQSFETVIRPKVVGAWNLHVLNEELCPTLADFVLLSSISVSLGNPGQAAYAAANHYIEVLASYRRGKNLPATALQLGPWESKLTQNL--------------------------------------------------------------------------------------------------------------------------------------------------------------------------------------------------------AVVVSDATIQTVADKSFELVLRPKVIGAWNLHTICEELKLPLKSFVLLSSVSVPLGNQGQIAYVAGNAYMETLASYRHSVGLPATCLQLGAWESKLVQNL--------------------------------------------------------------------------------------------------------------------------------------------------------------------------------------------------------AAVVTDSTIVATKPSDFEAVLLPKVTGSWNLHVASQELNLALDSFVLFSSTNVIVGNPGQVSYVAANSFMDSLATFRHNCGLPGASLQLGAWESRLISDV---------------------------------------------------------------------------------------------------------------------------------------------------------------------------------------------GGGSGGGGSDVAAVVSDATIQ+++++SFE+VLRPKV+GAWNLHV++EEL+L+L+SFVLLSS+SV+LGNPGQ+AYVAAN+YME+LASYRH++GLPAT+LQLGAWESKL++++--------------------------THAHVQDEETLSSGSSTPTLENTD-----LDSGKESLMG-PTRGLLRVDDLRDSIVSSVKDVLELKSNEDLDLSDTRDSLIPLMNNKRGVPLIISAMSKSDSVQMIANLDARKLAAHPVYSRDPLFYDIVFPEQDRLAK-SHQAR-SDEEISTLVANILRKVLELRPSEKLGQS-----------------------------------------------------------------------------------------------------GGGSLIPLMNNKRGVPLIISAMSKSDSVQMIANLDARKLAAHPVYSRDPLFYDIVFPEQDRLAK-SHQAR-SDEEISTLVANILRKVLELRPSEKLGQSDFSTGFVRPIKHAKGIPLLLKAMLTPIAVQVIADFDVEKLASVPAYARDPLFCHILGGAALTQIKTSLRGNLTEDEVADIMIDILRTVLELRPSERLG------------------------------------------------------------------------------------------------------GGSTGFVRPIKHAKGIPLLLKAMLTPIAVQVIADFDVEKLASVPAYARDPLFCHILGGAALTQIKTSLRGNLTEDEVADIMIDILRTVLELRPSERLG--NMENSFALLMKNDEGLPLILKAMMAPIPLQIIARMDTSKLSANPAYAKDPFFAPLLSSSNGAAPK-DTKAKLSKENAQKILIDILRVALELQPSEKLDTS----------------------------------------------------------------------------------------------------NMENSFALLMKNDEGLPLILKAMMAPIPLQIIARMDTSKLSANPAYAKDPFFAPLLSSSNGAAPK-DTKAKLSKENAQKILIDILRVALELQPSEKLDTSACP++++SF++LMKN++G+PLILKAM++PI+VQVIA++D+SKLAA+PAYARDPLF++IL+S++++L+KTSTRA+LS+EE++DI+IDILR+VLELRPSEKLG+S------------------ESLNALGMDSIMFAQLRKRIGEGLGLSVPMVFLSDAFSIGEMVSNLVEQAEASEDN-LNLLLLAQLFDNRTAELDDSLTICGVDSISFAQVRGRILHELMVEVPMMFLSDTFTIHEMIAFVIEKYSSRIASL---------------------------------------------------------------------------LNLLLLAQLFDNRTAELDDSLTICGVDSISFAQVRGRILHELMVEVPMMFLSDTFTIHEMIAFVIEKYSSRIASL---------------------------------------------------------------------------------------------------------------------------------------------------------------------------------------------------------------------------------------------------EELTALGADSITFAQFKGQVLKEFAVDVPMVYLSDGYTISDMINNVLESYG--VASL---------------------------------------------------------------------------------------------EELTALGADSITFAQFKGQVLKEFAVDVPMVYLSDGYTISDMINNVLESYG--VASLLNLLLLAQLFDNRTAELDESLTALG+DSI+FAQ+RGRIL+EL+V+VPMVFLSD+FTI+EMI+NV+E+Y+SR+ASL111111111198949494100100100979797102030405060708090999595951011011019898981961931931932002002001971971971101201301401501601701801901971941941942012012011981981982832932932932932932932862862862102202302402502602702802902842942942942942942942872872873753873873873803803803713713713103203303403503603703803903763883883883813813813723723724734814814814764764764654654654104204304404504604704804904744824824824774774774664664665695815815815765765765555555555105205305405505605705805905705825825825775775775565565566446756756756676676676436436436106206306406506606706806906456766766766686686686446446447347747747747647647647427427427107207307407507607707807907357757757757657657657437437438338748748748648648648428428428108208308408508608708808908348758758758658658658438438439339739739739639639639419419419109209309409509609709809909349749749749649649649429429421033107110711071106110611061104010401040101010201030104010501060107010801090103410721072107210621062106210411041104111221169116911691161116111611134113411341110112011301140115011601170118011901123117011701170116211621162113511351135121912691269126912591259125912311231123112101220123012401250126012701280129012201270127012701260126012601232123212321319136913691369135913591359133113311331131013201330134013501360137013801390132013701370137013601360136013321332133214191467146714671455145514551429142914291410142014301440145014601470148014901420146814681468145614561456143014301430151615661566156615541554155415291529152915101520153015401550156015701580159015171567156715671555155515551530153015301574165315721574165215841585162415431546161016201630164016501660167016801690165415751653158616251547174915751752158717231547171017201730174017501760177017801790175017531724184918501821181018201830184018501860187018801890185018511822194419401916191019201930194019501960197019801990194519411917201720282006201020202030204020502060207020802090201820292007211121262105211021202130214021502160217021802190211215762127158821061548221115832223159722031549221022202230224022502260227022802290221222242204155023112323230315602310232023302340235023602370238023901575231215842324159823041561164224091680242116952402165924102420243024402450246024702480249016432410168124031660169824841755245717142510252025302540255025602570
