# Supplementary material for: Domain Truncation in Hispidin Synthase Orthologs from Non-Bioluminescent Fungi Does Not Lead to Hispidin Biosynthesis
Source: Int J Mol Sci. 2023 Jan 10;24(2):1317. doi: 10.3390/ijms24021317 (PMC9866795; doi:10.3390/ijms24021317)
Supplement: Supplementary file 1 [file ijms-24-01317-s001.zip › Supplementary materials.docx]

**Supplementary materials**


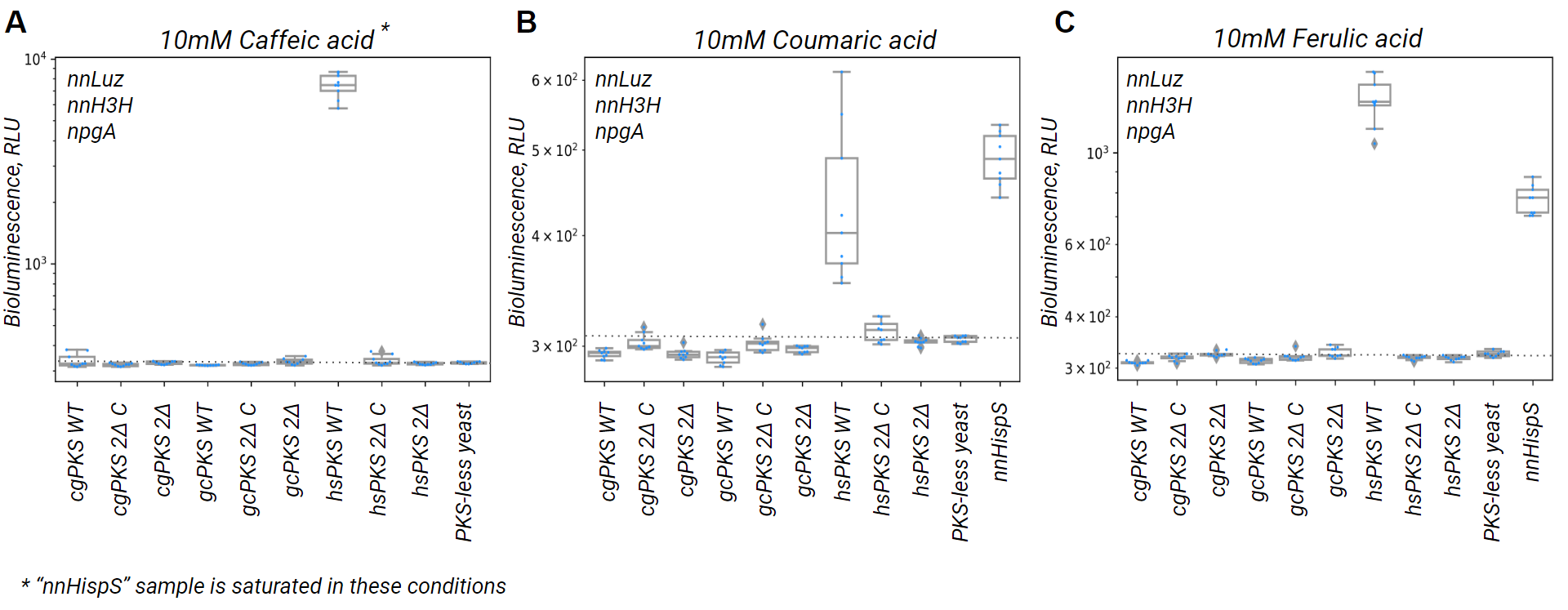


**Supplementary Figure 1.** Luminescence (Fusion Pulse.7 (BioRad), exposure time – 30 minutes) emitted by *Pichia pastoris* GS115 yeast colonies expressing nnLuz, nnH3H, npgA, and full-length or truncated polyketide synthases from *Cortinarius glaucopus* (cgPKS), *Hypholoma sublateritium* (hsPKS), and *Gymnopilus chrysopellus* (gcPKS) or nnHispS after treatment with 10mM caffeic acid (**A**), 10mM coumaric acid (**B**), 10mM ferulic acid (**C**).


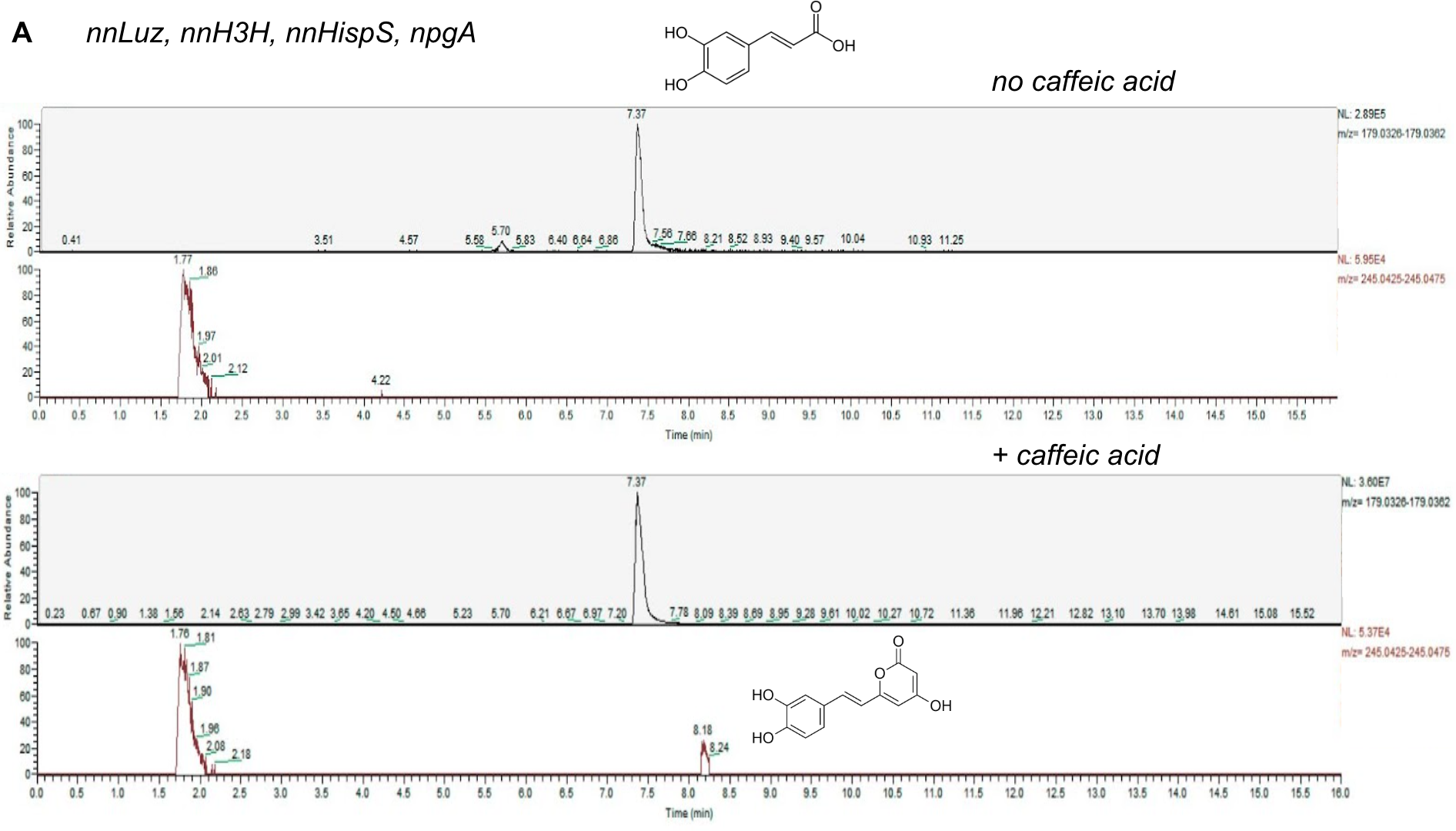

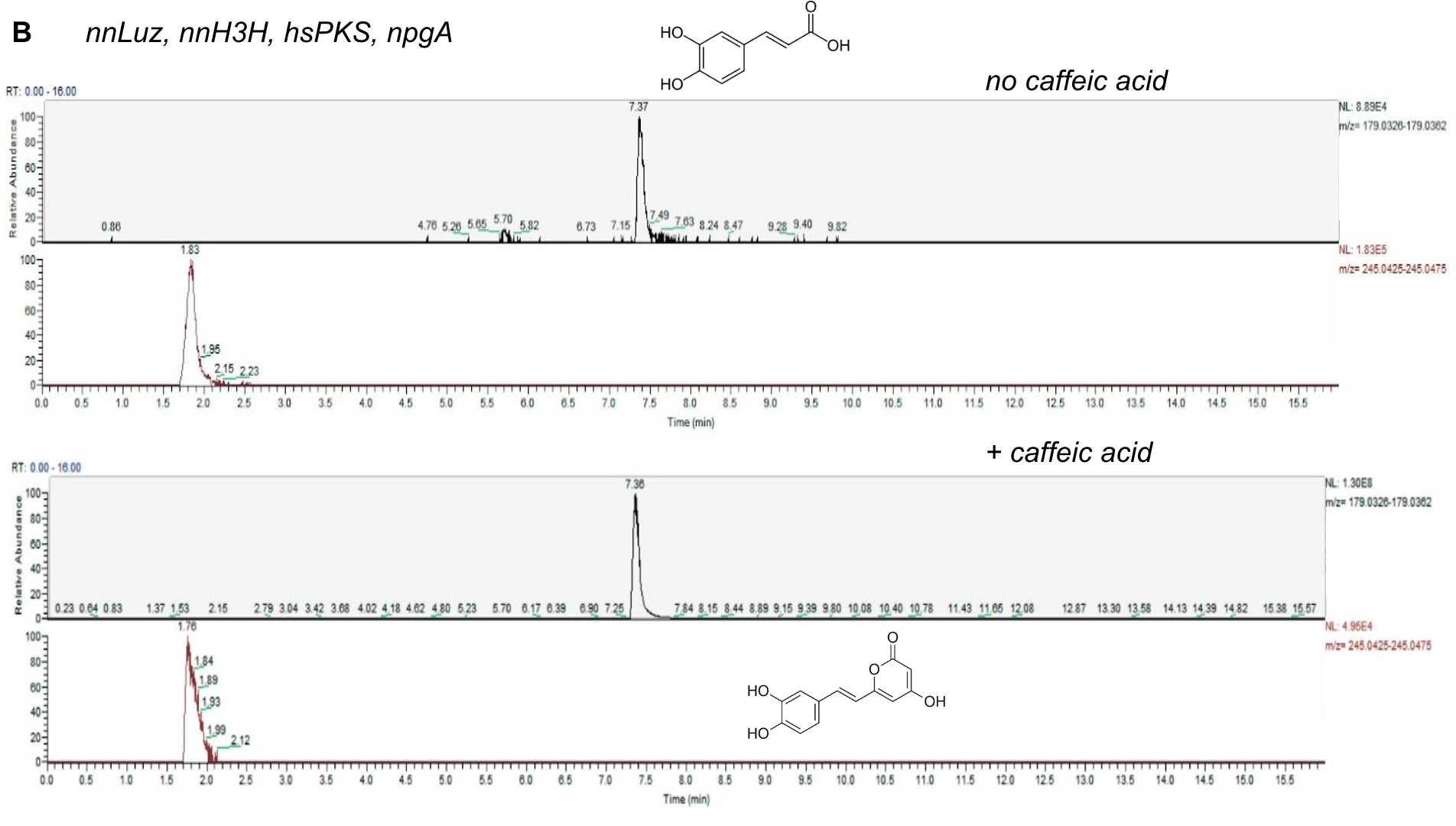

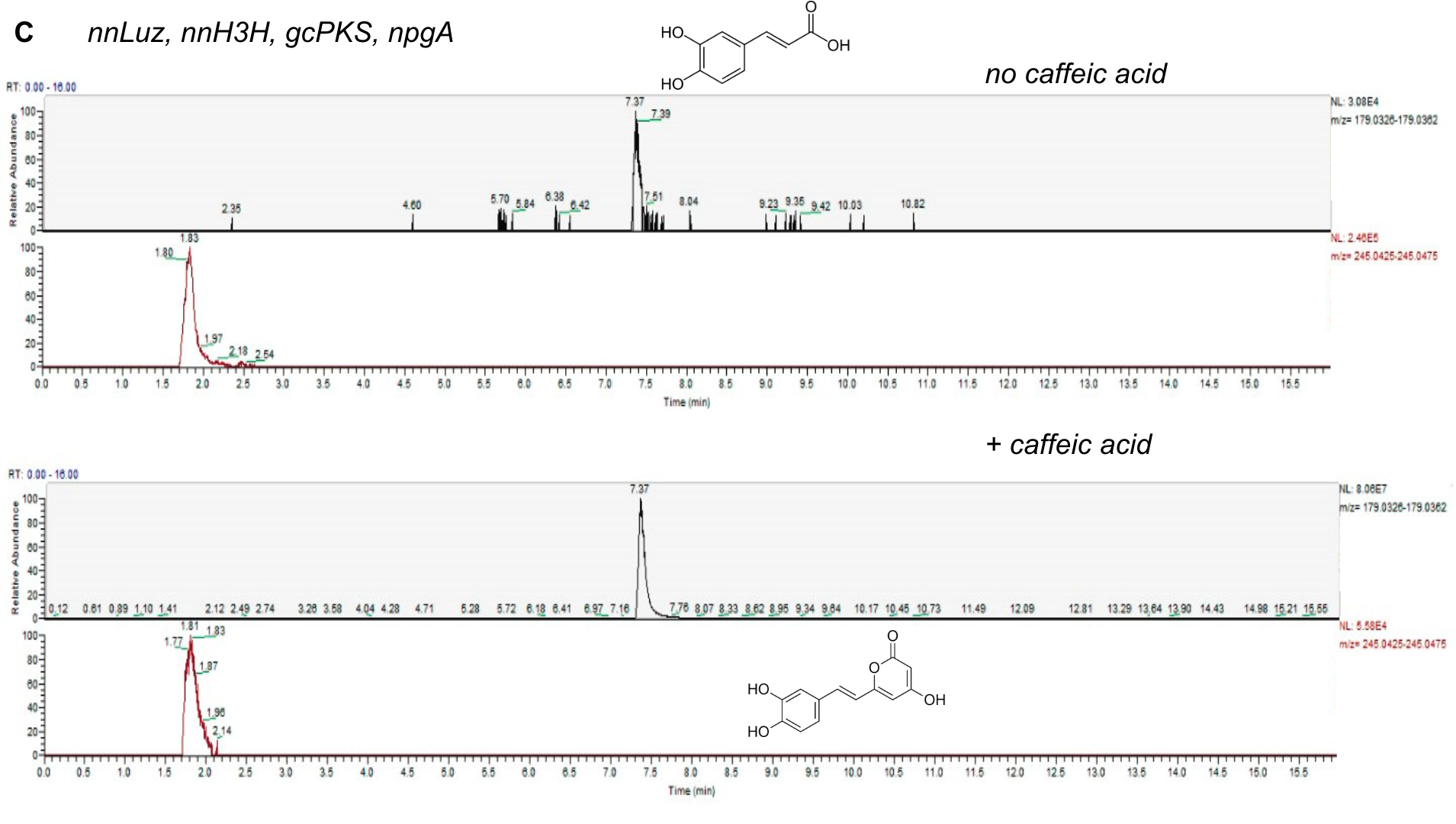

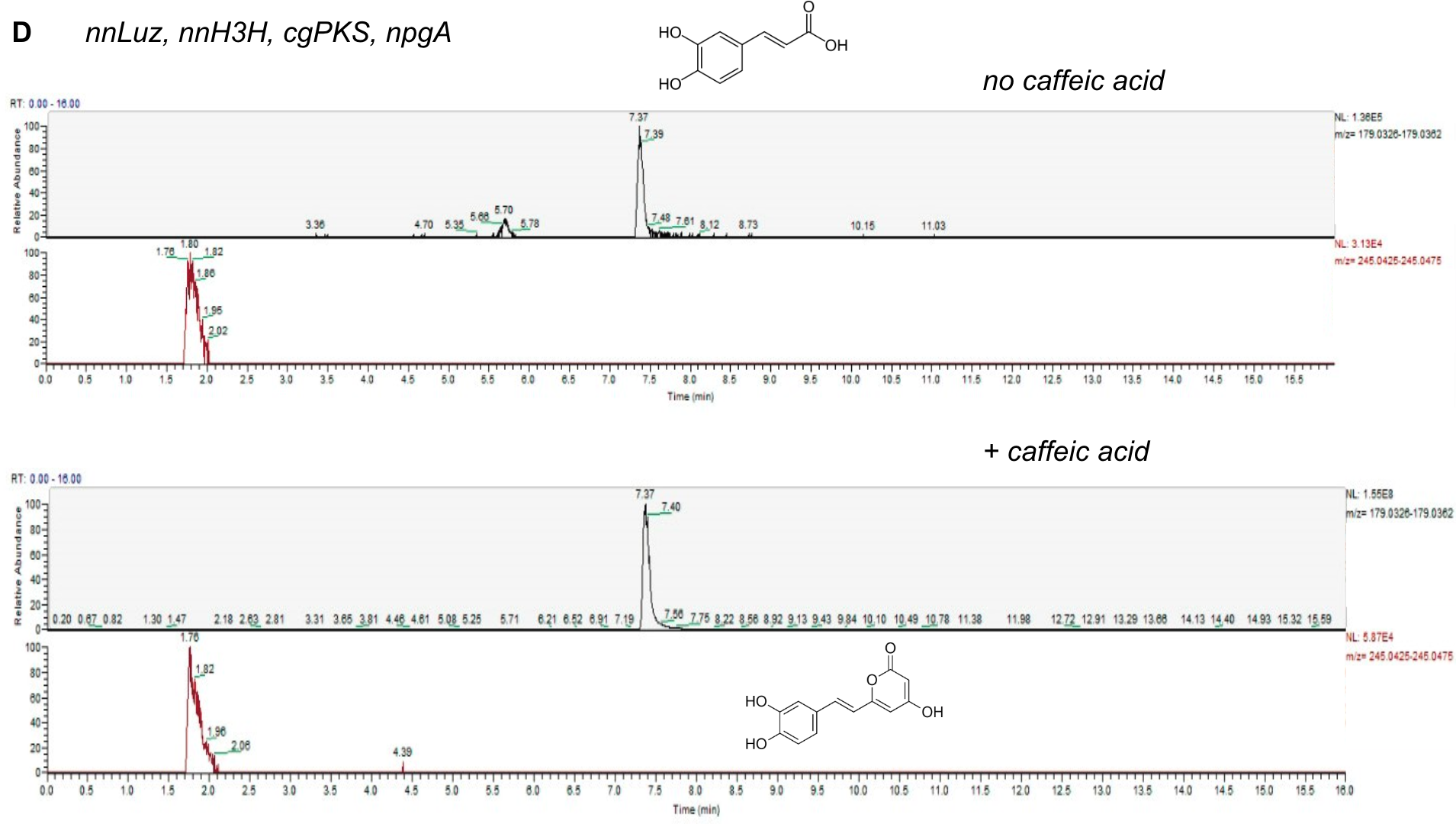

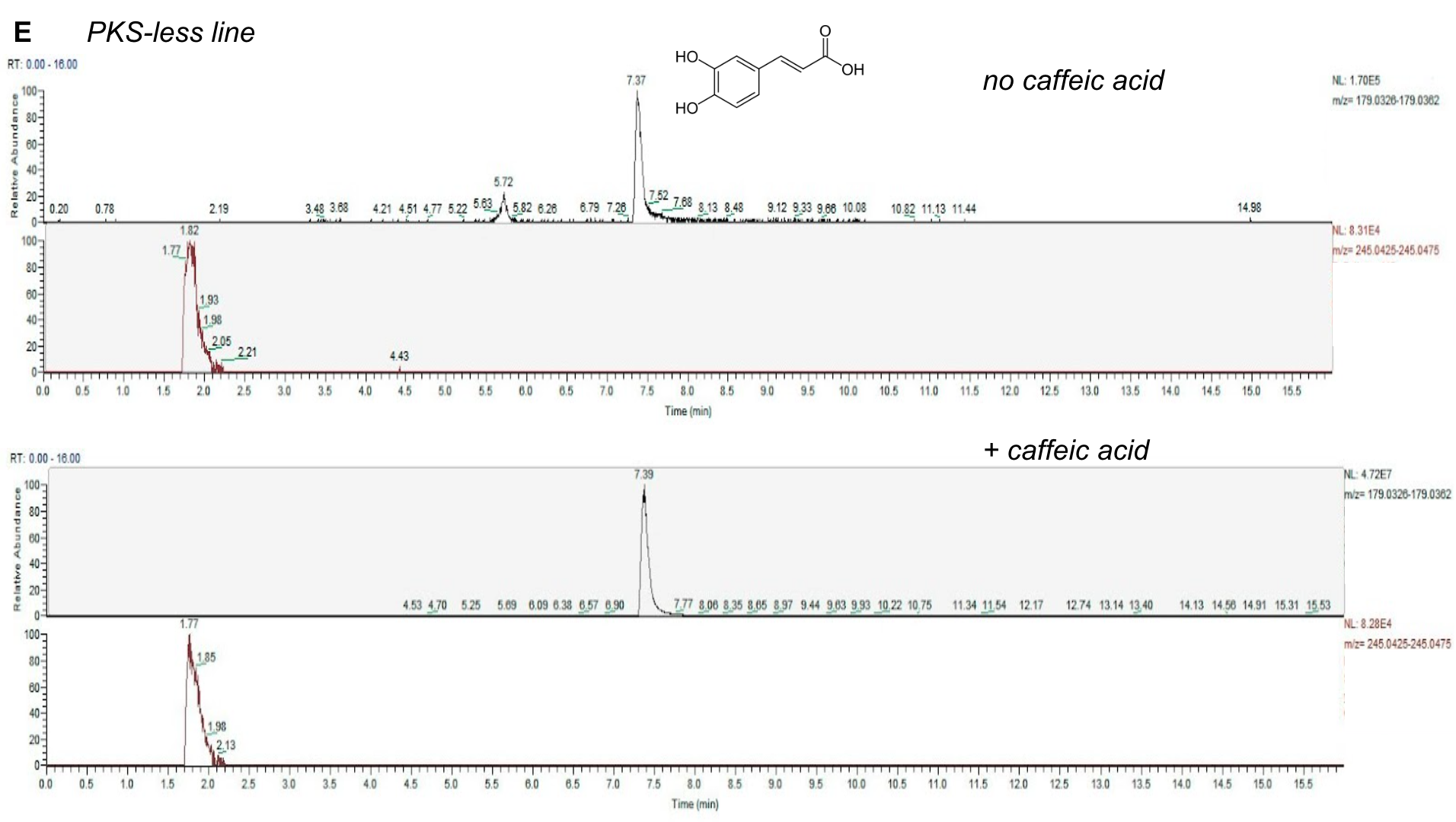

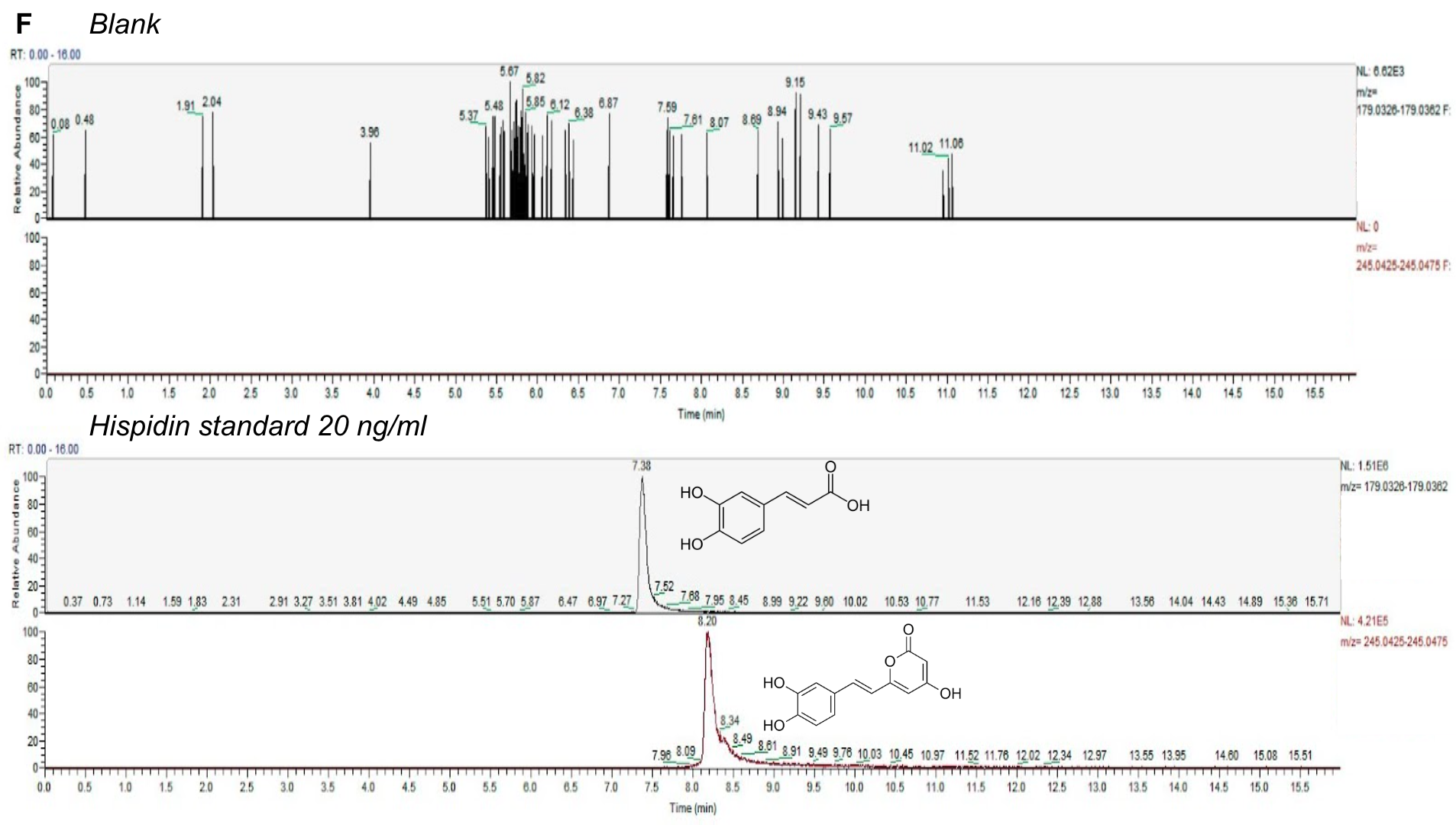


**Supplementary Figure 2**. LC-MS spectra of extracts of *Pichia pastoris* GS115 grown in the presence or absence of caffeic acid (10 mM) and expressing nnLuz, nnH3H, npgA, and either **(A)** nnHispS, **(B)** full-length polyketide synthase from *Hypholoma sublateritium* (hsPKS), **(C)** full-length polyketide synthase from *Gymnopilus chrysopellus* (gcPKS), **(D)** full-length polyketide synthase from *Cortinarius glaucopus* (cgPKS), or **(E)** no PKS (control strain). **(F)** Samples used as references in the analysis: blank sample and chemically synthesised hispidin (20 ng/ml).

**Supplementary Table 1.** The results of RT-PCR of assayed polyketide synthases. The data represents the values of ∆Ct = (Ct (assayed gene) – Ct (actin 1)) - Ct(nnHispS).

hsPKS WT 0.173333

hsPKS 2D -1.426667

hsPKS 2D_C 0.683333

gcPKS WT 0.240000

gcPKS 2D 0.583333

gcPKS 2D_C 0.420000

cgPKS WT 4.406667

cgPKS 2D -2.820000

cgPKS 2D_C 0.210000

**Supplementary Table 2. Primers used in the study.**

| **Primer name** | **Primer sequence** |
| --- | --- |
| hsPKS part1 RT dir | CTGGATAGCACACGCAAAGA |
| hsPKS part3 RT dir | GAAAATGCCCAGAAAATTTTAATC |
| cgPKS part1 RT dir | CCGTCAGATTTTTCGATGCAG |
| cgPKS part3 RT dir | TGTTAAAAGCTATGCTTACTCC |
| gcPKS part1 for D2 RT dir | CGTTTATGGACGTAAAATTGCT |
| gcPKS part3 RT dir | GTGAAAAGTTGGGCCAGTCT |
| tAOX rev | GCAAATGGCATTCTGACATCC |
| Actin forw | GGTGTGGTGCCAGATCTTTT |
| Actin rev | AGTGTTCCCATCGGTCGTAG |
| Pich actin forw | GGTGTGGTGCCAGATCTTTT |
| Pich actin rev | AGTGTTCCCATCGGTCGTAG |
| nnHispS_4950nt_ dir | GGGATGGACTCCATCATGTT |
| nnHispS rev | GTTGTCCTCGGAAGCCTCA |
| Hipsu PKS part3 RT rev | AGCGACTCCATAACTCTCTAACACGTTA |
| Hipsu PKS part3 RT dir | AGGGTTGCCTTAGAGCTTCAGCC |
| Hipsu PKS part1 RT rev | CTCAAATAGACTTGACAAAGCAGCAAGCA |
| Hipsu PKS part1 RT dir | ATACTGGATAGCACACGCAAAGAGTCC |
| Corgl PKS part3 RT rev | ACCAAGACGCTCGGATGGCC |
| Corgl PKS part3 RT dir | GCTTACTCCCATAGCTGTTCAAGTCATCG |
| Corgl PKS part1 RT rev | GTACTGACTCTTGTGTGCCAACAACTTAC |
| Corgl PKS part1 RT dir | CCTCAGCCATTGATATGGACTACATTGC |
| Gymch PKS part3 RT rev | GCTATCCTACTACTATACTTTTCGATTACAAAAGCAATC |
| Gymch PKS part3 RT dir | GGATAGATTAGCTAAGAGTCATCAGGCTCG |
| Gymch PKS part1 RT rev | GCAAGATTCTGTGACTGAGGTTGCTTG |
| Gymch PKS part1 RT dir | CCTTCCTGTGTTAGAAGCCTGGCTAG |

**Supplementary Table 3. Plasmids used in the study.**

| **Constructs name** | **Constructs sequence** |
| --- | --- |
| pGAP - hsPKS WT - tAOX | <https://benchling.com/s/seq-TuIZsuIdI7K6YngJaYuD?m=slm-a5tTbM9ovSZijyEWniok> |
| pGAP - hsPKS 2Δ_C - tAOX | <https://benchling.com/s/seq-AJsT8MAio0kicPRIbzmG?m=slm-OKsy3dH7HKUj1SIlc7uv> |
| pGAP - hsPKS 2Δ - tAOX | <https://benchling.com/s/seq-VuK85cbYVnyyRDPTYcDL?m=slm-ANyLyYfQN5IF16ONhSug> |
| pGAP - gcPKS WT - tAOX | <https://benchling.com/s/seq-7nGLItIAJ8Jr9YHjYHQt?m=slm-Ob2AQmWP4DRb3Ntw94gw> |
| pGAP - gcPKS 2Δ_C - tAOX | <https://benchling.com/s/seq-SSvx2tXEY5lSxuzdAZMs?m=slm-xGGaFcgexSOKZV5rTyHF> |
| pGAP - gcPKS 2Δ - tAOX | <https://benchling.com/s/seq-AJQ6tf8cLnlBl7LLfSxz?m=slm-Lso4BL3LSXYGJc5dBgjc> |
| pGAP - cgPKS WT - tAOX | <https://benchling.com/s/seq-NL74aiWHvMC5adlV2WBJ?m=slm-LKepp7cJJCXDzAbZeOLd> |
| pGAP - cgPKS 2Δ_C - tAOX | <https://benchling.com/s/seq-GXfE3gr5gw6vrrWNYuNR?m=slm-TYK6HitbPNr1oF9NFA6d> |
| pGAP - cgPKS 2Δ - tAOX | <https://benchling.com/s/seq-2Xy6qcfAQIC238Gr5zik?m=slm-dBp4hZtBkcI8sP0PmBh4> |
| pGAP - nnHispS - tAOX | <https://benchling.com/s/seq-jEJ17iy4tBR0aMAVoDDw?m=slm-MhQZOeqAxDrAAyqHYXPZ> |
